# Role of recruitment bias in stepped-wedge cluster randomised controlled trials: a systematic review

**DOI:** 10.1136/bmjopen-2024-096281

**Published:** 2025-11-28

**Authors:** Antonina Yakimova, Fraser Wiggins, Mona Kanaan, Ada Keding, David Torgerson

**Affiliations:** 1 Department of Health Sciences, University of York, York, UK

**Keywords:** Randomized Controlled Trial, Systematic Review, STATISTICS & RESEARCH METHODS

## Abstract

**Abstract:**

**Objectives:**

Increased popularity of stepped-wedge cluster randomised trials (SW-CRT) highlights the importance of understanding and appropriate mitigation of sources of bias within this trial design. While current evidence suggests that ‘conventional’ cluster randomised controlled trials (RCTs) are at a higher risk of recruitment bias than individually randomised trials, this review aims to estimate the risk of recruitment bias in SW-CRTs.

**Design:**

Systematic review with search conducted on four databases. Risk of bias (RoB) was assessed using subdomain 1a (randomisation process) and 1b (timing of identification or recruitment of participants) of the Cochrane RoB tool 2.0 (extension for cluster RCTs).

**Data sources:**

MEDLINE, Embase, CINAHL, Cochrane Library were searched on 9 February 2024.

**Eligibility criteria for selecting studies:**

SW-CRTs published in 2023 were included.

**Data extraction and synthesis:**

Two independent reviewers screened and extracted all eligible papers. RoB was assessed with the Cochrane RoB tool.

**Results:**

Overall, 808 papers were screened, and 64 studies were included in the review. Most studies were deemed to have a high RoB (n=35, 55%), some concerns were noticed in 20 studies (31%), and 9 (14%) were considered to have a low RoB. The description of the randomisation process in the included papers was sometimes poorly reported (in 15 studies (23%) problems with the randomisation process were identified), and 21 studies (33%) had issues with sampling strategy (recruiting participants after randomisation by unmasked staff).

**Conclusions:**

The review revealed that SW-CRTs are prone to recruitment bias, but the risks are comparable to cluster RCTs. When SW-CRTs are unable to recruit prior to randomisation, mitigation strategies could be implemented to reduce bias. A separate tool for RoB assessment in SW-CRTs is required to address the complexities of this trial design.

**STRENGTHS AND LIMITATIONS OF THIS STUDY**

- The study used systematic review methodology and incorporated a comprehensive literature search of four electronic databases, resulting in a diverse sample of journals, countries and interventions, and therefore, enhancing the generalisability of the review’s results.
- The review used an a priori agreed systematic evaluation, utilising the standard framework of the Cochrane risk of bias tool, to identify common shortcomings in the reporting of recruitment practices in recently published stepped-wedge cluster trials and to highlight effective mitigation strategies used in these trials.
- Only studies published in 2023 were included in the review, and although there were no language limitations, the dominant language in all included papers was English.
- As there is currently no custom tool available to quality assess the stepped-wedge cluster trial design, the generic Cochrane risk of bias tool may not fully capture design-specific risks.

## Introduction

 Stepped-wedge cluster randomised trials (SW-CRTs) are one of the more complex designs among randomised controlled trials (RCTs).^1^ Randomisation in SW-CRT occurs at a cluster level rather than at an individual level (eg, hospitals or schools are randomised instead of individual patients or children). The study design also aims to measure outcomes for each cluster before and after the exposure to the intervention at different time points.^1^ Each cluster or a group of clusters is allocated to a different sequence with a different number of periods spent in control and intervention conditions.^2^ Outcome data in SW-CRTs can be obtained from different participants at each study step (cross-sectional design), from the same individuals (longitudinal data from closed cohort design), or from their combination (open cohort).^3^ In trials with brief exposure periods, continuous recruitment of participants could be considered as an alternative to more conventional cohort designs.^4^

The popularity of SW-CRTs has soared over the last 5 years (based on the number of search results for ‘stepped-wedge cluster’ on PubMed,^5^ date of search 25 March 2024, year of publication (number of papers): 2006 (2), 2016 (76), 2020 (176) and 2023 (229)). SW-CRTs are commonly used for the assessment of pragmatic health policy interventions,^6^ particularly when due to policy, political or ethical reasons, such that it is deemed necessary for all clusters to receive the intervention. Since the outcomes of these trials affect the direction and implementation of healthcare standards, the results of such trials should be robust and unbiased. However, the complex study design and often continuous recruitment during both study conditions can lead to various sources of bias, such as awareness of the recruitment team of the allocation status and therefore selective recruitment of participants. One particular source of bias relates to the differential identification and recruitment of participants, which might lead to systematic differences between groups (intended or unintended selection of participants between or within clusters^7^). Although imbalances between intervention and control groups can also be problematic in a two-arm parallel CRT, where each cluster is allocated to either control or intervention group, imbalances between clusters and sequences are an additional area of concern in the stepped-wedge cluster design with individual recruitment.^1 6 8^

Another reason for concern is that the robustness of the trial results might be hindered, as certain crucial elements of RCTs are likely to be infeasible or impractical in SW-CRT (eg, recruitment of all participants prior to the randomisation in cross-sectional designs). The results of recent research emphasise the potentially high risk of differential recruitment in SW-CRT. The scoping review by Nevins *et al*^9^ revealed the high level of baseline imbalances reported in SW-CRTs, which can be an indication of both significant issues with a randomisation process (eg, use of a small number of clusters and unrestricted allocation) and possibility of selective recruitment.^10^ It is worth mentioning that simple (unrestricted) randomisation can guarantee balance on baseline characteristics only on the cluster level, but not on the individual (participants) level, particularly when the sample size is small. The balancing of characteristics on an individual level would require the use of restricted randomisation.^7^ Moreover, since current evidence suggests that cluster RCTs are at a higher risk of recruitment bias in comparison to individually randomised trials,^11^ the risk of bias (RoB) within SW-CRTs might be even greater. The aim of this review is to estimate the risk of recruitment bias in recently published SW-CRTs.

## Methods

This systematic review was conducted in accordance with established methodological principles and reported following the Preferred Reporting Items for Systematic Reviews and Meta-Analyses (PRISMA) guidelines.^12^ The complete PRISMA checklist is provided in online supplemental material file 1.

In this review, we use terms ‘recruitment bias’ and ‘selection bias’ as per the following descriptions. Although selection bias is generally considered to be a broader term,^2 13^ we deemed recruitment bias in this review as a broader term, addressing any issues arising from the randomisation process and the timing of identification or recruitment of participants (ie, any bias identified with Cochrane Risk of bias 2.0 tool (subdomain 1a and 1b)).^14^ Selection bias in this review is defined as issues arising from differential identification and recruitment of potential participants in clusters (ie, signalling question 1b.2 in subdomain 1b),^14^ for example, if recruiting staff and/or potential participants are aware of allocation status. Such awareness is likely (either consciously or subconsciously) to affect the recruitment process^14^ (eg, recruit healthier patients in the control condition, and patients with multiple comorbidities during the intervention condition). Therefore, in this review, selection bias is deemed one of the components of recruitment bias. Such an approach was chosen as sometimes participants are not identified and/or recruited before randomisation of clusters, and selectivity in identification and/or recruitment can become an element in the wider recruiting process. We acknowledge that these terms are sometimes used differently in the broader methodological literature^2 13^; however, for the purposes of this review, we adopted the above framework for clarity in the context of CRTs. See RoB assessment for further information.

### Patient and public involvement

No public or patient involvement was included in this review of published manuscripts.

### Eligibility criteria

Trials with stepped-wedge cluster randomised design were included. There were no limitations on included population groups, types of intervention or settings. As it was anticipated that the quality of reporting trials continues to improve over time,^2 15^ the publication date was limited to one calendar year due to the significant increase in annual number of registered stepped-wedge trials since the Hussey and Hughes paper in 2007.^16^ Based on a PubMed database search,^5^ since 2021, over 200 newly published papers that use the design were added annually. Therefore, we focused our review on the calendar year 2023 (the most recent complete calendar year at the time of data retrieval). Furthermore, it was assumed that articles published in that year would potentially be conducted and reported to higher standards than earlier years due to the increased awareness and use of the Consolidated Standards of Reporting Trials (CONSORT) statements partly driven by high impact journals consistently insisting on the reporting of RCTs to these guidelines.

There were no language limitations initially while searching, but all studies in languages other than English would be excluded if they could not be translated into English. Observational studies and animal studies were excluded from the review, as well as study protocols, ongoing and feasibility studies. Identical results of the same study related to the recruitment and randomisation process reported in separate papers were merged, while the papers with different settings or population groups were analysed separately (see Data selection and extraction).

### Information sources and search strategy

Searches for published materials (both peer-reviewed and preprints (ie, reviews available online but not being peer-reviewed yet)) were conducted in four electronic databases (MEDLINE (Ovid), Embase (Ovid), Cochrane Library (Wiley, 2024, Issue 1) and CINAHL Ultimate (EBSCO)). The search strategy for literature searches of electronic databases was developed with use of medical subject headings initially for MEDLINE and later adapted for other databases (online supplemental material file 2). All papers meeting the inclusion criteria (SW-CRT design and 2023 publication year) were included. Neither grey literature search nor citation searching was conducted.

### Data selection and extraction

The process of data screening and extraction was performed in Covidence Software (2024) (https://www.covidence.org/). All articles identified via the literature search were transferred directly to Covidence.

A team of two reviewers (AY and FW) independently screened the titles and abstracts, deciding on the eligibility of studies based on inclusion and exclusion criteria (online supplemental file 3, table S1). Members of the wider team were consulted in order to resolve any discrepancies. Reviewers were not masked either to study authors and their institutions or to the journals’ titles. During the full-text review stage, all papers were also double-screened (AY and FW) against inclusion criteria (online supplemental file 3, table S1).

If a study had reported results across several papers, all of them would be included in the review in the first instance. However, if these papers reported identical study characteristics (eg, population and other settings), then the papers were merged.

The extraction form was reviewed by all members of the team. Pilot extraction was conducted by one reviewer (AY) prior to the main extraction process in order to reveal inconsistencies and previously unreported issues and included three random eligible studies. Data extraction was performed independently by two reviewers (AY and FW). A calibration exercise with two included studies took place before the extraction process to confirm the practicality of the extraction form and ensure that both reviewers extract data in the same way and deal similarly with missing data. During data extraction, any disagreements between the reviewers were solved by discussion, and a third reviewer would be asked to be an arbitrator for any unresolved disagreements to reach a consensus.

The following data were extracted from all included studies: (1) general study information (title, country in which the study was conducted, authors, year, journal), (2) intervention characteristics (type and description of the intervention, study settings), (3) population characteristics (method of participants’ recruitment and its description, age group (adults and/or children as it was reported by the authors or, when not reported, adults were considered ≥18 years old)) and (4) quality assessment (RoB) (online supplemental material file 3, table S2).

As part of quality evaluation, we extracted information about the type of randomisation (eg, simple, blocked, stratified; signalling question 1a.1 as per the RoB assessment domains detailed below) and whether experienced independent personnel (eg, a statistician) were responsible for randomisation sequence generation (signalling questions 1a.1 and 1a.2). We also extracted any mitigation strategies that were reported by the authors to avoid bias arising from identification and recruitment of participants in clusters (signalling questions 1b.1 and 1b.2), such as use of waiver of consent or retrospective medical records. To assess signalling question 1a.3 (individual-level baseline imbalances), we judged whether authors adjusted their analysis models for imbalanced baseline covariates. Such adjustments could have been either specified a priori, for all clinically meaningful covariates, or added in post hoc analysis.

### RoB assessment

The current version 2.0 of the Cochrane Collaboration tool for assessing the RoB (RoB 2.0) for cluster RCTs^14^ was used. The tool incorporates five domains. Domain 1 includes assessment of bias arising from randomisation process and identification and recruitment of participants in a cluster RCT and was selected for this review for assessment of recruitment bias. The other four domains assess other sources of bias (deviation from intended interventions, missing data, outcome measurement and selective result reporting) and do not contribute directly to the assessment of recruitment bias; these were not evaluated within the current review since our focus was limited to recruitment bias.

Domain 1 includes two independent subdomains 1a and 1b. Each subdomain consists of a series of signalling questions, answers to which follow the algorithms provided (online supplemental material file 4, table S1) and ultimately lead to RoB judgement for subdomain 1a and subdomain 1b separately. RoB reporting includes a traffic-light system, where ‘low risk’ (green) shows minimal or no concerns regarding sources of bias, ‘some concerns’ (yellow/amber) indicates one or two moderate issues or several minor ones, and ‘high risk’ (red) signals major issues or the combination of more than two moderate ones. When assessing each signalling question, we responded ‘Yes’/‘No’ when clear information was provided, and ‘Possibly yes’/‘Possibly no’ when reported information is limited but implies a certain answer. In cases where no information was provided, we selected the option ‘No information’ (for detailed information on RoB algorithms refer to online supplemental material file 4, tables S1 and S2).

Both subdomains 1a and 1b are required for the assessment of recruitment bias. Subdomain 1a is an indicator of problems with the randomisation process. It includes assessment of whether randomisation was performed (signalling questions 1a.1), the allocation sequence was concealed (signalling question 1a.2), and if any baseline differences of individual participants between intervention groups resulted by chance (signalling question 1a.3). We judged sequence generation ‘random’ only when methods consistent with true randomisation were reported (eg, computer-generated sequences or an independent statistician’s algorithm). Where authors merely wrote that allocation was ‘random’ without method details, we coded 1a.1 as ‘no information’, automatically raising the RoB for Domain 1a to ‘some concerns’ by default. Allocation was considered concealed when the sequence was controlled by personnel independent to the enrolment process. Due to the nature of SW-CRTs, all clusters eventually reach the intervention condition; therefore, in cases when all clusters became aware of the allocation sequence at the beginning of the trial, we considered that the allocation sequence was not concealed (explicit statements on when the allocation sequence was revealed, otherwise we recorded ‘no information’). When assessing the baseline differences between intervention conditions, we used the following sequential approach. We used authors’ statements when these were explicitly provided. When authors’ statements were not provided, we examined baseline characteristics tables and narratively compared the reported summary statistics by the intervention conditions. Where p values for potential imbalances were reported, we noted them. We did not quantify differences with absolute standardised differences and did not compare differences between clusters in this subdomain.

Subdomain 1b is a marker for differential identification and recruitment of participants during screening and enrolment in clusters and is exclusive to cluster trials. It includes an assessment of whether the randomisation of clusters was performed prior to or after recruitment (signalling question 1b.1), and if so, were there any signs of differential recruitment (eg, recruiting staff and/or participants were aware of the allocation sequence which might have affected their decision to be included in a trial; signalling question 1b.2). To avoid any confusion, in this review, we used the term ‘selection bias’ only when referring to the results identified with signalling question 1b.2 (and indirectly with signalling question 1b.1, because in cases when recruitment occurred prior to randomisation, signalling question 1b.2 would be skipped). The term ‘differential identification and/or recruitment of individuals in clusters’ refers to the problems identified with signalling question 1b.2. Differential identification refers to the identification and screening process, and differential recruitment refers to the actual recruitment (often by research teams unmasked to allocation status). The subdomain 1b also includes assessment of intercluster baseline differences (signalling question 1b.3), including varying cluster sizes or imbalanced baseline characteristics between clusters that are not comparable with chance. When assessing whether all individual participants were identified and recruited before randomisation, we answered ‘Yes’/’Possibly yes’ when randomisation was performed after the recruitment process (eg, in closed cohorts); in other cases, particularly when recruitment was ongoing (eg, open cohort or cross-sectional design), the answer would be ‘No’/‘Possibly no’. Therefore, when individual recruitment followed cluster randomisation, we rated 1b as some concerns by default. We upgraded to high risk only when staff or participants were unmasked in ways plausibly able to influence who was approached, enrolled or analysed. However, in cases when masking was achieved and cluster characteristics were balanced, we downgraded 1b to low RoB. When assessing the possibility of differential recruitment, we focused on whether the recruiting personnel and potential participants were aware of the allocation sequence, and in cases when the allocation sequence was revealed in advance, we answered ‘Yes’/‘Possibly yes’ (depending on whether explicit information was provided). When direct mentioning of masking of the recruiting team was missing, but it was feasible to assess that the intervention condition was hidden at the site level, we coded this ‘Possibly no’ (eg, inclusion of all eligible participants, use of retrospective medical records, or concealed allocation sequence). Where masking was not reported and the intervention could not reasonably be hidden at the site level, we recorded ‘no information’ rather than inferring masking from the context. When assessing intercluster differences, we evaluated the imbalances in baseline characteristics between clusters (where reported) and the number of participants per cluster. Similarly to the assessment for signalling question 1a.3, we used author statements when these were explicitly provided. When authors’ statements were not provided, we examined baseline characteristics tables and narratively compared the reported summary statistics by clusters. Where p values for potential imbalances were reported, we noted them.

### Data management and analysis

Data extracted in Covidence were exported to Microsoft Excel for cleaning and summarising. Extracted categorical data were categorised into: (1) geographical region (based on World Bank classification (www.data.worldbank.org) (Europe, South Asia, East Asia and Pacific, Middle East, North America, Latin America and Caribbean, Africa, or multiple regions)); (2) population—based on participants’ age (over 18 years old—adults, under 18 years old—children and adolescents); (3) study setting —organisational units (eg, hospitals, schools, primary care facilities) which were not necessarily used as the unit of randomisation (eg, hospital wards within a hospital); (4) type of intervention (eg, educational, screening tool) based on how the authors of the original paper described their intervention. The summary statistics for these variables were presented as frequencies and percentages. The data were presented overall and by RoB category (high, some concerns, low). The differences between RoB groups were described narratively, without formal statistical testing due to a small number of studies per group.

RoB assessment results were presented in frequencies and percentages of studies with ‘high RoB’, ‘some concerns’ and ‘low RoB’ for subdomain 1a, 1b and domain 1 overall RoB. Since the objective of the review was to assess RoB in all eligible studies rather than pool the assessment results, no meta-analysis was performed. Extracted additionally to RoB assessment, type of randomisation and involvement of a trial member with statistical expertise who is independent to recruitment were reported as frequencies and percentages.

## Results

**Figure 1:**
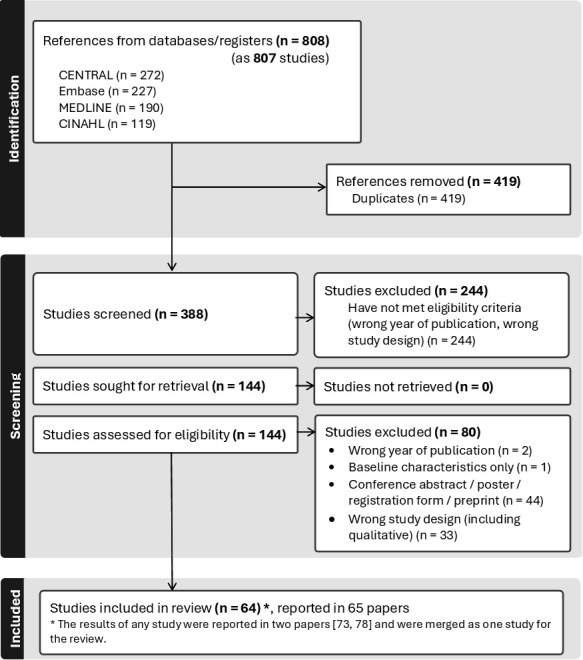
The flow chart of the search results (in accordance with the PRISMA guidelines^10^). PRISMA, Preferred Reporting Items for Systematic Reviews and Meta-Analyses.

Overall, 808 study records (from 807 trials) published in 2023 were identified. After removal of duplicates, 388 titles and abstracts were screened, 144 full texts were examined, and 64 trials (reported in 65 papers) were ultimately included in the final review^17^,^81^ (see figure 1). The results of one study were reported in two papers;^76 81^ the reported characteristics required for data extraction were identical, and the two papers were considered as one paper for the review. The results of Gyedu *et al* papers^33 34^ refer to one study but they report on different population groups (children and children plus adults); they were analysed as two separate studies. The results of six papers were published as preprints; the data were extracted from the preprint version and then retrospectively compared with 2024 versions—no discrepancies were identified.

During the full-text screening process, 80 studies were excluded due to the following reasons: 44 papers had limited study information available (conference abstracts or posters, registration forms); 33 papers had wrong study design (eg, before-and-after studies, difference in differences, qualitative analyses, systematic reviews); 2 papers were not published in 2023, and in 1 paper only the baseline characteristics were given, and recruitment and randomisation process was not reported.

### General characteristics of the included studies

Most of the studies were conducted either in North America (USA or Canada, 27 studies (42%)) or Europe (n=15; 23%) (see table 1). 53 studies (83%) recruited adults only, 7 (11%) recruited children and 4 (6%) recruited adults and children. 35 studies (55%) were conducted in hospitals or intensive care units (ICUs), and 12 studies (19%) were undertaken in primary healthcare facilities (note that the units of randomisation might be different from study settings). Other study settings included geographical areas and care homes and their analogues (four studies for each group), and three studies were conducted in other healthcare facilities; one study each was conducted in community gyms, homeless shelters, schools, office organisations and a special needs school. Around one-third of included studies (n=19, 30%) evaluated the effectiveness of educational programmes (eg, lifestyle modification advice or educational aids for healthcare professionals and patients), and in 13 studies (20%) interventions for disease prevention or early screening programmes were assessed. We planned to summarise data by journals, but it was not feasible as usually there was only one trial per journal. Further details of included studies are given in the online supplemental material file 5, table S1.

**Table 1:** General characteristics (geographical distribution, population groups, settings for cluster randomisation and type of intervention) in the included studies

Study characteristic	Number of studies (% of studies)			
	High risk of bias* (n=35)	Some concerns* (n=20)	Low risk of bias* (n=9)	Total† (n=64)
Region where the study conducted				
North America	11 (40.7)	11 (40.7)	5 (18.6)	27 (42.2)
Europe	10 (66.7)	4 (26.7)	1 (6.6)	15 (23.4)
Africa	7 (87.5)	–	1 (12.5)	8 (12.5)
East Asia and Pacific	5 (62.5)	3 (37.5)	–	8 (12.5)
South Asia	–	1 (100.0)	–	1 (1.6)
South America	1 (100.0)	–	–	1 (1.6)
Middle East	1 (100.0)	–	–	1 (1.6)
Multiple regions (worldwide)	–	1 (33.3)	2 (66.7)	3 (4.7)
Population				
Adults	30 (56.6)	16 (30.2)	7 (13.2)	53 (82.8)
Children	2 (28.6)	4 (57.1)	1 (14.3)	7 (10.9)
Adults and children	3 (75.0)	–	1 (25.0)	4 (6.3)
Study settings				
Hospitals	17 (62.9)	6 (22.2)	4 (14.9)	27 (42.2)
Primary healthcare facilities (eg, GP surgeries)	6 (50.0)	5 (41.7)	1 (8.3)	12 (18.8)
Intensive care units	6 (75.0)	1 (12.5)	1 (12.5)	8 (12.5)
Geographical areas	2 (50.0)	1 (25.0)	1 (25.0)	4 (6.4)
Care home and their analogues	–	4 (100.0)	–	4 (6.4)
Other healthcare facilities	1 (33.3)	1 (33.3)	1 (33.3)	3 (4.7)
Community gyms	1 (100.0)	–	–	1 (1.5)
Homeless shelters	1 (100.0)	–	–	1 (1.5)
Schools	–	1 (100)	–	1 (1.5)
Rehabilitation centres	1 (100.0)	–	–	1 (1.5)
Organisations (office workers)	–	–	1 (100.0)	1 (1.5)
Special needs school	–	1 (100.0)	–	1 (1.5)
Type of intervention				
Educational	11 (57.9)	5 (26.3)	3 (15.8)	19 (29.7)
Disease prevention/screening	8 (61.5)	4 (30.8)	1 (7.7)	13 (20.3)
Management tool	5 (41.7)	5 (41.7)	2 (16.6)	12 (18.8)
Cognitive behavioural therapy/behaviour intervention	2 (50.0)	2 (50.0)	–	4 (6.2)
Diagnostic	3 (75.0)	–	1 (25.0)	4 (6.2)
Pharmacological	3 (100.0)	–	–	3 (4.7)
Surgical	1 (100.0)	–	–	1 (1.6)
Other	2 (25.0)	4 (50.0)	2 (25.0)	8 (12.5)

Numbers and (percentages) are presented.

* The % was calculated per row (of all included studies in the subgroup).

† The % was calculated per column (of all included studies overall).

GP, general practitioner.

SW-CRTs conducted in Europe had a higher RoB (10/15, 67%) than in other regions, and trials conducted in hospitals, including ICUs, demonstrated a higher RoB (23/35, 66%) than in other settings. Educational trials also had a higher RoB (11/19, 58%) compared to other interventional trials.

### RoB assessment

Among 64 included studies, 35 (55%) were deemed as high RoB,^1821^,^24 26 28 29 33^ 20 (31%) as some concerns^2025 27 30^,^32 37 41 45 47 48 53 56 57 66 68 70 71 75 80^ and 9 (14%) as low risk^17 19 36 40 46 49 59 65 73^ (see table 2 for further information). In 12 studies (19%), RoB was deemed high in both subdomains 1a and 1b.^1824 29 42 43 54 61 67 76^,^79^ Similarly with general characteristics, further details of RoB assessment and justifications for all included studies are given in the online supplemental material file 5, table S1.

**Table 2:** Risk of bias assessment (with Cochrane RoB tool 2.0, extension for cluster trials^14^)

Risk of bias*	Number of studies (% of all included studies)†		
	Subdomain 1a	Subdomain 1b	Domain 1 overall‡
High risk of bias	24 (37.5)	23 (35.9)	**35** (**54.7**)
Some concerns	24 (37.5)	19 (29.7)	**20** (**31.3**)
Low risk of bias	16 (25.0)	22 (34.4)	**9** (**14.0**)

Numbers and (percentages) are presented. Numbers in bold represent the overall Domain 1 overall RoB.

* Subdomain 1a includes assessment of RoB arising from the randomisation process in a cluster-randomised trial. Subdomain 1b assessing the RoB arising from the identification or recruitment of participants into clusters.

† Domain 1 overall RoB is calculated based on the algorithms for Cochrane RoB tool 2.0: (1) if RoB is ‘low’ in subdomain 1a and subdomain 1b, domain 1 overall RoB was ‘low’, (2) if RoB in at least one subdomain is ‘some concerns’ and there is no ‘high’ RoB in any subdomain, domain 1 overall RoB was ‘some concerns’, (3) if RoB was ‘high’ in at least one subdomain, domain 1 overall RoB was ‘high’.

‡ Due to the RoB calculation algorithms described above, the number of studies for each RoB category in both subdomains and domain 1 overall is independent from each other; therefore, it is not expected that domain 1 overall number should be the sum of subdomain 1a and 1b results.

RoB, risk of bias.

**Figure 2:**
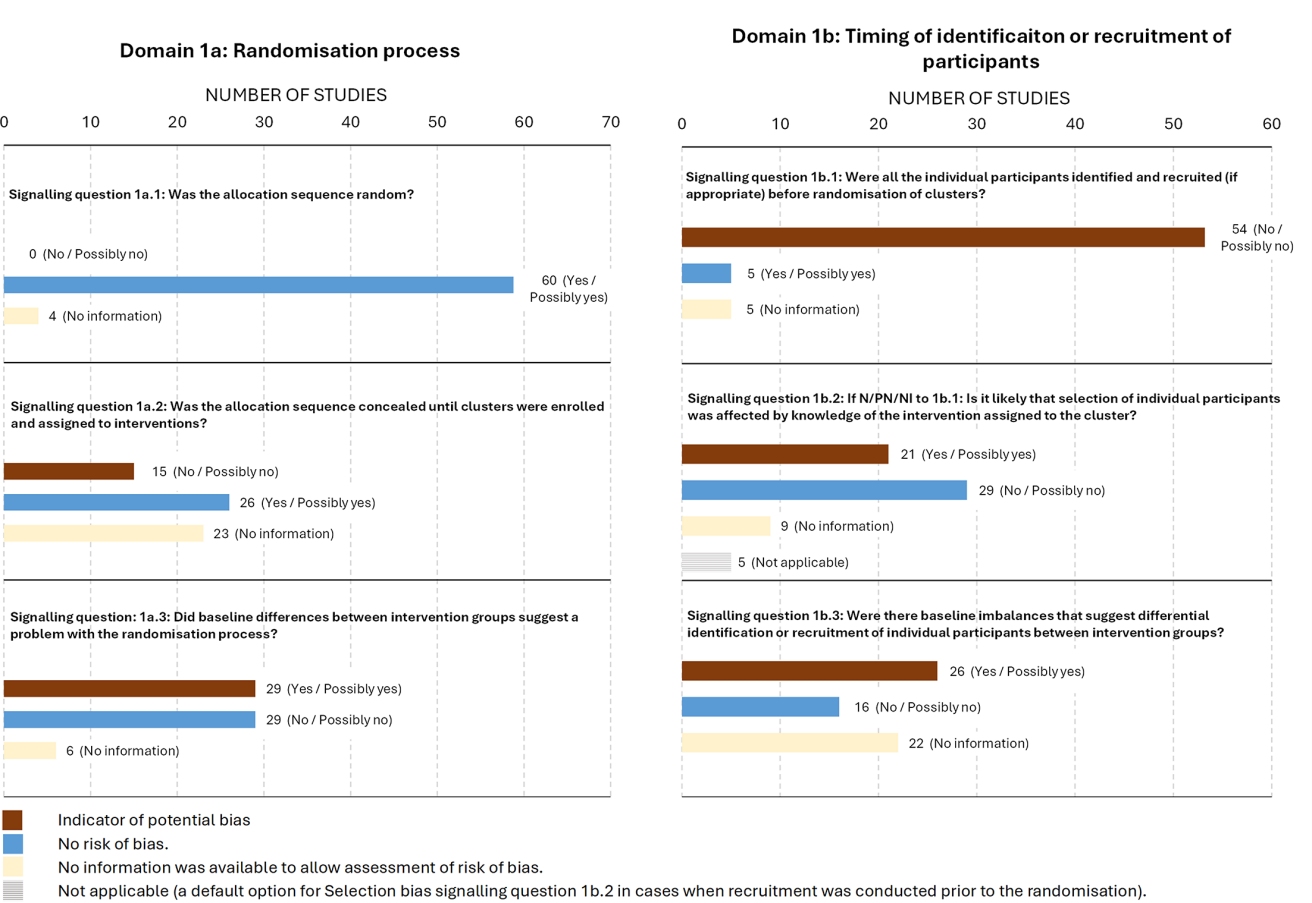
Responses to signalling questions in domains 1a and 1b.

Results of subdomain 1a revealed that 60/64 studies (94%) had clearly described the random allocation process whereas only 26/64 studies (41%) reported allocation concealment (see figure 2). The details of allocation concealment techniques in included trials are described in table 3. Among the trials where allocation concealment was not considered adequate (n=38), information was limited for RoB assessment in 23/38 studies (61%), and in 15 studies (15/38, 39%) the allocation sequence was revealed to all sites from the start (n=4),^21 39 42 79^ explicitly stated that it could not be masked (n=8),^24 54 62 63 67 69 77 78^ or the order was based on logistical (n=2)^44 50^ or geographical (n=1)^58^ factors and therefore could not be properly concealed.

**Table 3:** Mitigation strategies aiming to reduce the risks of unmasking in the included papers

Method	Masking achieved	Masking not achieved (potentially leading to higher risk of bias)
Allocation concealment (domain 1a, signalling question 1a.2)		
Central systems (eg, computer-generated sequence)	The sequence was controlled by personnel independent to the enrolment process (eg, as in refs. ^33 46^)	Recruitment and sequence generation were performed by the same person (as cannot be excluded in ref. ^39^)
Envelope methods	The use of opaque sealed envelopes (as reported in the trial) (eg, as in ref. ^52^)	No information regarding whether the envelopes were sealed, opaque or sequentially numbered (as in ref. ^72^)
Sequence foreknowledge	The sequence was revealed to the sites only before the intervention implementation (accounting for required training/transition period) (eg, as in refs. ^23 28 31^)	The sequence was revealed to all sites from the beginning (eg, as in refs. ^21 39 42 79^)
Selection bias (domain 1b, signalling question 1b.2)—only if identification and recruitment of all individual participants was not achieved before randomisation of clusters (1b.1)		
Inclusion of participants	Inclusion of all eligible participants based on *objective *inclusion criteria (this strategy still requires seeking participants’ informed consent) (eg, as in refs. ^32 36 69^)	Inclusion of all eligible participants based on *subjective* inclusion criteria (this strategy still requires seeking participants’ informed consent) (as in refs. ^23 24^)
	The use of waived consent (eg, as in refs. ^29 32 36^)	The use of individual informed consent from each participant/representative (eg, as in refs. ^61 72 76^)
Inclusion of medical records	Inclusion of (retrospective) medical records for all eligible patients (using such a strategy did not require informed consent to be sought (as in refs. ^30 38 40^)	

The type of randomisation was specified in 13 trials. Where reported, restricted randomisation was commonly used (n=11/64, 17%); simple (n=1/64, 1.5%) and matched-pair (n=1/64, 1.5%) were also reported. In one-third of all included studies (n=24/64, 38%), the authors mentioned a statistician was involved in the randomisation process.

Analysis of subdomain 1b revealed that the majority of the included studies recruited participants after randomisation (n=54/64; 84%). Among them, 56% of studies (n=30/54) were prone to selective recruitment of participants; these are studies where recruiting staff were not masked to the allocation status or no sufficient information was available to assess the question. However, in 29/64 (45%) trials, mitigation strategies were used to tackle potential selection bias in cases when identification and recruitment of all participants before randomisation was infeasible (table 3).

In 58/64 (91%) manuscripts, individual-level baseline characteristics by intervention conditions were presented; however, cluster-level data were given only in 42/64 (66%). Individual-level baseline differences were identified in 29/64 (45%) studies, while cluster-level baseline differences were identified in 26/64 (41%) studies. In 23/64 (36%) manuscripts, the authors adjusted statistical models to imbalanced covariates. Baseline characteristics per cluster were rarely reported (10/64, 16%). Shields *et al*^64^ and Krauss *et al*^42^ reported participant characteristics by clusters but not a comparison between intervention and control conditions; Yang *et al*^78^ and Leal *et al*^44^ reported characteristics separately by study step and allocation; and in four papers,^28 30 31 38^ data were reported by allocation and clusters. Inclusion of baseline characteristics in statistical analyses as covariates in adjusted regression models in either primary or sensitivity analyses was a method commonly used in included trials to mitigate potential baseline imbalances.^1823^,^26 30^ In a few cases, adjustments to models were not specified a priori: several authors^23 54 64^ acknowledged the need for post hoc adjustment for imbalanced baseline characteristics.

Importantly, imbalances in baseline characteristics do not necessarily indicate problems with randomisation or recruitment, and their presence alone might not necessarily lead to a high RoB. Among the trials with identified baseline imbalances in either subdomain 1a or 1b (n=41/64, 64%), 17 trials demonstrated signs of differential recruitment, but only in 7 of them did the authors associate imbalances with recruitment performed by an unmasked research team.^23 39 43 44 55 60 67^ In some trials (n=9), the authors acknowledged that recruitment was below the recruitment target, leading to underpowered results.^23 27 39 57 58 60 61 72 78^ In the majority of these cases (n=14 out of 20), imbalance problems were linked to variations in site demographics and processes^2831 34 47 50 51 61^,^63 68 72^ or site retention.^38 39 56^ In one paper,^26^ the rapid increase in the number of eligible participants was associated with the change of electronic health records provider. Another major reason for imbalances was the COVID-19 pandemic, causing early termination or halt of some trials,^18 24 32 33 42 57 74 75 77^ withdrawals of clusters^29 38^ and restructuring of wards,^18 38^ which affected the balance between allocation sequences and the comparability of clusters.

## Discussion

At the time of data extraction in 2023, PubMed recorded the highest number of published stepped-wedge trials (n=229) since the introduction of the stepped-wedge cluster design. This trend has continued, and in 2024, 299 papers referring to this design were published. In this review, 388 potentially relevant papers published in 2023 were screened, of which 64 met the inclusion criteria. Nearly half of these exhibited a high risk of recruitment bias.

In comparison to recent systematic reviews on different sources of bias in cluster RCTs, the RoB in this review appears to be slightly lower than in cluster trials. The review of forty randomly selected cluster RCT reports of individual-level interventions^82^ revealed that high RoB assessed with Cochrane RoB tool 2.0 arising from the randomisation process (subdomain 1a) was reported in over 50% of the included studies (vs 38% in this review). In that review, over 67% of studies were reported as ‘high risk’ of bias arising from the timing of identification and recruitment of individual participants (subdomain 1b) compared with 36% in this paper. Nevertheless, these figures are not directly comparable as the assessment of RoB remains a subjective process.^83^ In another recent review,^11^ 11 out of 23 studies (48%) were considered as ‘high RoB’, which is similar to the figure for SW-CRTs identified in our review (55%); however, the RoB assessment was not conducted with Cochrane RoB tool 2.0, and therefore, the direct comparison is likely to be less informative.

Generally, randomisation methods (signalling question 1a.1) were adequately reported. Only four articles^25 27 29 58^ lacked essential details of the randomisation process, such as details of how randomisation was actually performed. When reported, stratification was used for randomisation to ensure the better balance of baseline characteristics between cluster-level and individual-level characteristics. For some papers, however, the randomisation sequence was partly predetermined due to logistical issues based on some sites’ capacity^44 50^; therefore, facilities were informed of the allocation status even before the randomisation occurred. Additionally, some papers lacked details about the person responsible for the randomisation sequence. For example, the involvement of ‘an expert researcher’,^55^ ‘an independent consultant’,^22^ ‘a member of the research team’^64^ or even ‘the principal investigator’^76^ omits information whether this person had relevant experience in randomisation sequence generation. However, the involvement of an independent statistician could not guarantee the robustness of randomisation sequence generation, as in one trial,^78^ a statistician wrote cluster numbers on identical pieces of paper, placed them in blank envelopes, and representatives of each cluster drew lots to determine the allocation sequence. Although this method might provide unbiased results, it can be considered unconventional, particularly in comparison to the recommended centralised computer-generated sequence.

Allocation concealment (signalling question 1a.2) was poorly reported in 15/64 papers (23%) where no valuable information to address this element could be extracted. In one paper,^39^ the allocation sequence could not be concealed because the randomisation and recruitment were performed by one individual, although it is generally expected that enrolment is performed by an independent person. Moreover, in several trials with cross-sectional design (and therefore continuous recruitment), sites were informed of their allocated sequence before the enrolment,^54 63 67 79^ and unmasked recruiting staff were likely to contribute to differential identification and recruitment of potential participants. Shete *et al*^63^ reported that the sequence order was announced to all sites during the stakeholder-led randomisation ceremony with health centre representatives choosing a numbered ball with their sequence order from an opaque bag. Since the revealed allocation sequence with continuous recruitment design is likely to lead to potential selectivity when enrolling new participants, this paper had a high RoB. Additionally, the use of envelopes containing allocation information was reported in three papers.^52 72 80^ Although the use of sealed envelopes is more likely to lead to the subversion in allocation concealment than a centralised computer system,^84^ we considered the use of opaque sealed envelopes (as reported in the trial) as concealed allocation,^52^ while in one trial^72^ no information regarding whether the envelopes were sealed, opaque or sequentially numbered was given, leading to the higher RoB for this signalling question. Overall, allocation concealment in SW-CRTs is different from conventional cluster trials as recruitment of participants is often continuous but the clusters should become aware of transition order in advance. The current RoB tool does not allow for softening the RoB in such cases.

Due to the trial design, the recruitment of all participants prior to randomisation (signalling questions 1b.1) was challenging and often infeasible due to the sampling scheme. Only three trials used the closed-cohort design,^17 27 66^ and in all of them, all participants were enrolled during the recruitment stage and followed up longitudinally for the length of a study, whereas two of the trials^37 59^ did not recruit individual participants. 56 trials (56/64, 88%) had a cross-sectional or open-cohort design which meant participants were identified/recruited after randomisation. Therefore, the RoB could not be avoided due to the sampling strategy in these studies. Three papers were closed cohorts^51 64 73^; however, no explicit information on when randomisation was performed in relation to recruitment.

In cases when recruitment is ongoing and/or allocation sequence is revealed to all sites before the recruitment starts, it is crucial to mask recruiting staff to avoid selection bias (signalling question 1b.2). When the details of the recruitment process and staff masking were assessed, sometimes relevant information was missing or insufficient (n=9/59, 15%), or explicit statements were included that staff was unmasked (n=21/59, 36%). Schafthuizen *et al*^61^ also mentioned that referral to gatekeepers’ assistance for recruitment processes may also violate the unbiased approach to treatment, especially when gatekeepers are aware of the sequence allocation and recruitment is continuous.

In order to minimise any potential selectivity while recruiting continuously in open cohort and cross-sectional designs (signalling question 1b.2), the authors used different mitigation strategies by including all eligible participants, using a consent waiver, or using the medical records of all eligible patients. These strategies allow the inclusion of all or almost all patients, therefore minimising the role of selectivity in recruitment. It is worth noting that the use of a waived informed consent could be an effective mitigation strategy only if inclusion criteria for participation are based on objective measures, for example, age,^3236 44^,^47 53 69^ diagnosis,^46 50 69^ laboratory values^46^ or number of visits.^36 69^ However, where a subjective measure is used, such as expected length of stay in healthcare facility (generally based on clinical judgement rather than objective tests),^23 24^ the use of a waived informed consent cannot eliminate the risk of differential recruitment.

Imbalanced baseline characteristics might be used as an indicator of recruitment bias. The review by Bolzern *et al*^11^ found that the risk of recruitment bias was much higher for cluster trials compared with individually randomised trials, particularly in terms of baseline characteristics such as age. The authors compared age imbalances in cluster and individual RCTs in meta-analyses and revealed that while in all individually randomised trials age was balanced between intervention groups, in 14 out of 23 cluster trials (61%) age imbalances were identified. In our review, a number of the included studies had individual-level covariate imbalance in baseline characteristics (n=29, 45%) particularly in clinically important variables (eg, number of comorbidities and severity of disease,^18 29 31 55 58^ number of hospital admissions or length of stay in hospital^29 55 78^). Generally, the number of participants per cluster was well-reported; however, the assessment of intercluster differences in participant characteristics was problematic as only a few papers (n=10, 16%) included detailed data by cluster. Importantly, these imbalances can be symptomatic of other issues such as small cluster numbers, time-varying site characteristics or inadequate randomisation methods. An additional challenge was the interpretation of a CONSORT flow diagram for SW-CRTs; in particular, it was more complicated to assess the participants’ flow in sequences or clusters when the results were displayed on a CONSORT flow diagram for individually randomised trials, allowing comparison in the number of participants between intervention and control conditions only.^29 57 77^ Rarely, baseline characteristics of clusters were reported with any disaggregation.^51 73^

The imbalances on an individual level were noted in 29/64 trials (45%) and on a cluster level in 26/64 (41%) in this review comparing to ‘traditional’ cluster RCTs with 75% and 38%, respectively.^82^ Although some of these imbalances might have been due to chance, the risk of recruitment bias could not be ruled out completely. Importantly, regardless of the study design (cross-sectional or open cohort), time-related (eg, seasonal) imbalances in site-level covariates might be expected.^85^ Some of these time-related factors are usually uncontrollable and/or unavoidable (eg, general policy changes, staff turnover, seasonal changes such as different hospital load in winter and summer, different infection rates throughout the year^47 86^), whereas other factors (eg, staff rotation or recruitment performed by unmasked team members) can be avoided. Hypothetically, in cases when the potential risk of any time-related changes on site level required mitigation actions, implementation of fewer cluster sequences with greater number of clusters per sequence might be more effective because trial duration (and consequently the risk of any uncontrollable changes) will be minimised. However, it might cause logistical and financial problems. Time constraints might also negatively affect outcome measures, as for some interventions their effect could be delayed. In the paper by Irvine *et al*,^37^ the authors restricted the enrolment window for each implementation period, permitting more outcome observation time for each participant before the next step and limiting the risk of time-related confounding. This was a pragmatic strategy and an example of good practice.

### Limitations

This review has several limitations. The search strategy was limited to only 1 year (2023), and although expected to demonstrate the most current practices in addressing the risk of recruitment bias, it is also likely to violate generalisability of the review results. We acknowledge that recency might improve reporting; however, expanding the timeframe would have required additional resources that were not at the team’s disposal. Therefore, as a trade-off, we decided to limit the included timeframe to 2023 but analyse the papers in detail. Importantly, the choice of 2023 implied taking into consideration direct effects or delayed consequences of the recent COVID-19, which might have affected the recruitment process^87^; however, in this review, no adjustments for the recent pandemic were made. Also, although international electronic databases were screened, the predominant language of the screened publications was English.

The RoB assessment was performed with the Cochrane RoB tool for cluster RCTs, and only one domain of the RoB assessment tool was used (out of five); therefore, current domain 1 overall RoB does not necessarily match the actual RoB for the study measured with all five domains; however, ‘high’ RoB in at least one domain automatically raises overall RoB (assessed with all five domains) to the maximum level. Importantly, since the majority of included studies had a cross-sectional design, it would be problematic to extract data regarding lost to follow-up rates, which could be an additional marker of recruitment bias.^88^ Also, the RoB assessment remains a subjective process. When p values were reported in baseline tables, we used them to assess signalling questions 1a.3 and 1b.3 alongside the authors’ comments, acknowledging that using p values is not recommended for differences assessment.

### Recommendations

This review identified various sources of bias in SW-CRTs. Although some problems were detected with the subdomain 1b signalling questions, these were designed to assess RoB in ‘conventional’ cluster RCTs; some of these problems appeared to be unavoidable in stepped-wedge cluster trials due to sampling schemes used. Whereas identification and recruitment of all participants before the randomisation of clusters is usually expected in cluster RCTs, this might be less feasible in SW-CRTs due to the time factor, particularly for SW-CRTs with a cross-sectional design and, consequently, ongoing recruitment. Masking of the recruiting research staff could be an effective mitigation strategy to reduce the risk of potential selection bias, but its applicability might be limited in SW-CRTs when the allocation sequence is required to be known in advance so the intervention implementation could be planned while in a control condition. Statistical analysis adjusting for imbalanced baseline characteristics (either a priori or post hoc) can still be useful to minimise the RoB influence on the trial results.

At the same time, the role of rigorous, transparent and detailed reporting remains essential. The quality of reporting of randomisation process, allocation sequence masking, recruitment processes and blinding of research staff was poor in some trials, consequently leading to the higher RoB. Authors should be encouraged to strictly follow the reporting recommendations, including CONSORT extension to SW-CRT guidelines on detailed reporting of randomisation process and allocation concealment.^2 89^ Type of randomisation (eg, simple, restricted) must be reported in all trials. The allocation sequence is expected to be prepared by independent personnel not involved in the recruiting process, and such sequence must remain concealed until the allocated transition period occurs. Although CONSORT guidelines do not particularly emphasise reporting the masking status of recruiting staff, it is still expected that masking status of all research team members is disclosed. Since lack of details and underreporting often lead to a higher RoB assessment, transparent and detailed reporting of trials, randomisation and recruitment process is essential for reducing the risk of recruitment bias.

However, SW-CRTs have important differences from ‘conventional’ cluster trials. Not only do all clusters in SW-CRTs receive the intervention, but the recruitment process often cannot be completed before randomisation occurs, particularly in trials with cross-sectional design (eg, in intensive care wards), where continuous follow-up of the same participants is not possible due to high patient turnover. Also, the concealment of the allocation sequence up to the start of the intervention period is likely to be impractical because, depending on the intervention, preparation to the intervention roll-out might require significant amounts of time. Still, masking of recruiting staff seems to be achievable, although it might be facing challenges when the cluster intervention status cannot be masked. All these peculiarities of SW-CRT lead to the necessity of the development and validation of a RoB tool separate for SW-CRTs, as the current tool does not correctly address the issues of this design. However, the RoB tool for crossover trials might be valuable in the development of the separate tool for SW-CRTs, because SW-CRTs in nature are crossover trials, but this crossover happens always from the control to the intervention phase.

Theoretically, a combination of the RoB tool for cluster trials^14^ with the RoB tool for crossover trials^90^ would address some design challenges of SW-CRTs overlooked by the cluster RoB tool, including adjusting for a trial period. The RoB tool for crossover trials includes three signalling questions in the special Domain S: confirmation of the equal number of participants per sequence (signalling question S1), inclusion of period effects in the analysis in cases where there is an imbalance between sequences (signalling question S2), and appropriate duration of a transition period to allow any carryover effects to disappear (signalling question S3). The suggested combination of these tools is given in table 4.

**Table 4:** Possible adaptation of the existing RoB tools to create a separate SW-CRT tool

Domain	Signalling question based on existing RoB tools	Comments to adapt signalling questions to create a separate RoB tool for SW-CRT
Domain 1a	Randomised controlled trial element	
	1—Randomisation occurred (signalling question 1a.1 from cluster RCT tool)	As in parallel trials (ie, explicit statement of what method of randomisation has been used)
	2—Allocation concealed (adapted signalling question 1a.2 from cluster RCT tool)	As in parallel trials, the allocation sequence should not be revealed before the intervention step accounting for preparation time and transition period (logistical issues). For trials with continuous recruitment or cross-sectional/open cohort design, mitigation strategies should be considered, particularly when the recruiting staff cannot be masked to the allocation status.
	3—Baseline characteristics balanced (on individual participant level) (signalling question 1a.3 from cluster RCT tool)	As in parallel trials. Formal statistical testing of differences in individual baseline characteristics between intervention and control conditions is not required and not expected
Domain 1b	Cluster element	
	1—Recruitment before randomisation (adapted signalling question 1b.1 from cluster RCT tool)	The question applies only for closed cohort design, for open cohort and cross-sectional design this question is not required
	2—Masking of recruiting staff and patients (signalling question 1b.2 from cluster RCT tool)	The question should be skipped if recruitment occurred *before* randomisation (as in cluster trials)
	3—Baseline sequence characterises balanced, including the number of participants per sequence (adapted signalling question S1 from crossover RCT tool)	In SW-CRTs, the unit of randomisation is clusters within sequence, therefore it might be more valuable to compare the differences between sequences. The data should be presented per cluster/sequence (as appropriate).
Domain 1c	Time element	
	1—Were period effects accounted for in the analysis? (adapted signalling question S2 from crossover RCT tool)	In the RoB tool for crossover trials, this signalling question is required only if the number of participants in the sequences was not balanced, and such, any period effects identified may lead to biased results. For SW-CRTs, temporal effects are also crucial to consider for the analysis. This is particularly due to generally longer trial duration with potential secular trends and regular repeated outcome collection. Therefore, for SW-CRTs, the period effect should be adjusted for in the analysis models.

RCT, randomised controlled trial; RoB, risk of bias; SW-CRT, stepped-wedge cluster randomised trial.

Such a tool would still demand high quality and transparency when reporting the details of the randomisation process (which is also in line with CONSORT guidelines). The role of recruitment before randomisation would be less significant; however, the role of masking of research staff involved in recruitment would still be essential. Also, the baseline comparison between sequences (rather than between clusters) would be more reasonable as more than one cluster can be part of a sequence. When only one cluster is randomised per sequence, the baseline comparison between sequences will be equivalent to the comparison between clusters. Furthermore, temporal effects might be crucial in SW-CRTs to account for any trends over time (either external (eg, seasonal changes) or internal (eg, more active recruitment in the intervention phase)). Where possible, a transition period should be incorporated into the trial design to allow for a smooth implementation of the intervention in the next step of the sequence.

Further methodological research is required to investigate the adherence of SW-CRTs to CONSORT guidelines. The development of a new tool for RoB assessment of SW-CRTs is also highly desirable. The role of ‘No information’ response to signalling questions should be reviewed as currently it often does not aggravate the potential RoB.

## Conclusions

As the popularity of the SW-CRT continues to increase exponentially, it becomes crucial to ensure that such studies are rigorous and methodologically sound. While the results of SW-CRTs are widely being used for further healthcare policies implementations and updates, the RoB, particularly when avoidable, must be minimised.

## Supplementary material

10.1136/bmjopen-2024-096281 online supplemental file 1

10.1136/bmjopen-2024-096281 online supplemental file 2

10.1136/bmjopen-2024-096281 online supplemental file 3

10.1136/bmjopen-2024-096281 online supplemental file 4

10.1136/bmjopen-2024-096281 online supplemental file 5

## Data Availability

All data relevant to the study are included in the article or uploaded as supplementary information.
