# Supplementary material for: Role of recruitment bias in stepped-wedge cluster randomised controlled trials: a systematic review
Source: BMJ Open. 2025 Nov 28;15(11):e096281. doi: 10.1136/bmjopen-2024-096281 (PMC12666225; doi:10.1136/bmjopen-2024-096281)
Supplement: online supplemental file 2 [file bmjopen-15-11-s002.pdf]

Supplementary Material File 2: Search strategies

Searches conducted on 09/02/2024.

1. **MEDLINE (Ovid)** - 190 publications identified

1	stepped wedge.mp.
2	stepped-wedge.mp.
3	1 or 2
4	random*.mp.
5	randomised controlled trial.mp. or randomized controlled trial or exp Randomized Controlled Trials as Topic/
6	rct.mp.
7	4 or 5 or 6
8	cluster*.mp.
9	3 and 7 and 8
10	study protocol.ti.
11	9 not 10
10	limit 11 to yr="2023"

2. **EMBASE (Ovid)** - 227 publications identified

1	stepped wedge.mp.
2	stepped-wedge.mp.
3	1 or 2
4	random*.mp.
5	randomised controlled trial.mp. or randomized controlled trial or exp Randomized Controlled Trials as Topic/
6	rct.mp.
7	4 or 5 or 6
8	cluster*.mp.
9	3 and 7 and 8
10	study protocol.ti.
11	9 not 10
10	limit 11 to yr="2023"

3. **Cochrane Library (Willey)** - 272 publications identified

1	stepped wedge
2	stepped-wedge
3	cluster*
4	MeSH descriptor: [Randomized Controlled Trials as Topic] explode all trees
5	random*
6	#1 or #2
7	#4 or #5
8	#3 and #6 and #7 (with Publication Year from 2023 to 2023, with Cochrane Library publication date from Jan 2023 to Dec 2023, in Trials)

4. **CINAHL Ultimate (EBSCO) -** 119 publications identified

stepped wedge OR stepped wedge design OR stepped-wedge AND (randomized controlled trials or rtc or randomised control trials) AND cluster

Publication Date: 20230101-20231231
