# Supplementary material for: Role of recruitment bias in stepped-wedge cluster randomised controlled trials: a systematic review
Source: BMJ Open. 2025 Nov 28;15(11):e096281. doi: 10.1136/bmjopen-2024-096281 (PMC12666225; doi:10.1136/bmjopen-2024-096281)
Supplement: online supplemental file 3 [file bmjopen-15-11-s003.pdf]

Supplementary Material File 3: Screening and extraction process

Table S1. Eligibility criteria for included studies

Inclusion criteria	Exclusion criteria
Population: any<br>Intervention: any<br>Comparator: any<br>Outcome: any<br>Study design: Stepped-wedge cluster randomised controlled trial<br>Year of publication: 2023<br>Language: any<br>Settings: any	Animal studies<br>Feasibility studies<br>Pilot studies<br>Ongoing studies / study protocols<br>Any study design other than SW-CRT (including observational studies, non-randomised controlled trials, qualitative studies, before-and-after studies)

Table S2. Data extraction table

<table>
<tr><th>Study ID (Author, Year, Title)</th><th>Country (Region)</th><th>Age group</th><th>Settings</th><th colspan="2">Intervention (description)</th><th>Updated CONSORT diagram?</th></tr>
<tr><td></td><td></td><td></td><td></td><td colspan="2"></td><td></td></tr>
<tr><th colspan="4">Signalling question / Response (Y/PY/NI/PN/N)</th><th>Justification</th><th>Domain RoB</th><th>Overall RoB</th></tr>
<tr><td colspan="4">1a: Allocation sequence random?</td><td></td><td rowspan="3">Low /
Some concerns /
High</td><td rowspan="6">Low /
Some concerns /
High</td></tr>
<tr><td colspan="4">1b: Allocation sequence concealed?</td><td></td></tr>
<tr><td colspan="4">1c: Any baseline differences?</td><td></td></tr>
<tr><td colspan="4">2a: Recruitment prior to randomisation?</td><td></td><td rowspan="3">Low /
Some concerns /
High</td></tr>
<tr><td colspan="4">2b: Signs of selection bias?</td><td></td></tr>
<tr><td colspan="4">2c: Cluster baseline imbalance?</td><td></td></tr>
</table>
