# Supplementary material for: Role of recruitment bias in stepped-wedge cluster randomised controlled trials: a systematic review
Source: BMJ Open. 2025 Nov 28;15(11):e096281. doi: 10.1136/bmjopen-2024-096281 (PMC12666225; doi:10.1136/bmjopen-2024-096281)
Supplement: online supplemental file 4 [file bmjopen-15-11-s004.pdf]

Supplementary Material File 4: Risk of bias assessment

Table S1. Scoring algorithms for risk of bias assessment (in accordance with Cochrane Risk of Bias Assessment Tool 2.0 for cluster randomised trials [1]). Figure 1 (above) illustrates the scoring process within Domain 1a, and Figure 2 (below) illustrates the scoring process for Domain 1b.

Figure 1. Algorithm for suggested judgement of risk of bias judgements for bias arising from the randomization process in a cluster-randomized trial

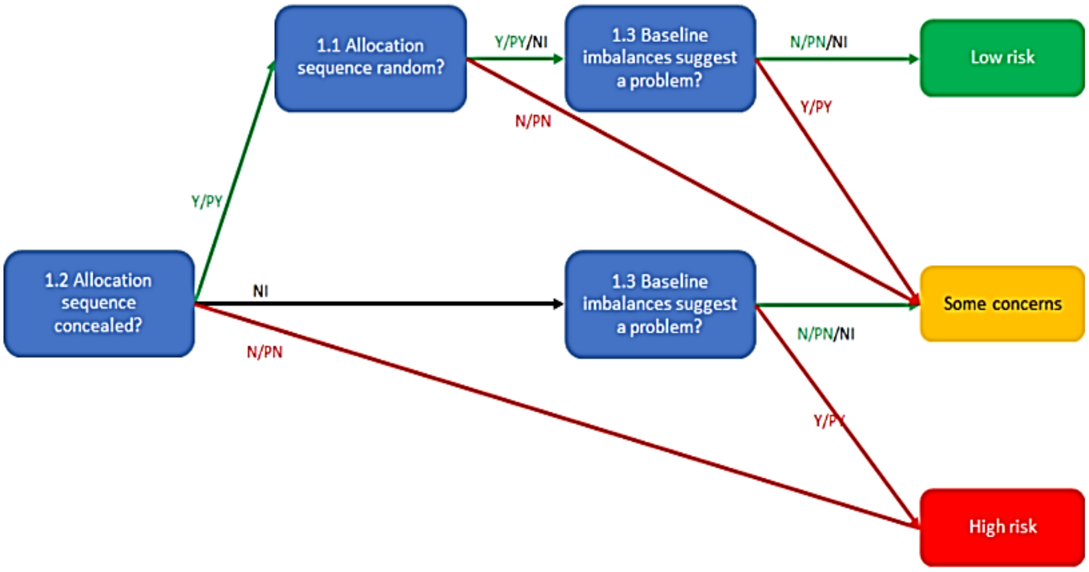

Figure 2. Algorithm for suggested judgement of risk of bias judgements for bias arising from the timing of identification and recruitment of participants in a cluster-randomized trial

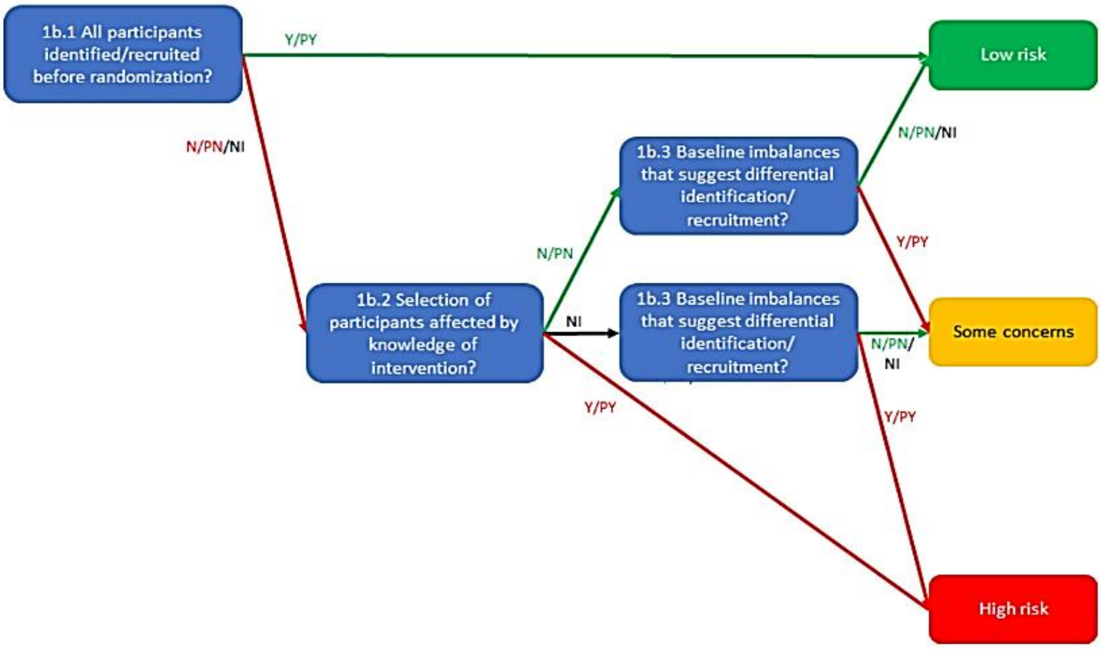

Table S2. Overall risk of bias judgement (in accordance with Cochrane Risk of Bias Assessment Tool 2.0 for cluster randomised trials [1]). The overall risk of bias judgement is being performed when all domains are being analysed.

**Overall risk-of-bias judgement**	**Criteria**
Low risk of bias	The study is judged to be at **low risk of bias for all domains** for this result.
Some concerns	The study is judged to raise **some concerns in at least one domain** for this result, but **not to be at high risk of bias for any domain**.
High risk of bias	The study is judged to be at **high risk of bias in at least one domain** for this result.<br>OR<br>The study is judged to have **some concerns for multiple domains in a way that substantially lowers confidence** in the result.

References:

1. Eldridge, S. et al. (2021). Revised Cochrane risk of bias tool for randomized trials (RoB 2.0): additional considerations for cluster-randomized trials. Cochrane Methods. Cochrane Database Syst Rev, 10. [Online]. Available at: https://drive.google.com/file/d/1yDQtDkrp68_8kJilUdbongK99sx7RFI-/view.
