# Supplementary material for: Role of recruitment bias in stepped-wedge cluster randomised controlled trials: a systematic review
Source: BMJ Open. 2025 Nov 28;15(11):e096281. doi: 10.1136/bmjopen-2024-096281 (PMC12666225; doi:10.1136/bmjopen-2024-096281)
Supplement: online supplemental file 5 [file bmjopen-15-11-s005.pdf]

Supplementary Material File 5: Description of all included studies

Table S1. Description of all included studies

Study ID	Country (Region)	Age group	Settings	Intervention (description)
Aegerter et al., 2023	Switzerland (Europe)	Adults	Organisations (office workers)	Educational (the intervention consisted of a workstation ergonomics intervention, weekly group health-promotion information workshops, and neck exercises)

Domain		Signalling question	Response	RoB Justification	Domain	Overall
Domain	1a	1a: Allocation sequence random?	PY	*"These 15 groups were then randomly assigned to the intervention cluster (1, 2, and 3) by computer by a senior biostatistician (TV) who was blinded to the identity of the participants". Regarding concealment of the allocation sequence (from the protocol) – "The study coordinator will notify individuals of their allocation, collect baseline data, and communicate between participants and the intervention health professional to organize assessments".*	Low	Low
	1a	1b: Allocation sequence concealed?	PY			
	1a	1c: Any baseline differences?	NI	In the paper, no data comparing characteristics in intervention and control group were given (neither between nor within clusters). Baseline table comprise the characteristics for the study population in total.		
	1b	2a: Recruitment prior to randomisation?	Y	*"Participants who worked in the same organisation, on the same floor or in the same room were assigned to the same group (de-identified by AA) to avoid contamination, resulting in a total of 15 groups of 8 participants in each. These 15 groups were then randomly assigned to the intervention cluster (1, 2, and 3) by computer by a senior biostatistician (TV) who was blinded to the identity of the participants".*	Low	
	1b	2b: Signs of selection bias?	NA	Since all participants were enrolled before the randomisation of clusters, there was no risk of selection bias (not being assessed according to Cochrane RoB tool 2.0).		
	1b	2c: Cluster baseline imbalance?	PN	No precise data is given in Table 1, but imbalances appear to result from a random error and highly unlikely due to the rigorous recruitment process.		

Study ID	Country (Region)	Age group	Settings	Intervention (description)
Åhsberg et al., 2023	Ghana (Africa)	Adults	Hospitals	Disease prevention / screening (provision of Determine LAM (lipoarabinomannan assay) tests to enable point-of-care (POC) tuberculosis (TB) diagnosis in addition to routine care.)

Domain		Signalling question	Response	RoB Justification	Domain	Overall
Domain	1a	1a: Allocation sequence random?	PY	*"Cluster randomization was undertaken by an impartial biostatistician at the Open Patient Data Explorative Network, Odense University Hospital, with open-labeled allocation. On each switch from control to intervention, the study site was provided with Determine LAM tests to enable POC TB diagnosis".*	High	High
	1a	1b: Allocation sequence concealed?	NI			
	1a	1c: Any baseline differences?	PY	*"Baseline characteristics also differed between study groups with higher rates of comorbidities, older age, lower female-male ratio, and higher body mass index in the intervention group (Table 1)".* The number of patients in in the Intervention group was 174, and 248 were in Control group. "Due to the COVID-19 pandemic, the sample size was not reached".		
	1b	2a: Recruitment prior to randomisation?	PN	*"Patients were consecutively enrolled within a maximum of 72 hours from patient admission at the current ward".* Therefore, the recruitment of all participants before the cluster randomisation was impossible.	High	
	1b	2b: Signs of selection bias?	NI	No information if the recruiting staff was blinded to the intervention allocation		
	1b	2c: Cluster baseline imbalance?	PY	Between clusters, there were imbalances in female-male ratio, availability of HIV RNA results, CD4 counts, WHO danger sign distribution, and comorbidities rates. (Table S3, Supplementary material). The ratio of participants in Intervention and Control groups differed noticeably between the clusters (hospitals), with similar numbers for Korle Bu teaching Hospital (75 participants in Intervention and 67 participants in Control), somewhat higher number of participants in Intervention group in Lekma Hospital (58 participants in Intervention and 33 in Control), and dramatically huge difference for Tema General Hospital (41 in Intervention and 148 in Control). "Due to the COVID-19 pandemic, the sample size was not reached".		

Table S1 (continues)

Study ID	Country (Region)	Age group	Settings	Intervention (description)
Ambasta et al., 2023	Canada (North America)	Adults	Hospitals (laboratory tests of clinic patients)	Diagnostic (intervention bundle to reduce repetitive use of routine laboratory testing in hospitalised patients)

Domain		Signalling question	Response	RoB Justification	Domain	Overall
Domain	1a	1a: Allocation sequence random?	Y	*"The order each site entered the intervention period was randomly selected using a random number generator. All four sites had agreed to their involvement before they were randomised, and none dropped out after randomisation".*	Low	Low
		1b: Allocation sequence concealed?	Y			
		1c: Any baseline differences?	PN	Although baseline data (between and within clusters) for intervention and control groups is given in Table 1, there is no information regarding the statistically significant difference between groups provided. Imbalances, however, appear to result from a random error.		
	1b	2a: Recruitment prior to randomisation?	PN	Infeasible in terms of intervention (ongoing "recruitment" – analysis of all medical tests).	Low	
		2b: Signs of selection bias?	PN	Highly unlikely in terms of intervention (no inclusion and exclusion criteria for patients).		
		2c: Cluster baseline imbalance?	PN	No precise data is given in Table 1, but imbalances appear to result from a random error.		

Study ID	Country (Region)	Age group	Settings	Intervention (description)
Anastario et al., 2023	United States (North America)	Children	Schools (voluntary pupils' recruitment)	Disease prevention / screening (intervention to prevent teen pregnancy)

Domain		Signalling question	Response	RoB Justification	Domain	Overall
Domain	1a	1a: Allocation sequence random?	PY	*"Schools were randomized to the intervention using a single sequence random assignment to clusters that determined the order of intervention receipt. The study's analyst randomized clusters to N/E following the first round of baseline data collection".*	Some concerns	Some concerns
		1b: Allocation sequence concealed?	NI			
		1c: Any baseline differences?	PN	There is no information about baseline differences between Intervention and Control group (within or between clusters). *"There were no appreciable differences in endorsement of COVID-19-related items between the 96 participants who had completed the intervention, and the 22 participants who did not complete the intervention (see Table 2)".*		
	1b	2a: Recruitment prior to randomisation?	PN	Although the study design was an open cohort, the study does not provide any information about the possible ongoing recruitment.	Low	
		2b: Signs of selection bias?	PN	As the recruitment was completed before the randomisation, and eventually all clusters (schools) have received the intervention, it seems to be unlikely.		
		2c: Cluster baseline imbalance?	PY	The number of students in the Intervention group was 96, and in Control group was 22. No data allowing comparison between clusters are available.		

Table S1 (continues)

Study ID	Country (Region)	Age group	Settings	Intervention (description)			
Berdal et al., 2023	Norway (Europe)	Adults	Rehabilitations centres (clinic patients)	Educational (the BRIDGE-intervention was directed at improving the routines and communication skills of health professionals in secondary healthcare as regard goal setting, action planning, and psychological support, and at engaging and motivating patients to take an active role in pursuing their rehabilitation goals over time).			
		Signalling question / Response		RoB Justification		Domain	Overall
Domain	1a	1a: Allocation sequence random?	Y	*"The cluster-level randomization was performed using a computer-generated list of random numbers. The eight clusters were given a number between 1 and 8 before they were randomly allocated to one of eight sequences. The sequential intervention rollout plan was presented to the clusters at the study start, and accordingly, the intervention providers were not blinded to group allocation.".*		High	High
		1b: Allocation sequence concealed?	N				
		1c: Any baseline differences?	PY	*"The treatment groups were mostly well-balanced with respect to baseline characteristics, however, some differences in the distribution of diagnoses were seen. Further, the patients in the intervention group were significantly younger, with shorter disease duration compared to the control group".* No cluster level data was provided to allow comparisons.			
	1b	2a: Recruitment prior to randomisation?	N	*"For patients, group allocation was determined by their admission dates, and whether the pertaining clusters were in the control- or intervention phase on that date".* Recruitment was ongoing, identification of all participants before the cluster randomisation was impossible.		Some concerns	
		2b: Signs of selection bias?	PN	*"All patients were given identical study information on admission before consenting to participate, but to avoid unblinding disparate verbal information was given, by leaving out the particulars of the experimental intervention to potential participants in the control group". Patients were screened for eligibility and recruited on admission to rehabilitation stay by local project coordinators at each participating center.* No information available whether the recruiting staff was blinded to the cluster allocation status; however, wide eligibility criteria are likely to minimise any risk of selection bias.			
		2c: Cluster baseline imbalance?	NI	*"The treatment groups were mostly well-balanced with respect to baseline characteristics, however, some differences in the distribution of diagnoses were seen. Further, the patients in the intervention group were significantly younger, with shorter disease duration compared to the control group".* No cluster level data was provided to allow comparisons.			

Study ID	Country (Region)	Age group	Settings	Intervention (description)			
Blackberry et al., 2023	Australia (East Asia and Pacific)	Adults	Geographical areas (rural communities)	Educational (This study investigated the effect of a Virtual Dementia-Friendly Rural Communities (Verily Connect) model on social support and demand for caregivers of people living with dementia)			
		Signalling question / Response		RoB Justification		Domain	Overall
Domain	1a	1a: Allocation sequence random?	Y	*"An independent consultant used stratified randomization to produce the schedule".*		Some concerns	High
		1b: Allocation sequence concealed?	NI				
		1c: Any baseline differences?	NI	No baseline data for Intervention and Control groups separately are presented (only demographic characteristics for all caregivers) (Table 1). The equal number of clusters randomised to Intervention and Control groups (Figure 3).			
	1b	2a: Recruitment prior to randomisation?	N	The study was an open cohort. "*… there were additional enrolments with each implementation step, comprising participants from communities that were moving from the control to the implementation phase…"*		High	
		2b: Signs of selection bias?	PY	*"Although cluster sampling was randomised, the recruitment of individual participants involved convenience sampling. Although the Verily Connect website and app were available on public platforms, the study project management team had control over who could sign up and access the app, thus restricting access to only enrolled participants".* No information is provided if the recruiting staff was blinded to the allocation status while recruiting.			
		2c: Cluster baseline imbalance?	NI	No baseline data for Intervention and Control groups separately are presented (only demographic characteristics for all caregivers) (Table 1).			

Table S1 (continues)

Study ID	Country (Region)	Age group	Settings	Intervention (description)		
Bollaert et al., 2023	France (Europe)	Adults	Intensive care unit (clinic patients)	Pharmacological (assess the effectiveness of a fluid balance control strategy on mortality in critically ill patients)		
	Signalling question / Response		RoB Justification		Domain	Overall
Domain 1a	1a: Allocation sequence random?	Y	*"JMV used computer-generated numbers to randomly assign each ICU to one of the 12 sequences".*		Some concerns	High
Domain 1a	1b: Allocation sequence concealed?	Y	*"ICU staff were informed about their own allocation 1 month before their time of inclusion. ICUs were blinded to other ICUs allocation (until allocation occurrence). Once an ICU was assigned to a sequence, the research team met with the whole ICU staff and the dedicated onsite research nurse to inform them about the trial procedures for the control period during the month before the time of inclusion".*			
Domain 1a	1c: Any baseline differences?	PY	*"As compared with the control group, the strategy group was younger and had lower SAPS II and poorer McCabe score. They also had lower potassium level and higher bicarbonates level. In addition, they were more often admitted to the ICU due to central nervous system (CNS) injury than to any other causes".* No baseline characteristics between clusters are given.			
Domain 1b	2a: Recruitment prior to randomisation?	N	Due to the nature of the intervention and settings (ICU), it was not feasible.		High	
Domain 1b	2b: Signs of selection bias?	PY	Recruitment team was not blinded to the allocation status during the recruitment stage since *"ICU staff were informed about their own allocation 1 month before their time of inclusion. ICUs were blinded to other ICUs allocation (until allocation occurrence). Once an ICU was assigned to a sequence, the research team met with the whole ICU staff and the dedicated onsite research nurse to inform them about the trial procedures for the control period during the month before the time of inclusion".* Since the staff was not blinded to the allocation status and recruitment			
Domain 1b	2c: Cluster baseline imbalance?	Y	Baseline imbalances on individual level (see 1c). Additionally, the number of participants per cluster varies from 349 (Center 3) to 1,242 (Center 8) (one centre per sequence).			

Table S1 (continues)

Brennan et al, 2023		Australia (East Asia and Pacific)	Adults	Intensive care unit (clinic patients)	Disease prevention / screening (The nurse-led intervention incorporated a multicomponent delirium prevention protocol that targeted risk factors for delirium).		
		Signalling question / Response			RoB Justification	Domain	Overall
Domain	1a	1a: Allocation sequence random?	Y		*"The random order in which the clusters crossed over into the training, and then the intervention period, was performed externally with the assistance from colleges from Griffith University, Queensland".* *From protocol: "This information will be known to the central research team; however, given the close geographical proximity of the ICUs and study design, allocation concealment cannot be guaranteed."*	High	High
		1b: Allocation sequence concealed?	PN				
		1c: Any baseline differences?	PY		There are 5/13 general characteristics of the participants (including age, comorbidities, planned surgery status, APACHE III [risk stratification score]) which is different between pre- and post-intervention groups. No information to compare date on cluster level is available.		
	1b	2a: Recruitment prior to randomisation?	N		*"All patients admitted to the four adult ICUs during the study period were included in the study, except for patients among which delirium assessment was impractical..."* The design of study is cross-sectional due to the short duration of admission to ICUs. It is impossible to identify all participants before randomisation.	High	
		2b: Signs of selection bias?	PY		The medical staff has been aware of the intervention allocation, thereby allocation subversion cannot be excluded. *"All patients admitted to the four adult ICUs during the study period were included in the study, except for patients among which delirium assessment was impractical: (i) patients at the end of life, and not expected to survive 24 h; (ii) patients not staying in the ICU for at least 24 h; (iii) patients with acute or chronic neurological conditions that may prevent assessment of delirium (traumatic brain injury, intracerebral haemorrhage, ischaemic stroke, central nervous system infection, hypoxic brain injury, hepatic encephalopathy, severe mental disability, serious receptive aphasia, dementia)."* Although all eligible participants were included, the assessment of eligibility is based on somewhat subjective criteria, therefore the risk of selectivity in recruitment cannot be completely ruled out.		
		2c: Cluster baseline imbalance?	NI		Baseline imbalances on individual level (see 1c). Additionally, there were 1,184 participants in pre-intervention group, and 1,434 patients in post-intervention group.		

Table S1 (continues)

Study ID	Country (Region)	Age group	Settings	Intervention (description)			
Chen et al., 2023	United States (North America)	Adults	Hospitals (clinic patients)	Educational (standardized Stir-up Regimen (deep breathing, coughing, repositioning, mobilization [moving arms/legs], assessing and managing pain and nausea) within the first 30 minutes of arrival in the postanesthesia care unit (PACU), with a goal of decreasing recovery time in the immediate postanesthesia period).			
		Signalling question / Response		RoB Justification		Domain	Overall
Domain	1a	1a: Allocation sequence random?	NI	*"A prospective longitudinal design was used, with control and intervention groups that randomized at the group level, not the individual patient level".* Research team included a biostatistician who produced a randomisation sequence.		Some concerns	Some concerns
		1b: Allocation sequence concealed?	NI				
		1c: Any baseline differences?	PN	Baseline characteristics are mainly well-balanced (Table 1) and are likely to result from a random error. No between cluster baseline characteristics are provided. *"The demographic distribution between intervention and control groups was statistically and clinically similar. Patient variables (age, gender, race, ethnicity, BMI, and ASA score) were analyzed in the adjusted model and did not make a significant difference to time in minutes between control and intervention outcomes (Table 2)."*			
	1b	2a: Recruitment prior to randomisation?	PN	Due to the nature of the setting (postanaesthesia units), advanced recruitment would be infeasible.		Some concerns	
		2b: Signs of selection bias?	NI	No information available if the recruitment team was blinded to the allocation status.			
		2c: Cluster baseline imbalance?	PN	Baseline characteristics are mainly well-balanced (Table 1) and are likely to result from a random error. *"The number of patients in each PACU is not the same, which is a consequence of the stepped wedge cluster RCT design. The three PACUs had similar surgical volumes during the study (PACU A 33%, PACU B 32%, PACU C 34%) with consistent weekday surgery numbers (Table 1)."*			

Study ID	Country (Region)	Age group	Settings	Intervention (description)			
Coronado et al., 2023	United States (North America)	Adults	Primary healthcare facilities (primary care clinics)	Disease prevention / screening (Mailed fecal immunochemical test (FIT) for colorectal cancer screening).			
		Signalling question / Response		RoB Justification		Domain	Overall
Domain	1a	1a: Allocation sequence random?	PY	*On the basis of prior research (29), we stratified clinics based on colorectal cancer screening rate (>40%;-≤40%) and the proportion of patients who listed Spanish as their preferred language (>70; ≤70%), then the project statistician randomized them into two wedges. Wedge 1 clinics crossed over from the standard mailed FIT program to the enhanced mailed FIT program in June of 2018; wedge 2 clinics crossed over in June of 2019. All clinics were randomized on May 31, 2018.*		Some concerns	High
		1b: Allocation sequence concealed?	NI				
		1c: Any baseline differences?	PN	Baseline individual characteristics are well-balanced. The number of clusters allocated to each sequence is similar. Intervention group (n= 16,934) is noticeably larger than the control group (n=10,651).			
	1b	2a: Recruitment prior to randomisation?	N	*"Across the baseline, year 1, and year 2, time periods, we identified 27,585 instances where these criteria were met at the 15 participating clinics (7,101 at baseline, 8, 31 in year 1, and 11,553 in year 2). Some adults met criteria in more than one time period".*		High	
		2b: Signs of selection bias?	PY	*For practical reasons, neither researchers nor centralized clinic outreach staff were blinded to clinic-level or patient-level randomization assignment. Clinic staff in a centralized outreach department mailed FIT kits to all qualifying adults in each time period*. Since the recruitment team was not blinded to allocation status, selection bias cannot be rules out.			
		2c: Cluster baseline imbalance?	PY	Baseline individual characteristics are well-balanced. Intervention group (n= 16,934) is noticeably larger than the control group (n=10,651). No inter-cluster differences presented, but the numbers of participants in the second sequence is smaller than in the first sequence.			

Table S1 (continues)

Study ID	Country (Region)	Age group	Settings	Intervention (description)
Currier et al., 2023	United States (North America)	Adults	Primary healthcare facilities (clinic patients)	Management tool (community paramedicine implemented in 2 rural counties in reducing nonemergent ED use among a sample of Medicaid beneficiaries with complex medical conditions and a history of high ED utilization)

Domain		Signalling question / Response		RoB Justification	Domain	Overall
Domain	1a	1a: Allocation sequence random?	NI	*"Patients were clustered at the clinic level; the unit of randomization was the clinic. The sequence order of the 4 clinics' transition from control to intervention was determined pragmatically based on clinic readiness to assume new workflow processes associated with the intervention".* No details on randomisation process were provided.	Some concerns	Some concerns
		1b: Allocation sequence concealed?	NI			
		1c: Any baseline differences?	NI	No baseline data for Intervention and Control groups separately are presented. The number of clusters per sequence was equal (1 cluster per sequence).		
	1b	2a: Recruitment prior to randomisation?	PY	*"All participants were enrolled during the recruitment phase and followed longitudinally for 20 months".*	Low	
		2b: Signs of selection bias?	NA	Since all participants were enrolled before the randomisation of clusters, there was no risk of selection bias (not being assessed according to Cochrane RoB tool 2.0).		
		2c: Cluster baseline imbalance?	PY	No baseline data for Intervention and Control groups separately are presented. There was noticeably different number of participants per cluster: clinic 1 (n = 61), clinic 2 (n = 9), clinic 3 (n = 20), clinic 4 (n = 12) (one clinic per sequence).		

Study ID	Country (Region)	Age group	Settings	Intervention (description)
De Oliveira et al., 2023	Brazil (South America)	Adults	Hospitals (clinic patients)	Disease prevention / screening (Risk stratification and pregnancy delivery strategy)

Domain		Signalling question / Response		RoB Justification	Domain	Overall
Domain	1a	1a: Allocation sequence random?	PY	*"Randomization was undertaken by the trial statistician through a computer-generated blocking list at the start of the trial. The timing of implementation of the intervention to each center was revealed only 30 days before the implementation phase to prevent changes in practice before the implementation".*	Low risk	High
		1b: Allocation sequence concealed?	PY			
		1c: Any baseline differences?	PN	*"There were no substantial differences in the baseline characteristics between the 2 trial groups."*		
	1b	2a: Recruitment prior to randomisation?	N	*"… eligibility was assessed after randomization in an unblinded way, which may have introduced a risk of bias".*	High	
		2b: Signs of selection bias?	PY	The recruiting staff was not blinded to allocation status.		
		2c: Cluster baseline imbalance?	Y	On a cluster level, the total number of deliveries, number of preterm preeclampsia cases, total number of preeclampsia cases, and total number of preterm deliveries were noticeably different between intervention and control group, and well as between sites (Table S2, S3, Supplementary material).		

Table S1 (continues)

Study ID	Country (Region)	Age group	Settings	Intervention (description)			
Dijkstra et al., 2023	Netherlands (Europe)	Adults	ICU (relatives of ICU patients)	Educational (standardized program to facilitate family participation inpatient communication, amusement/distraction, comfort, personal care, breathing, mobilization, and nutrition)			
	Signalling question / Response			RoB Justification	Domain	Overall	
Domain 1a	1a: Allocation sequence random?	NI		*"The start of the intervention at center level was randomized, blinding was not possible".* Authors state that the randomisation has taken place, but no further details were given.	High	High	
	1b: Allocation sequence concealed?	NI					
	1c: Any baseline differences?	PY		Baseline differences in clinically important variables on individual patients' level (such as admission type, OCU length of stay, days being mechanically ventilated) (Table 1). Additionally, there were noticeably fewer participants in the Intervention group (n=73) rather than in Control group (n=233).			
Domain 1b	2a: Recruitment prior to randomisation?	PN		*"The start of the intervention at center level was randomized, blinding was not possible".*	High		
	2b: Signs of selection bias?	PY		Since the blinding did not occur, this knowledge of allocation status might have affected the recruitment.			
	2c: Cluster baseline imbalance?	NI		No inter-clusters comparisons for baseline characteristics were provided.			

Study ID	Country (Region)	Age group	Settings	Intervention (description)			
Eismann et al., 2023	United States (North America)	Children	Primary healthcare facilities (pediatric primary care practice)	Disease prevention / screening (Parent Connext, a positive parenting program that integrates screening and co-located parent coaching within pediatric primary care)			
	Signalling question / Response			RoB Justification	Domain	Overall	
Domain 1a	1a: Allocation sequence random?	Y		*"Randomization was performed at the cluster level by a research coordinator using random number generation to determine which year each practice would implement Parent Connext".*	Some concerns	Some concerns	
	1b: Allocation sequence concealed?	NI					
	1c: Any baseline differences?	PN		Baseline characteristics between clusters (paediatric sites) and interventions arms (intervention and control groups) are quite well-balanced (based on Table 1, Table 2). Number of patients in control period was 21,779, and during intervention stage the number was similar (n=21,734).			
Domain 1b	2a: Recruitment prior to randomisation?	PN		The study used retrospective patient's records.	Low		
	2b: Signs of selection bias?	PN		*"Retrospective chart review of all patients under age 8 who attended the six practices during the study period from January 1, 2017 to December 31, 2019 was performed at the conclusion of the study. The entire practice populations under age 8 were included in order to better understand practice-wide effects of the program… Patients under age 8 were studied because screening went up to age 6, and patients could be in the study period for up to 2 years afterward. Also, the majority of patients referred for parent coaching tend to be under age 8. The medical system's Institutional Review Board approved this study and waived informed consent".*			
	2c: Cluster baseline imbalance?	PN		Baseline characteristics between clusters (paediatric sites) and interventions arms (intervention and control groups) are quite well-balanced (based on Table 1, Table 2).			

Table S1 (continues)

Study ID	Country (Region)	Age group	Settings	Intervention (description)		
Fournaise et al., 2013	Denmark (Europe)	Adults	Care home and their analogues (older adults receiving home-based care)	Disease prevention / screening (The PATINA algorithm and decision-support tool alerts home-based-care nurses to older adults at risk of hospitalisation).		

Domain		Signalling question	Response	RoB Justification	Domain	Overall
Domain	1a	1a: Allocation sequence random?	PY	*"Computer-generated randomisation by use of Stata (version 16) was done by a statistician not associated with the project." "It was not possible to mask the nurses or the managers of the local area teams, but the dates for crossover to the intervention were masked from the area teams and the project management until 1 month before study inclusion".*	Some concerns	Some concerns
		1b: Allocation sequence concealed?	PY			
		1c: Any baseline differences?	PY	The baseline characteristics between Intervention and Control groups mostly balanced but statistically significantly different between clinically important variables (comorbidity index, number of hospital admissions).		
	1b	2a: Recruitment prior to randomisation?	PN	The recruitment was ongoing as the study design was cross-sectional, and recruitment of all participants prior to the randomisation would be infeasible.	Some concerns	
		2b: Signs of selection bias?	PN	Highly unlikely as all eligible dwellers were included in the study. *"It was not possible to mask the nurses or the managers of the local area teams, but the dates for crossover to the intervention were masked from the area teams and the project management until 1 month before study inclusion".*		
		2c: Cluster baseline imbalance?	PY	Baseline characteristics are imbalances between municipalities (i.e., cluster combinations).		

Study ID	Country (Region)	Age group	Settings	Intervention (description)
Gago et al., 2023	United States (North America)	Children	Geographical areas (agencies serving children and their families)	Educational (Parents Connect for Healthy Living (PConnect) - parents education to encourage healthy weight parenting practices, as a means of promoting healthy child growth and development, while recognizing the broader impact of contextual factors, including the social determinants of health, on parenting)

Domain		Signalling question	Response	RoB Justification	Domain	Overall
Domain	1a	1a: Allocation sequence random?	PY	*"Using a stepped wedge design the Head Start programs were randomly assigned by the study's data manager, and with oversight from the study statistician, to one of three intervention start times".*	Some concerns	Some concerns
		1b: Allocation sequence concealed?	NI			
		1c: Any baseline differences?	PY	Although no inter-clusters comparisons presented, no baseline differences which could be caused by improper randomisation process were noticed. However, there were statistically significant differences between intervention and control groups in ethnicity and education. The number of children identified during passive enrolment was 3368 in Control group and 1631 in Intervention group. Later. 2650 and 1361 children were eligible for screening measurement in Control and Intervention groups respectively. Overall, 2579 children from the Control group and 1318 from the Intervention group were included in the analysis.		
	1b	2a: Recruitment prior to randomisation?	PN	Infeasible in terms of settings (study is an open-cohort).	Low risk	
		2b: Signs of selection bias?	PN	*"Families were informed during enrollment each year of the health measures collected and of the potential for de-identified data for all children to be used for quality improvement or research purposes. Passive consent procedures were used for the aforementioned measures; that is, families were given the opportunity to opt out of the assessments prior to measurement".* Low risk of selection bias.		
		2c: Cluster baseline imbalance?	NI	No inter-clusters comparisons were presented.		

Table S1 (continues)

Study ID	Country (Region)	Age group	Settings	Intervention (description)				
Guterud et al., 2023	Norway (Europe)	Adults	Other healthcare facilities (ambulance stations)	Diagnostic (use of the National Institutes of Health Stroke Scale (NIHSS) by paramedics in the ambulance to improve communication with the hospital, augment triage, and enhance diagnostic accuracy of acute stroke)				
		Signalling question / Response			RoB Justification		Domain	Overall
Domain	1a	1a: Allocation sequence random?		PY	*"The cluster randomisation was based on sequential conversion of the ambulance stations from control to intervention; hence, paramedics received intervention training based on station affiliation. The cluster sequence was randomised using a simple lottery draw. Five numbered notes were drawn to select the cluster activation order. This draw was done independently by the administration of the Prehospital Division at OUH and was masked from the research team. A few weeks in advance of activation, the research team was informed which cluster should receive training".*		Some concerns	High
		1b: Allocation sequence concealed?		PY				
		1c: Any baseline differences?		PY	Individual baseline characteristics are well-balanced. However, the number of patients in the Intervention group was 447, and 354 were in the Control group.			
	1b	2a: Recruitment prior to randomisation?		PN	Recruitment of all participants before the cluster randomisation was infeasible due to the intervention and medical condition (acute stroke) characteristics.		High	
		2b: Signs of selection bias?		PY	*"A few weeks in advance of activation, the research team was informed which cluster should receive training. It was not possible to conceal the allocation of clusters due to the nature of the intervention".* Therefore the risk of selection bias cannot be ruled out.			
		2c: Cluster baseline imbalance?		PN	Cluster size varied from 45 to 93 paramedics per site which should have probably be included into consideration during randomisation. However, no information of the number of patients per cluster is available.			

Study ID	Country (Region)	Age group	Settings	Intervention (description)				
Gyedu et al., 2023a	Ghana (Africa)	Adults and children	Hospitals (clinic patients)	Diagnostic (trauma intake form (TIF) as a quality improvement intervention) (in emergency units (EU) health service providers (EHSP))				
		Signalling question / Response			RoB Justification		Domain	Overall
Domain	1a	1a: Allocation sequence random?		PY	*"The TIF was then introduced to 2 nearby hospitals, through simple random sampling, after training the EHSPs on its use. Sampling was done using STATA'S random number generator. Groups of 2 nearby hospitals were randomized together for ease of study management. The TIF was introduced sequentially to randomly selected groups of 2 hospitals every 3.5 months in a stepped-wedge manner with a total of 5 periods.*		High	High
		1b: Allocation sequence concealed?		NI				
		1c: Any baseline differences?		PY	Baseline characteristics are imbalanced on clinical variables (consciousness level on EU arrival, EU outcome, seriousness of injury) (Table 1). Number of patients in intervention and control groups is comparable.			
	1b	2a: Recruitment prior to randomisation?		PN	Identification of all participants before the randomisation of the clusters would be infeasible due to the ongoing retirement process for patients with acute medical condition (trauma).		Low	
		2b: Signs of selection bias?		PN	No information regarding whether the recruiting staff was blinded to the allocation status, only a statement "*Within-cluster contamination was minimized by exposing EHSPs to the TIF only after their hospital was randomized for TIF introduction and cluster allocation bias was avoided by exposing all the hospitals to the TIF with their order of exposure determined randomly" "Recruitment bias was avoided by obtaining consent from EHSPs to be observed. This allowed inclusion of all injured patients arriving at the EU."*			
		2c: Cluster baseline imbalance?		NI	No between-cluster comparisons were provided.			

Table S1 (continues)

Study ID	Country (Region)	Age group	Settings	Intervention (description)				
Gyedu et al., 2023b	Ghana (Africa)	Adults and children	Hospitals (clinic patients)	Diagnostic (trauma intake form (TIF) as a quality improvement intervention) (in emergency units (EU) health service providers (EHSP))				
		Signalling question / Response		RoB Justification			Domain	Overall
Domain	1a	1a: Allocation sequence random?	Y	*"The TIF was then introduced to a simple randomly selected group of two nearby hospitals after training the EHSPs on its use. Groups of two hospitals that were close geographically were randomized together to decrease costs of travel for training and study management (that is, for logistical reasons). Training of EHSPs in use of the TIF was done by the PI and lasted 1 day at each hospital. The TIF was similarly introduced sequentially to the next randomly selected group of two hospitals every 3.5 months in a stepped-wedge fashion until study completion at 17.5 months"*			High	High
		1b: Allocation sequence concealed?	NI					
		1c: Any baseline differences?	PY	Baseline characteristics are imbalances on clinical variables (mechanism and intent of injury, injury seriousness) (Table 2). Number of patients in intervention and control groups is comparable.				
	1b	2a: Recruitment prior to randomisation?	PN	Identification of all participants before the randomisation of the clusters would be infeasible due to the ongoing recruitment process for patients with acute medical condition (trauma).			Some concerns	
		2b: Signs of selection bias?	NI	No information regarding whether the recruiting staff was blinded to the allocation status.				
		2c: Cluster baseline imbalance?	NI	Baseline characteristics are imbalances on clinical variables (mechanism and intent of injury, injury seriousness) (Table 2); however, the comparison is available between children and adults without details per intervention arm or between clusters.				

Study ID	Country (Region)	Age group	Settings	Intervention (description)				
Hastings et al., 2023	United States (North America)	Adults	Hospitals (clinic patients)	Educational (STRIDE is a supervised walking program for older adult inpatients that includes a 1-time gait and balance assessment followed by daily supervised walks for the duration of the hospital stay)				
		Signalling question / Response		RoB Justification			Domain	Overall
Domain	1a	1a: Allocation sequence random?	Y	*"Enrollment and randomization occurred in 2 distinct blocks of 4, with 2 hospitals randomly assigned to each sequence".* Recruitment and randomisation was performed in two batches, therefore there is a higher possibility of properly concealed allocation sequence.			Low	Low
		1b: Allocation sequence concealed?	PY					
		1c: Any baseline differences?	PN	Individual baseline characteristics are mainly well-balanced.				
	1b	2a: Recruitment prior to randomisation?	PN	Infeasible due to the nature of the patient characteristics (aged 60 years or older who were community dwelling and admitted for 2 or more days to a participating medicine ward)			Low	
		2b: Signs of selection bias?	PN	No patient-level consent was required to be included in study analyses. Patients' medical records and electronic datasets were used for the analyses. *"Clinicians treating hospitalized patients at enrolled sites were not blinded; however, they were not informed of study eligibility criteria nor of study outcomes being assessed".*				
		2c: Cluster baseline imbalance?	PN	Cluster size varied within sequences and time periods but overall was comparable.				

Table S1 (continues)

**Study ID**	**Country (Region)**	**Age group**	**Settings**	**Intervention (description)**
Irvine et al., 2023	United States (North America)	Adults	Other healthcare facilities (original CCP provider agencies)	Other (healthcare organisation: HIV care coordination program (CCP))

		Signalling question / Response		RoB Justification	Domain	Overall
Domain	1a	1a: Allocation sequence random?	Y	*Sites were matched to maximize similarity on characteristics plausibly related to the outcome: site type, primary location, and program size. After finalizing the pairs with input from team members, the lead analyst used a random-number generator in Excel to determine site assignments within pairs.*	Some concerns	Some concerns
		1b: Allocation sequence concealed?	PY			
		1c: Any baseline differences?	PY	*"A total of 960 clients met trial eligibility criteria: 176 in Period 0, 390 in Period 1, and 394 in Period 2. The 9 early-implementing sites accounted for 531 eligible clients (55.3%),while the 8 late-implementing sites accounted for 429 (44.7%).Clients at late-implementing and early-implementing sites resembled one another closely on ART status, baseline CD4 and VL levels. However, clients at late-implementing sites were more often reported to be younger than 45 years, men, White or Asian, residents of Brooklyn or Queens, stably housed, never incarcerated…" (etc).*		
	1b	2a: Recruitment prior to randomisation?	PY	Taking into account the nature of the design, it was almost achieved by: The trial-eligible enrollment window for each nine-month implementation period was restricted to the first 5 months, permitting 4 months of outcome observation per enrollee before the next transition/step. Clients were not individually recruited for the trial but were enrolled in the original or revised CCP depending on the site and period in which they presented for services. Following intention-to-treat principles, all clients determined retrospectively to meet trial eligibility criteria were included in the analysis	Low	
		2b: Signs of selection bias?	NA	Since all participants were enrolled before the randomisation of clusters, there was no risk of selection bias (not being assessed according to Cochrane RoB tool 2.0).		
		2c: Cluster baseline imbalance?	PY	BL characteristics between early and later implantation are imbalanced (see 1c)		

**Study ID**	**Country (Region)**	**Age group**	**Settings**	**Intervention (description)**
Iverson et al., 2023	United States (North America)	Adults	Primary healthcare facilities (Veterans Health Administration facilities)	Disease prevention / screening (intimate partner violence screening programs in primary care)

		Signalling question / Response		RoB Justification	Domain	Overall
Domain	1a	1a: Allocation sequence random?	PY	*"Of these, 12 sites enrolled through a project letter of agreement signed by local leadership and were assigned to 1 of 2 waves of the stepped-wedge using a site-balancing algorithm. This algorithm increases the likelihood that waves were broadly similar on key facility-level characteristics (e.g., size and complexity) while retaining the benefits of randomization".* After randomisation, one site from Wave 1 (out of six) and two sites from Wave 2 (out of six) withdrew from the trial.	High	High
		1b: Allocation sequence concealed?	NI			
		1c: Any baseline differences?	PY	Presented demographic individual baseline characteristics are balanced, although no clinically important variables were given. Number of eligible participants in control (pre-IF) stage was 2,272, and 5,149 participants in the intervention (IF) stage.		
	1b	2a: Recruitment prior to randomisation?	PN	*The study was cross-sectional by design, therefore no recruitment prior to randomisation was feasible.*	Some concerns	
		2b: Signs of selection bias?	PN	*"Medical record data for all women receiving care at the participating primary care clinics (i.e., all eligible women) at the nine sites in the 3 months before IF (pre-IF assessment period) and 9 months after the start of IF (IF assessment period) were included in analyses".*		
		2c: Cluster baseline imbalance?	PY	Participants' baseline characteristics vary noticeably between sites (Appendix Table 2). Range of eligible participants per site was 97-1849 (Wave 1) and 51-875 (Wave 2).		

Table S1 (continues)

Study ID	Country (Region)	Age group	Settings	Intervention (description)			
Jones et al., 2023	Australia (East Asia and Pacific)	Adults	Primary healthcare facilities (general practices)	Educational (Intervention included (1) electronic-technology tool extracting data from general practice electronic medical records and generating graphs and lists for audit; (2) education regarding chronic disease and the electronic-technology tool; (3) assistance with quality improvement audit plan development, benchmarking, monitoring and support)			
		Signalling question / Response		RoB Justification		Domain	Overall
Domain	1a	1a: Allocation sequence random?	PY	*"Simple randomisation occurred after all practices had been recruited. Once randomisation had taken place, practices were advised of the trial schedule. Practices were enrolled by a project officer who subsequently assigned practices to their sequence as per randomisation schedule. This was an open intervention with no blinding".* The paper does not specify who generated the randomisation sequence, and it sounds as identification of clusters, randomisation and revealing the allocation sequence to clusters have been performed by the same person (project officer) (which is unlikely but details are missing).		High	High
		1b: Allocation sequence concealed?	PN				
		1c: Any baseline differences?	PN	Based on Table 3, individual baseline characteristics were mostly balanced. There were certain discrepancies in rates of diagnostic testing completed as per guideline recommendations (chronic kidney disease and type 2 diabetes).			
	1b	2a: Recruitment prior to randomisation?	N	*Rather than one cohort present at baseline that is then followed all the way through, the participants in each period consisted of all eligible patients in the participating practices at that time point (a cross-section).*		Some concerns	
		2b: Signs of selection bias?	PN	*" Eligibility criteria for patients within the practices included: age ≥18 years and being an active patient (attendance at the same general practice≥three times in the past 24 months – a definition used by the Royal Australian College of General Practitioners [RACGP]35). De-identified data for study analysis were extracted every 16 weeks from the general practice EMRs using the Pen CAT data extraction tool36 on site at each of the practices by practice staff"*			
		2c: Cluster baseline imbalance?	PY	The number of clusters per sequence was similar (n=2-3), but the number of eligible patients per sequence varied (from 5,509 to 13,688).			

Study ID	Country (Region)	Age group	Settings	Intervention (description)			
Kohler et al., 2023	Kenya (Africa)	Adults and children	Primary healthcare facilities (clinics)	Educational (Standardized patient (SP) training is anchored in Kolb's experiential learning and Bandura's social learning theories; health providers learn through cycles of concrete experiences (mock clinical encounters with SPs), observation, and feedback after the encounters, in a safe and controlled setting)			
		Signalling question / Response		RoB Justification		Domain	Overall
Domain	1a	1a: Allocation sequence random?	Y	*"The study statistician generated the randomization assignment for each clinic using Microsoft Excel". "Facilities were not informed which wave they were in until it was time to schedule that wave"*		Low	Low
		1b: Allocation sequence concealed?	PY				
		1c: Any baseline differences?	PN	Although p-values (or any other measures of statistical significance) are not presented, individual baseline characteristics appear to be balanced.			
	1b	2a: Recruitment prior to randomisation?	PN	*The study design was cross-sectional – "Retention in care and clinical outcomes were assessed via an audit of EMR charts from YLHIV 10–24 years of age, and a subset of YLHIV from each facility were also enrolled to complete patient satisfaction and risk behavior surveys after each training wave".*		Low	
		2b: Signs of selection bias?	PN	*Facilities were not informed which wave they were in until it was time to schedule that wave*			
		2c: Cluster baseline imbalance?	PN	The number of participants per wave (sequence) was similar (except for the last wave affected by COVID-19 pandemic). Data per cluster/sequence were not presented.			

Table S1 (continues)

Study ID	Country (Region)	Age group	Settings	Intervention (description)			
Kosters et al., 2023	Netherlands (Europe)	Adults	Care home and their analogues (residents of nursing homes)	Educational (Mobile Soundscape Appraisal and Recording Technology sound awareness intervention (MoSART+) on NPS in nursing home residents with dementia. The goal was to raise awareness about soundscapes and their influence on residents.)			
	Signalling question / Response			RoB Justification		Domain	Overall
Domain 1a	1a: Allocation sequence random?	Y		*"...there were 5 periods (steps), with the allocation sequence being randomly computer-generated and concealed from the nursing homes until we began preparing for the intervention in each home (eg, ambassador selection)".*		Some concerns	Some concerns
Domain 1a	1b: Allocation sequence concealed?	PY					
Domain 1a	1c: Any baseline differences?	PY		*"... duration of stay was notably longer for residents enrolled at baseline than for those included after the trial had started. Workload was highest and job satisfaction was lowest among the nursing staff in the nursing home that did not implement the intervention".* were slightly more participants in the control group (98 versus 86 in the intervention group).			
Domain 1b	2a: Recruitment prior to randomisation?	N		After baseline, 7 new residents were included, of which 4 had a follow-up assessment (study was an open cohort).		Some concerns	
Domain 1b	2b: Signs of selection bias?	PN		Although the study was an open cohort, all eligible residence had become trial participants.			
Domain 1b	2c: Cluster baseline imbalance?	PY		The cluster size varied from 12 to 27 (Table 1). *"There were some important differences between individual nursing homes at baseline. Residents in one home had more severe NPS and lower QoL; in another, a higher proportion used psychotropic drugs; and in 2 nursing homes, residents had longer durations of stay"*			
Krauss et al., 2023	United States (North America)	Adults	Geographic areas (apartment buildings grouped based on geographical proximity).	Disease prevention / screening (The intervention had three components designed to reduce falls among residents: (1) fall risk awareness for staff and residents, (2) resident fall risk screening, and (3) a HARP for participants at high risk of falling.)			
	Signalling question / Response			RoB Justification		Domain	Overall
Domain 1a	1a: Allocation sequence random?	Y		*"The study statistician randomly ordered the five clusters using a computer-generated random allocation sequence. "Agency staff (including building managers) were provided with a schedule that reflected the study timeline and randomized sequence of intervention deliver" "Due to the nature of the study design and intervention, there were no blinding procedures.*		High	High
Domain 1a	1b: Allocation sequence concealed?	PN					
Domain 1a	1c: Any baseline differences?	NI		Individual baseline characteristics were poorly balanced between clusters (Table 2), but no data comparing intervention and control groups were provided.			
Domain 1b	2a: Recruitment prior to randomisation?	N		*The study had a cross-sectional design - "All residents were eligible to participate in screening when their site was randomly assigned to cross over into the intervention period".*		High	
Domain 1b	2b: Signs of selection bias?	PY		*"Agency staff (including building managers) were provided with a schedule that reflected the study timeline and randomized sequence of intervention deliver" – since the staff has not been blinded to allocation sequence and recruitment was ongoing, the risk of selection bias cannot be completely ruled out.*			
Domain 1b	2c: Cluster baseline imbalance?	PY		The number of buildings clusters was similar (2-3 buildings). The number of recruited participants varies from 42 (cluster 4) to 70 (cluster 2) (one cluster per sequence).			

Table S1 (continues)

Study ID	Country (Region)	Age group	Settings	Intervention (description)			
Lambert et al., 2023	United Kingdom (Europe)	Children	Hospitals (clinic patients)	Other (haemodialysis device for babies under 8kg, the Newcastle Infant Dialysis Ultrafiltration System (NIDUS), compared with the current options of peritoneal dialysis (PD) or continuous venovenous hemofiltration (CVVH))			
		Signalling question / Response		RoB Justification		Domain	Overall
Domain	1a	1: Allocation sequence random?	Y	*"The randomisation was restricted so that one large site and one small site was allocated to each sequence, to mitigate the risk of a sequence with very low recruitment and to control the variation in power between different allocations. The Senior Trial Manager produced a list in which each of the symbols A, B and C was associated with one of the large sites in an arbitrary order. The Senior Trial Statistician used the base function sample in R to produce a random permutation of the symbols A, B and C, with the first element of the permuted list being allocated to sequence 1, the second to sequence 2 and the last to sequence 3. This allocation, with one upper case and one lower case letter allocated to each sequence was passed to the Senior Trial Manager, who was able to form the random allocation of sites to sequences by substituting the actual site names for the symbols".*		High	High
		2: Allocation sequence concealed?	NI				
		3: Any baseline differences?	PY	*"The baseline characteristics were similar in control and intervention groups".* However, mean age was noticeably higher, and more males were recruited in the intervention group (eTable 2a). The number of clusters per sequence was identical (n=2). There were noticeably more recruited patients in sequence 3 (Figure 1). The number of participants in the control group was 62, and in the intervention group n=35.			
	1b	1: Recruitment prior to randomisation?	PN	Due to the nature of intervention (haemodialysis need might be acute) and study design (cross-sectional) this was infeasible to identify and recruit all participants prior to the cluster randomisation.		High	
		2: Signs of selection bias?	NI	The study was unblinded; no information regarding whether the recruiting staff was blinded the allocation status; however, it might be unlikely.			
		3: Cluster baseline imbalance?	PY	See 1a.3.			

Study ID	Country (Region)	Age group	Settings	Intervention (description)			
Leal et al., 2023	Canada (North America)	Adults	Hospitals (clinic patients)	Pharmacological (administering probiotics to prevent Clostridioides difficile infection (CDI) among patients receiving therapeutic antibiotics)			
		Signalling question / Response		RoB Justification		Domain	Overall
Domain	1a	1a: Allocation sequence random?	PY	*"Due to concerns that starting the study at FMC as the largest and most complex facility would be logistically challenging, an a priori decision was made to start FMC last, and the remaining facilities were randomly staggered using a random number generator in R version 3.3.1 software".*		High	High
		1b: Allocation sequence concealed?	PN				
		1c: Any baseline differences?	PN	Total admissions, patient days, and antibiotic courses per period that are not expected to be influenced by the intervention are described in Table 1			
	1b	1: Recruitment prior to randomisation?	PN	Since the patients' turnover rate was high, the design of the study was cross-sectional, and the identification and recruitment of all participants prior to the randomisation of clusters was infeasible.		Some concerns	
		2: Signs of selection bias?	NI	No information regarding whether the recruiting team was blinded to the allocation status; however, it is highly unlikely that the recruiting team was not aware of the allocation status after the cross-over period.			
		3: Cluster baseline imbalance?	PN	Total admissions, patient days, and antibiotic courses per period that are not expected to be influenced by the intervention are described in Table 1			

Table S1 (continues)

Study ID	Country (Region)	Age group	Settings	Intervention (description)
Lee AK et al., 2023	United States (North America)	Adults	Primary healthcare facilities (patients in primary care practices)	Disease prevention / screening (The SPARC intervention – alcohol-related prevention and treatment).

Domain		Signalling question	Response	RoB Justification	Domain	Overall
Domain	1a	1: Allocation sequence random?	Y	*"The study biostatistician completed stratified random assignment using a computer-generated list of random numbers; within each year (y1 vs y2), each site had equal probability of assignment to each wave. The 22 practices were randomized as 19 sites because clinical leaders requested that 3 pairs of nearby practices be randomized together as 3 sites".*	Some concerns	Some concerns
		2: Allocation sequence concealed?	NI			
		3: Any baseline differences?	PN	Although, there are no p-values as well as authors comments regarding baseline characteristics available, individual baseline characteristics seem to be sufficiently balanced. The number of clusters per sequence was similar (n1=9, n2=10). The number of patients in the intervention and control groups was comparable (Figure 2).		
	1b	1: Recruitment prior to randomisation?	N	The study design was cross-sectional: "Patients could contribute data to usual care and/or SPARC intervention periods depending on when they visited primary care."	Low	
		2: Signs of selection bias?	PN	Although the actual recruitment of all participants was infeasible, the data was used from ALL participants in each practice. Waiver of consent was used.		
		3: Cluster baseline imbalance?	PN	See 1a.3.		

Study ID	Country (Region)	Age group	Settings	Intervention (description)
Lee DS et al., 2023	Canada (North America)	Adults	Hospitals (clinic patients)	Management tool (a point-of-care algorithm to stratify patients with acute heart failure according to the risk of death).

Domain		Signalling question	Response	RoB Justification	Domain	Overall
Domain	1a	1a: Allocation sequence random?	Y	*"A covariate-constrained randomization method was used ... with regard to status as a teaching hospital (there were five academic hospitals and five community hospitals) and the annual volume of patients with heart failure. An independent statistician implemented the randomization scheme. All research personnel and hospitals were unaware of the hospital sequence assignments until 4 months before implementation of an intervention; at that point, staff were informed about the intervention, and the implementation team performed training before crossover".*	Low	Low
		1b: Allocation sequence concealed?	Y			
		1c: Any baseline differences?	N	*"The patients who were enrolled during the two phases were well matched with respect to baseline characteristics; there was a high percentage of patients who had a history of heart failure in both groups. The trial population was representative of patients with heart failure in the general population; the distributions according to age, sex, and race or ethnic group were similar to those observed in population-based studies. No patients were no lost to follow-up".*		
	1b	2a: Recruitment prior to randomisation?	PN	The study design was cross-sectional – "Patients were eligible for enrollment in the trial if they were at least 18 years of age and presented to the emergency department with acute heart failure." – due to the medical condition and settings, recruitment prior to randomisation was not feasible.	Low	
		2b: Signs of selection bias?	PN	*"Trained chart abstractors, who were unaware of the hospital sequence assignments, obtained detailed clinical information from hospital records - including demographic characteristics, presenting signs and symptoms, medical history, left ventricular function, and laboratory test results and biomarker levels - and confirmed the clinical diagnosis of heart failure with data from hospital records for all trial participants".*		
		2c: Cluster baseline imbalance?	N	See 1a.3		

Table S1 (continues)

Study ID	Country (Region)	Age group	Settings	Intervention (description)			
Llewelyn et al., 2023	United Kingdom (Europe)	Adults	Hospitals (clinic patients)	Other (decision aid: to clarify the level of diagnostic uncertainty at antibiotic initiation)			
	Signalling question / Response			RoB Justification		Domain	Overall
Domain 1a	1a: Allocation sequence random?	Y		*"Eligible sites were randomised by use of a computer-generated list by the trial statistician (ASW), including the pilot sites (one block of three sites) and main trial sites (six blocks of six sites and one block of seven sites), to an intervention implementation date". "To avoid contamination, complete information about the intervention and allocation sequence was concealed from the site until the point of randomisation, when sites were told that their randomised implementation date was 12 weeks in the future, ensuring that all sites had 12 weeks for implementation preparation."*		Low	Some concerns
	1b: Allocation sequence concealed?	Y					
	1c: Any baseline differences?	NI		Individual baseline characteristics for intervention and control groups are not given. Seven additional clusters were recruited to replace those that withdrew after randomisation (Figure 1).			
Domain 1b	2a: Recruitment prior to randomisation?	PN		All ongoing eligible admissions have been included in the analysis.		Some concerns	
	2b: Signs of selection bias?	PN		*"Since the intervention targeted prescribers on acute general medical wards and used electronic health records to ascertain patient-level outcomes, the study population was defined using the consultant specialty codes that were most often used to admit adult general medicine inpatients (appendix p 45).21 Sites were asked to exclude patients who opted out of having their health records used for research purposes (appendix pp 3–4)."*			
	2c: Cluster baseline imbalance?	PY		The number of admissions varied noticeably between clusters (median = 38045, IQR = 26008-56294, range = 11416 - 77946).			

Study ID	Country (Region)	Age group	Settings	Intervention (description)			
Ma et al., 2023	Multiple (Brazil, China, India, Mexico, Nigeria, Pakistan, Peru, Sri Lanka, Viet Nam, Chile)	Adults	Hospitals (clinic patients)	Management tool (a goal-directed care bundle incorporating protocols for early intensive blood pressure lowering and management algorithms for hyperglycaemia, pyrexia, and abnormal anticoagulation, implemented in a hospital setting)			
	Signalling question / Response			RoB Justification		Domain	Overall
Domain 1a	1a: Allocation sequence random?	Y		*"Eligible hospital sites were randomly assigned into three sequences (with four periods) by use of a computer-generated list by the trial statistician (QL) using permuted blocks, stratified by country and the projected number of patients to be recruited per site during the study period (<80, 80-160, and >160 patients). To avoid contamination, details of the intervention, sequence, and allocation periods were concealed from sites until they had completed the usual care control periods. A period of 7-10 days was used to allow sites time for their staff to receive training before transitioning from usual care to the intervention period".*		Low	Some concerns
	1b: Allocation sequence concealed?	Y					
	1c: Any baseline differences?	PN		Baseline demographic, medical history, and the clinical and brain imaging characteristics of patients were well balanced between the treatment groups. The number of clusters per sequence was similar (n=37-43). Number of patients per site per period is reasonably balanced (Figure 1).			
Domain 1b	2a: Recruitment prior to randomisation?	PN		The study design was cross-sectional, so identification and recruitment of all participants prior to the randomisation of clusters was infeasible.		Some concerns	
	2b: Signs of selection bias?	NI		All eligible hospital patients were included in the analysis. However, informed consent was required. No information whether the recruiting staff was blinded was available.			
	2c: Cluster baseline imbalance?	PN		Number of participants per cluster was balanced, no baseline characteristics per cluster were available.			

Table S1 (continues)

Study ID	Country (Region)	Age group	Settings	Intervention (description)
Marcy et al., 2023	Multiple (Cambodia, Cameroon, Côte d'Ivoire, Mozambique, Uganda, Zambia)	Children	Hospitals (clinic patients)	Disease prevention / screening (systematic tuberculosis detection in young children with severe pneumonia in countries with high incidence of tuberculosis)

Domain		Signalling question	Response	RoB Justification	Domain	Overall
Domain	1a	1: Allocation sequence random?	Y	*"The unit of randomisation was the cluster (hospital). Randomisation was stratified by the estimated country tuberculosis incidence rate, classified as either high or very high. The trial statistician at the University of Bordeaux, France, established the randomisation sequence before the start of the trial, using a computer-generated random sequence. The research teams and hospitals were masked to the randomisation order up to 5 weeks before the switch date".*	Low	Low
		2: Allocation sequence concealed?	Y			
		3: Any baseline differences?	PN	*"Overall, demographic characteristics were similar in the control and the intervention groups.... Pneumonia was more severe in the control group... There were more children with severe acute malnutrition in the intervention group than in the control group. There was a higher proportion of children receiving antibiotics in the intervention group than in the control group. Children were hospitalised for a median duration of 5 days with no difference between the study groups".* There were slightly more children enrolled on the control period (n=1401 in control and n=1169 in the intervention).		
	1b	1: Recruitment prior to randomisation?	PN	Identification of all participants before the randomisation was infeasible due to the study design and medical condition. :	Low	
		2: Signs of selection bias?	PN	*"Any child aged between 2-59 months presenting to the emergency units, intensive care unit, or paediatric departments of the selected hospitals with signs and symptoms of pneumonia were screened for trial eligibility".*		
		3c: Cluster baseline imbalance?	NI	No data per cluster were available.		

Study ID	Country (Region)	Age group	Settings	Intervention (description)
Martinez-Gutierrez et al., 2023	United States (North America)	Adults	Hospitals (clinic patients)	Management tool (automated computed tomography (CT) angiogram interpretation coupled with secure group messaging in endovascular stroke therapy EVT workflows)

Domain		Signalling question	Response	RoB Justification	Domain	Overall
Domain	1a	1: Allocation sequence random?	PY	*The order in which the clusters were activated was determined in a random fashion with 1 exception: the academic CSC was decided to be the final activation, as it was felt that additional time would be needed to prepare for this activation given the large number of residents and fellows involved in patient care at this CSC.*	High	High
		2: Allocation sequence concealed?	PN			
		3: Any baseline differences?	PN	Individual baseline characteristics were well-balanced (Table 1). There were slightly more patients in the control (n=140) than in the intervention group (n=103) (Figure 1).		
	1b	1: Recruitment prior to randomisation?	PN	Recruitment of all patients was infeasible prior to the randomisation.	Some concerns	
		2: Signs of selection bias?	PN	All eligible patients were included in the trial. Consent was waived.		
		3: Cluster baseline imbalance?	PY	The number of participants per cluster per sequence varied from 22 (cluster 2) to 105 (cluster 4) (Figure 2).		

Table S1 (continues)

Study ID	Country (Region)	Age group	Settings	Intervention (description)			
Maunder et al., 2023	Canada (North America)	Adults	Hospitals (staff)	CBT / behaviour intervention (The personalized self-awareness feedback (PSAF) intervention: The enriched survey provided personalized feedback about coping behaviours, interpersonal problems, moral distress, and attachment style, based on the relevant scores, during COVID-19 pandemic.)			
		Signalling question / Response		RoB Justification		Domain	Overall
Domain	1a	1a: Allocation sequence random?	PY	*"Randomization of the time of availability of PRC was facilitated by assigning clinical units and departments to five randomization groups. To balance randomization group composition, the following procedure was followed. First, units were categorized by acuity: high acuity (e.g., ICU, emergency department), other patient care, or non-patient care. Within acuity categories, units were assigned to randomization groups to balance the number of participants and the proportion of occupation types within groups as much as possible and to minimize unintended early participant exposure to PRC interventions (e.g., by assigning geographically or organizationally related units to the same group). The order of availability of PRC intervention was then determined using an online randomization tool, and the groups were labelled A (first) to E (fifth)".*		Some concerns	High
		1b: Allocation sequence concealed?	NI				
		1c: Any baseline differences?	PN	Individual baseline characteristics are well balanced between Intervention / Control groups (Supplementary File 3).			
	1b	2a: Recruitment prior to randomisation?	NI	Cohort was recruited to take part in longitudinal study, and then in stepped-wedge trial, but no explicit information whether the randomisation occurred after the recruitment were provided.		High	
		2b: Signs of selection bias?	PY	Participants were the staff members, and "*Staff were not blind to whether PRC was available*." Participation was voluntary. therefore, there might be differences between participants and those who decided not to participate.			
		2c: Cluster baseline imbalance?	PY	The number of patients per cluster ranged noticeably (from 57 in Groups B&C to 153 in Group E) (Supplementary File 4).			

Study ID	Country (Region)	Age group	Settings	Intervention (description)			
Midgard et al., 2023	Norway (Europe)	Adults	Hospitals (clinic patients)	Pharmacological (opportunistic treatment of hepatitis C infection)			
		Signalling question / Response		RoB Justification		Domain	Overall
Domain	1a	1a: Allocation sequence random?	Y	*Allocation was computer-generated and stratified according to expected cluster size (small, medium, large) to keep high HCV prevalence clusters separated regarding the timing of the intervention. The sequences were prepared by a statistician not involved in enrollment and kept in closed opaque envelopes. Concealment of a new step in the sequence was made available to the researchers on the day of transition and immediately disclosed to the clinical staff at the relevant cluster.*		Low	High
		1b: Allocation sequence concealed?	PY				
		1c: Any baseline differences?	PN	Baseline characteristics were similar during intervention and control conditions and throughout the trial (however, p-values were not provided) (Table 1).			
	1b	2a: Recruitment prior to randomisation?	N	*"The trial had several limitations. First, because recruitment was done with study personnel and participants knowing the treatment allocation, potentially influencing screening activity and participation, the trial is at risk of selection bias between the intervention conditions". The trial had a cross-sectional design.*		High	
		2b: Signs of selection bias?	PY	See 1b.1.			
		2c: Cluster baseline imbalance?	PN	The number of patients and their characteristics were comparable between clusters (Supplementary Table 3).			

Table S1 (continues)

Study ID	Country (Region)	Age group	Settings	Intervention (description)			
Mohan et al., 2023	United States (North America)	Adults	Hospitals (physicians and their patients in acute care hospitals)	CBT / behaviour intervention (theory-based video game designed to increase autonomous motivation for Advanced Care Planning)			
	Signalling question / Response			RoB Justification		Domain	Overall
Domain 1a	1a: Allocation sequence random?	Y		*"Using a schema developed in R, one team member (AJO) stratified hospitals into eight blocks and randomized each hospital per block to the step in which they were invited to receive the intervention". "At the start of each step (to accommodate the oscillating stresses from COVID-19), we sequentially contacted the chief of the hospitalist services of each hospital."*		Low	Some concerns
Domain 1a	1b: Allocation sequence concealed?	PY					
Domain 1a	1c: Any baseline differences?	PN		Patients' baseline characteristics are balanced (Table 1). The number of patients in the intervention and control groups are comparable.			
Domain 1b	2a: Recruitment prior to randomisation?	NI		There is no direct information whether the randomisation was conducted before the recruitment took place. *The trial had a cross-sectional design.*		Some concerns	
Domain 1b	2b: Signs of selection bias?	NI		For physicians - it is possible that they were aware of the allocation status (no information available if the recruiting staff was blinded to the allocation status. For patients' data – all patients data was used (waived consent).			
Domain 1b	2c: Cluster baseline imbalance?	PN		The number of patients per cluster varies between sequences but similar per period (Figure 1).			

Study ID	Country (Region)	Age group	Settings	Intervention (description)			
Mutale et al., 2023	Zambia (Africa)	Adults and children	Primary healthcare facilities (rural health centres and their surrounding communities)	Educational (BHOMA was a complex health system intervention comprising intensive clinical training and quality improvement measures, support for commodities procurement, improved community outreach, and district level management support).			
	Signalling question / Response			RoB Justification		Domain	Overall
Domain 1a	1: Allocation sequence random?	Y		*"Randomization was done by a statistician (JL) based in London and not involved in implementation, who generated a random implementation schedule using Stata version 15 (StataCorp LLC; College Station, TX). The order of roll out of the intervention could not be blinded".*		High	High
Domain 1a	2: Allocation sequence concealed?	PN					
Domain 1a	3: Any baseline differences?	PN		Individual baseline characteristics for control and intervention groups are given in percentages but appear to be well-balanced (Table 2). The number of person-years contributing to the control and intervention periods is noticeably different (49,240 and 74,530 respectively) (Figure 3) (was caused by unexpectedly low mortality rates and decision to double the sample size during rounds 2&3).			
Domain 1b	1: Recruitment prior to randomisation?	PN		Baseline community survey and health facility survey on voluntary basis taken before the start of first intervention step. The cross-sectional nature of the trial implies continuous recruitment (for each survey round); therefore, the risk of bias cannot be ruled out (particularly with unmasked recruiting team to the allocation sequence).		High	
Domain 1b	2: Signs of selection bias?	PY		Since the *"The order of roll out of the intervention could not be blinded"*, recruiting team might have been aware of the allocation status.			
Domain 1b	3: Cluster baseline imbalance?	NI		No cluster-specific data are given.			

Table S1 (continues)

Study ID	Country (Region)	Age group	Settings	Intervention (description)			
Najafpour et al., 2023	Iran (Middle East)	Adults	Hospitals (clinic patients)	Educational (Multiple fall-prevention interventions)			
		Signalling question / Response		RoB Justification		Domain	Overall
Domain	1a	1a: Allocation sequence random?	Y	*"Randomization of 18 wards was conducted 4 weeks before the start of the study using a matched-pair cluster-randomized technique. Information about unique intervention sequence was revealed by a researcher 2 to 3 days before each transition period started for each pair by an expert researcher who designed the randomization process for the research team to prepare wards for intervention".*		Some concerns	High
		1b: Allocation sequence concealed?	Y				
		1c: Any baseline differences?	PY	The number of participants in the control phase was n=2407, and in the intervention n=4031. The number of participants per cluster varied within and between sequences (Figure 1). No identification of statically significant difference is given in Table 1; however, individual baseline characterises seem to be quite imbalanced.			
	1b	2a: Recruitment prior to randomisation?	N	Throughout the trial, all patients admitted to participating units were assessed by nurses.		High	
		2b: Signs of selection bias?	PY	*"Interventions in this research study sought to motivate patients and the staff to improve their knowledge about implementing other study interventions. Therefore, it was not feasible to blind staff or patients in this trial".*			
		2c: Cluster baseline imbalance?	PY	See 1a.3.			

Study ID	Country (Region)	Age group	Settings	Intervention (description)			
Pathare et al., 2023	India (South Asia)	Adults	Primary healthcare facilities (patients in primary health centers)	Educational (Atmiyata, a community champion led psychosocial intervention for common mental disorders)			
		Signalling question / Response		RoB Justification		Domain	Overall
Domain	1a	1a: Allocation sequence random?	Y	*"Participants were randomly selected from the master screening list using a computer-generated random method". "the data collection team is separate from the intervention team and blind to the treatment status. "[from protocol]*		Some concerns	Some concerns
		1b: Allocation sequence concealed?	PY				
		1c: Any baseline differences?	PY	*"Compared to intervention participants, participants in the control condition were older, less educated, and more likely to be employed".*			
	1b	2a: Recruitment prior to randomisation?	N	This study used a cross-sectional design, where outcome data is derived from different participants at each period. A random sample of 56 participants was selected from each cluster for each period.		Low	
		2b: Signs of selection bias?	PN	Based on the recruitment process described in the protocol, recruiting staff were not aware of the allocation status.			
		2c: Cluster baseline imbalance?	NI	*No cluster-specific data were given.*			

Table S1 (continues)

Study ID	Country (Region)	Age group	Settings	Intervention (description)			
Politi et al., 2023	United States (North America)	Adults	Primary healthcare facilities (clinic patients in outpatient urology clinics)	Other (decision aid: the impact of a conversation-based decision aid containing information about low-risk prostate cancer management options and their relative costs)			
	Signalling question / Response			RoB Justification		Domain	Overall
Domain	1a	1a: Allocation sequence random?	Y	*"The principal investigator and study coordinator enroled clusters. The study statistician, masked to cluster identity, generated the randomisation schedule and randomly allocated clusters (urologic surgeons) to the intervention sequences with a simple randomisation approach using R statistical software. Across the control and intervention arms, the study coordinator enroled consecutive eligible patients who provided informed consent to participate".*		Some concerns	Some concerns
		1b: Allocation sequence concealed?	NI				
		1c: Any baseline differences?	PN	Individual baseline characteristics are mainly balanced (Table 1). The number of participants per intervention arm is slightly different but comparable (Control n=51, Intervention n=66)			
	1b	2a: Recruitment prior to randomisation?	NI	There is no direct information whether the randomisation was conducted before the recruitment took place. *The trial had a cross-sectional design.*		Some concerns	
		2b: Signs of selection bias?	PN	*"Across the control and intervention arms, the study coordinator (K. P.) enroled consecutive eligible patients who provided informed consent to participate."*			
		2c: Cluster baseline imbalance?	PY	However, the number of participants per intervention arm per surgeon (cluster) is statistically significantly different (p<0.01) (Table 1).			

Study ID	Country (Region)	Age group	Settings	Intervention (description)			
Posthuma et al., 2023	Netherlands (Europe)	Adults	Hospitals (clinic patients)	Management tool (continuous wireless monitoring for high-risk early postsurgical patients in real time)			
	Signalling question / Response			RoB Justification		Domain	Overall
Domain	1a	1: Allocation sequence random?	NI	*"The stepped-wedge approach was chosen, as it prevents contamination that could arise from a learning effect when using a dual approach (wireless monitoring, whilst simultaneously randomly assigning patients to standard perioperative care in the same ward). To treat an equal number of patients in the intervention and control group in both hospitals, wards 1 and 4 were assigned to Amsterdam University Center and wards 2 and 3 were assigned to University Medical Center Utrech".*		High	High
		2: Allocation sequence concealed?	PN				
		3: Any baseline differences?	PY	The number of participants in the Intervention group (n=230) is noticeably smaller than in the Control (n=517) (Figure 2). *"… cancer was present in 58.4% of patients in the control group versus 70.0% patients in the intervention group. Diabetes Mellitus was observed in 16.1% of patients in the control group, versus 11.7% patients in the intervention group. In the control group, 54.4% of patients underwent major surgery, compared to 52.6% of patients in the intervention group. In total, 14 patients died within 90 days after surgery, 11 (2.2%) patients in the control group versus 3 (1.3%) patients in the intervention group"*			
	1b	1: Recruitment prior to randomisation?	N	Recruitment was continuous as *"To ensure an equal number of patients across the two study arms, a switch to the subsequent study phase was made after 30 study patients had been recruited in one study block".*		Some concerns	
		2: Signs of selection bias?	NI	No information whether the recruiting staff was blinded to the allocation status. However, it was unlikely that they were not aware of the ward allocation status.			
		3: Cluster baseline imbalance?	NI	No cluster-level data were given.			

Table S1 (continues)

Study ID	Country (Region)	Age group	Settings	Intervention (description)			
Raymond et al., 2023	United States (North America)	Adults	Geographical areas (ZIP codes)	Other (communicable disease control: automated vs. manual case investigation and contact tracing for pandemic surveillance)			
	Signalling question / Response			RoB Justification		Domain	Overall
Domain 1a	1a: Allocation sequence random?	Y		*"Randomization was done by the researchers with a random number generator prior to the study period. The allocation sequence was made available to SCC Public Health leadership, but not to those performing manual CICT or study participants".*		Low	Low
	1b: Allocation sequence concealed?	Y					
	1c: Any baseline differences?	PN		*"Ultimately, we note no systematic imbalances that could threaten the validity of our study".* There were slightly fewer participants in Cluster 2 in comparison to other clusters (Figure 1).			
Domain 1b	2a: Recruitment prior to randomisation?	PY		Clusters were recruited prior to randomisation; cases recruitment is impossible.		Low	
	2b: Signs of selection bias?	NA		Since all participants were enrolled before the randomisation of clusters, there was no risk of selection bias (not being assessed according to Cochrane RoB tool 2.0).			
	2c: Cluster baseline imbalance?	PN		*"Ultimately, we note no systematic imbalances that could threaten the validity of our study".* There were slightly fewer participants in Cluster 2 in comparison to other clusters (Figure 1).			

Study ID	Country (Region)	Age group	Settings	Intervention (description)			
Rogers et al., 2023	United States (North America)	Adults and children	Homeless shelters	Disease prevention (influenza testing at kiosks in homeless shelters)			
	Signalling question / Response			RoB Justification		Domain	Overall
Domain 1a	1a: Allocation sequence random?	Y		*"Nine shelters were randomized to the four sequences, with rerandomization at the start of each year using computer-generated randomization. Stratified randomization (youth vs. adult shelters) was performed to ensure that the family shelters (n = 3) were evenly distributed to three of the four sequences. All sites remained in the intervention condition for the remainder of the season once it had been introduced".*		Some concerns	High
	1b: Allocation sequence concealed?	PY					
	1c: Any baseline differences?	PY		Individual baseline characteristics are given without statistical significance indicators (e.g., p-values) but appear quite imbalanced (i.e., more males in intervention arm, and on average older participants in intervention arm (Table 1)). There were slightly more participants in the Intervention group (n=769) than in Control (n=514).			
Domain 1b	2a: Recruitment prior to randomisation?	PN		Infeasible due to the study design (cross-sectional study with continuous recruitment).		High	
	2b: Signs of selection bias?	PY		*"... selection biases may have occurred as the nature of the stepped-wedge cluster-randomized trial design does not allow for blinding of the intervention. Study participation may have been perceived as more desirable during intervention periods when immediate testing results and actionable intervention for illness episodes were made available".*			
	2c: Cluster baseline imbalance?	NI		No cluster-level data were given.			

Table S1 (continues)

Study ID	Country (Region)	Age group	Settings	Intervention (description)			
Schafthuizen et al., 2023	Netherlands (Europe)	Adults	Hospitals (nurses and their clinic patients)	Educational (a new evidence-based nursing oral care protocol in a Dutch university teaching hospital on both nurses' level of oral health-related knowledge and attitude as well as nurses' protocol adherence to oral care in ADL-dependent patients).			
		Signalling question / Response		RoB Justification		Domain	Overall
Domain	1a	1: Allocation sequence random?	Y	*"An independent statistician assigned randomly the order in which participating clusters started with the implementation phase. Each of the four clusters was made up of two or three clinical units on the same floor, with a total of 10 units (both internal medicine and surgery)".*		High	High
		2: Allocation sequence concealed?	NI				
		3: Any baseline differences?	PY	Individual baseline characterises for nurses were slightly imbalanced (in the Control group nurses were on average older and with more years of experiences (Table 1). The number of participating nurses in Control (n=226) and Intervention (n=283) groups was comparable. For patients, their individual baseline characteristics were balanced except for ward type (Table 2). The number of patients in Control (n=73) and Intervention (n=51) groups was similar.			
	1b	1: Recruitment prior to randomisation?	N	*"We included 509 nurses; 226 in the pre-implementation period and 283 in the post-implementation period".*		High	
		2: Signs of selection bias?	PY	"A convenience sample was recruited, consisting of nurses at work when the principal investigator visited the relevant unit and who were willing to provide consent". No information whether the recruiting staff was aware of the allocation status; however, it is highly likely that the staff was unblinded.			
		3: Cluster baseline imbalance?	NI	No cluster-level data were given.			

Study ID	Country (Region)	Age group	Settings	Intervention (description)			
Schweickert et al., 2023	United States (North America)	Adults	ICUs (clinic patients)	Educational (the mobilization intervention included 1) designation and posting of daily mobilization goals; 2) interprofessional closed-loop communication coordinated by each ICU's facilitator; and 3) performance feedback).			
		Signalling question / Response		RoB Justification		Domain	Overall
Domain	1a	1: Allocation sequence random?	Y	*"At the outset, we considered three small, closely located ICUs in one hospital to represent a single ICU and then randomly assigned the resultant 12 ICUs into six clusters of two ICUs each. We constrained the randomization such that each cluster included one ICU above the median annual volume of patients mechanically ventilated for >48 hours. Every 6 weeks, a new cluster adopted the intervention in a sequence determined by computerized random-number generation". "Given the nature of the interventions, clinicians and ICU administrators were aware of their ICU's study phase"*		High	High
		2: Allocation sequence concealed?	PN				
		3: Any baseline differences?	PY	Individual baseline characteristics are mainly balanced (Table 1). The number of patients per intervention arm is noticeably different (Control = 848, Intervention = 1069).			
	1b	1: Recruitment prior to randomisation?	PN	Infeasible due to continuous new hospitalisations.		Low	
		2: Signs of selection bias?	PN	*"All patients admitted to the 12 ICUs from March 4, 2019 through March 15, 2020 were eligible for STAND, given that the intervention was rolled out as a new standard of care for all admitted patients. Because the trial was conducted with a waiver of consent, patients and caregivers were unaware of the trial or the phase their ICU was in".*			
		3: Cluster baseline imbalance?	NI	No information about between cluster differences is provided.			

Table S1 (continues)

Study ID	Country (Region)	Age group	Settings	Intervention (description)
Shete et al., 2023	Uganda (Africa)	Adults	Primary healthcare facilities (community health centres)	Disease prevention / screening (the impact of a cash transfer intervention on completion of TB testing and treatment initiation).

		Signalling question / Response		RoB Justification	Domain	Overall
Domain	1a	1a: Allocation sequence random?	Y	*"Eligible health centres were randomised using a simple, unrestricted two-stage process. First, they were matched into clusters based on pre-randomisation data of patient volume. Second, clusters were randomly assigned into the sequence order in which they would switch into the intervention period during a stakeholder-led randomisation ceremony whereby health centre representatives chose a numbered ball from an opaque bag, indicating their sequence order". Owing to the nature of the intervention, masking of participants and providers was not possible.*	High	High
		1b: Allocation sequence concealed?	PN			
		1c: Any baseline differences?	PY	*"People evaluated during the pre-intervention and intervention periods were similar except for lower proportions with HIV (25.9% versus 42.0%) and confirmed TB (3.6% versus 8.1%) in the intervention period (table 1)".* There were almost as twice as many participants in the Intervention period (n=2212) than in the Control (n=1143).		
	1b	2a: Recruitment prior to randomisation?	PN	*"The trial employed a repeated cross-sectional design; each 1-month time period captured different people initiating evaluation for TB".*	Low	
		2b: Signs of selection bias?	PN	*"Eligible people were enrolled into the cash transfer intervention by health centre laboratory technicians at the time they submitted a sputum sample for TB testing. The intervention was introduced to relevant health centre staff during a 1 to 2 days of training at the beginning of the buffer month (supplementary methods)".* No information whether the recruiting staff was actually blinded to the allocation status. The trial was approved by the institutional review boards at Makerere University School of Public Health and the University of California San Francisco, and by the Uganda National Council for Science and Technology including a waiver of written informed consent for enrolling in the intervention and accessing routinely collected demographic and clinical information among all participants		
		2c: Cluster baseline imbalance?	NI	*No cluster-level data were given.*		

Study ID	Country (Region)	Age group	Settings	Intervention (description)
Shields et al., 2023	Australia (East Asia and Pacific)	Adults	Community gyms (people with disability and their student mentors)	CBT / behaviour intervention (FitSkills is an evidenced-based 12-week, a student-mentored, community-based exercise program for young people with disability)

		Signalling question / Response		RoB Justification	Domain	Overall
Domain	1a	1a: Allocation sequence random?	PY	*"A member of the research team (LP) not involved in recruitment, assessment or intervention delivery, randomised the order of the sites using web-based software".*	High	High
		1b: Allocation sequence concealed?	NI			
		1c: Any baseline differences?	PY	Individual baseline characteristics are given per cluster (no Intervention/Control group comparison available). No p-values given, but baseline characteristics between clusters are imbalanced (Table 1).		
	1b	2a: Recruitment prior to randomisation?	NI	The study had a cohort design. However, there was no available information whether the recruitment was performed before the randomisation.	Some concerns	
		2b: Signs of selection bias?	PN	Recruiting staff was not aware of the allocation status.		
		2c: Cluster baseline imbalance?	PY	There were fewer participants in Cluster B (n=39) in comparison to other clusters (n=52-59).		

Table S1 (continues)

Study ID	Country (Region)	Age group	Settings	Intervention (description)		
Siegal et al., 2023	Canada (North America)	Adults	ICUs (clinic patients)	Management tool (small-volume blood collection tubes to reduce transfusions in intensive care).		
	Signalling question / Response			RoB Justification	Domain	Overall
Domain 1a	1: Allocation sequence random?	Y		*"We used a computer-generated randomization schedule to determine the order in which ICUs would transition from standard-volume (4.0-6.0 mL) to small-volume (1.8-3.5 mL) tubes". "On the transition date, standard volume tubes were replaced with small-volume tubes. Random audits were done to evaluate adherence to allocated tubes."*	Low	Low
Domain 1a	2: Allocation sequence concealed?	Y				
Domain 1a	3: Any baseline differences?	PN		*"Baseline characteristics of the primary and secondary populations were generally similar before and after transition, with standardized differences of 0.1 or less for most parameters. The baseline characteristics of patients admitted during the transition delay and those admitted during other study periods were also similar".* No information regarding the baseline differences between clusters was available, the number of patients in intervention and control groups was similar (Intervention = 10261, Control = 10940).		
Domain 1b	1: Recruitment prior to randomisation?	PN		Due to the study setting (ICUs), the identification and recruitment of all participants prior to the randomisation is infeasible.	Low	
Domain 1b	2: Signs of selection bias?	PN		Data from all eligible participants were analysed: *"The population for the primary analysis included all patients admitted to the participating ICUs during the trial except the following: those admitted to the ICU for less than 48 hours, those admitted during one of the prespecified washout periods, or those admitted during the 4 periods in which implementation of the intervention was delayed due to the COVID-19 pandemic." "Research ethics boards at all participating centers approved the study under a waiver of individual patient consent due to the low-risk nature of the intervention and clinical use of both tube types"*		
Domain 1b	3: Cluster baseline imbalance?	NI		See 1a.3.		

Study ID	Country (Region)	Age group	Settings	Intervention (description)		
So et al., 2023	Hong Kong (East Asia and Pacific)	Children	Special needs school (children with autism)	CBT / behaviour intervention (robot dramas where two social robots modelled RJA [responses to joint attention] and verbal responses from the children with low-functioning autism were not required)		
	Signalling question / Response			RoB Justification	Domain	Overall
Domain 1a	1a: Allocation sequence random?	PY		*"... we adopted a stepped wedge design where children (N = 18) were randomly assigned into three tiers, with each tier receiving intervention at staggered time points (Tier 1 received the intervention earlier than Tiers 2 and 3)".*	Some concerns	Some concerns
Domain 1a	1b: Allocation sequence concealed?	NI				
Domain 1a	1c: Any baseline differences?	PN		"There was no significant difference in age among the three tiers". The number of children per cluster was the same (n=6). No other information is provided.		
Domain 1b	2a: Recruitment prior to randomisation?	Y		All identified and recruited children were randomly allocated to clusters.	Low	
Domain 1b	2b: Signs of selection bias?	NA		Since all participants were enrolled before the randomisation of clusters, there was no risk of selection bias (not being assessed according to Cochrane RoB tool 2.0).		
Domain 1b	2c: Cluster baseline imbalance?	PN		*"There was no significant difference in age among the three tiers. ... there were 6 children in each cluster, and all 18 children completed the trial".*		

Table S1 (continues)

Study ID	Country (Region)	Age group	Settings	Intervention (description)
Spies et al., 2023	Germany (Europe)	Adult	ICUs (clinic patients)	Management tool (The quality improvement intervention consisted of daily telemedical rounds guided by eight German acute ICU care QIs and expert consultation).

Domain		Signalling question	Response	RoB Justification	Domain	Overall
Domain	1a	1: Allocation sequence random?	Y	*"Before trial commencement, all 12 clusters were randomly allocated to one of three sequence groups of four units to switch from the control to the intervention according to a staggered timetable. The randomisation list defining the order of the treatment switch was generated by the independent trial statistician using a computer-generated algorithm (nQuery Advisor V.7., block size=3, without stratification by prespecified characteristics)". "Due to a 3-month training period before the sequential switch to the intervention period, ICU staff were not masked to the allocated sequence group and were notified prior to the study start about their crossover date. Since study personnel and patients knew when they were and were not engaged in the telemedical intervention, blinding was not possible."*	High	High
		2: Allocation sequence concealed?	PN			
		3: Any baseline differences?	PY	*"There were no major differences between both cohorts with respect to demographic characteristics and primary admission diagnosis. However, the severity of illness at baseline was lower for patients treated on standard of care vs on intervention.... On control condition, patients were more frequently admitted due to medical reasons and admitted from emergency medical services and wards, but fewer postoperative admissions were observed....* There were 10 clusters in each intervention group, but the number of participants in the control phase was n=414, and n=1048 for the Intervention phase.		
	1b	1: Recruitment prior to randomisation?	PN	Due to the study settings (ICUs) identification and recruitment of all study participants prior to the randomisation would be infeasible. Recruitment was continuous.	High	
		2: Signs of selection bias?	PY	*"Due to a 3-month training period before the sequential switch to the intervention period, ICU staff were not masked to the allocated sequence group and were notified prior to the study start about their crossover date. Since study personnel and patients knew when they were and were not engaged in the telemedical intervention, blinding was not possible".*		
		3: Cluster baseline imbalance?	PY	See 1a.3.		

Study ID	Country (Region)	Age group	Settings	Intervention (description)
Strombotne et al., 2023	United States (North America)	Adult	Hospitals (clinic patients)	Management tool (a mandate requiring providers to perform case reviews on opioid analgesic-prescribed patients at high risk of overdose/suicide).

Domain		Signalling question	Response	RoB Justification	Domain	Overall
Domain	1a	1: Allocation sequence random?	Y	*"All 140 VHA medical centers were included in the study and were allocated to one of two clusters (i.e., early versus late expansion of the "very high" risk cohort from top 1% to top 5%) using permuted block randomization. All medical centers were blinded to assignment...".*	Some concerns	Some concerns
		2: Allocation sequence concealed?	PY			
		3: Any baseline differences?	PY	*"The standardized differences suggest no major imbalances among the control and treatment participants for sex, age, race, marital status, homelessness, number of comorbidities, or underlying predicted risk scores. Of the 28 individual comorbidities examined, only standardized differences in alcohol abuse were greater than 0.10".* There were as twice as many participants in the control phase (n=32,197) than in the intervention phase (n=11,845).		
	1b	1: Recruitment prior to randomisation?	PN	*"The design of this trial involved an open cohort, in which a substantial number of individuals were identified and designated as "very high" risk on the first day of the trial, and other participants became eligible over the course of the study".*	Some concerns	
		2: Signs of selection bias?	PN	*"All medical centers were blinded to assignment and received the treatment by the end of the study (Figure 1). Each medical center entered the study on 4/18/2018 in the control condition and randomly switched to the treatment condition in two waves..."*		
		3: Cluster baseline imbalance?	NI	No information regarding inter-cluster differences was provided.		

Table S1 (continues)

Study ID	Country (Region)	Age group	Settings	Intervention (description)			
Sturt et al., 2023	Tanzania, Nigeria (Africa)	Adults	Primary healthcare facilities (clinic patients in primary care practices)	Management tool (the REaCH intervention comprised training and mobile data allowance provision for mobile phones to support remotely delivered primary care in Africa compared with no training and mobile data allowance.).			
	Signalling question / Response			RoB Justification	Domain	Overall	
Domain 1a	1a: Allocation sequence random?	Y		*"Following enrolment, clusters were assigned to one of ten (Nigeria) or one of seven (Tanzania) sequences of intervention rollout (figure 1) by the trial team member (EA) responsible for randomisation. The clusters were placed in a random order by generating a uniform random variable In Microsoft Excel for each cluster and placing in ascending order. The allocated sequence was sent to trial staff by the trial team member (EA) for implementation". "Health facilities and REaCH training facilitators were not masked to allocation sequence given the need to plan service delivery and prepare for training."*	High	High	
	1b: Allocation sequence concealed?	PN					
	1c: Any baseline differences?	PN		*"Characteristics of the monthly random sample in both countries were similar across intervention and control groups (table 1)".* Cluster sizes and total numbers were similar across both countries in the intervention and control phases.			
Domain 1b	2a: Recruitment prior to randomisation?	PN		The patients were identified in both cohorts after the randomisation (open cohorts – recruitment prior to randomisation would be infeasible).	Low		
	2b: Signs of selection bias?	PN		All eligible patients' data were used, and *"The open cohort were not asked for consent as approved by all ethics committees".*			
	2c: Cluster baseline imbalance?	PN		Cluster sizes and total numbers were similar across both countries in the intervention and control phases.			

Study ID	Country (Region)	Age group	Settings	Intervention (description)			
Sunner et al., 2023	Australia (East Asia and Pacific)	Adults	Care home and their analogues (residents of residential aged care facilities)	Other (decision aid: implementation and evaluation of a nurse-led intervention to augment an existing residential aged care facility outreach service with a visual telehealth consultation).			
	Signalling question / Response			RoB Justification	Domain	Overall	
Domain 1a	1a: Allocation sequence random?	Y		*"For implementation purposes, facilities were organised into eight clusters for randomisation, by a statistician using a computer-generated randomisation sequence, with each cluster comprising one ED and two RACFs; each ED was included in two clusters".*	Some concerns	Some concerns	
	1b: Allocation sequence concealed?	NI					
	1c: Any baseline differences?	PN		Demographic characteristics of participants appeared to be similar between pre and post-implementation periods. Cluster size information is not available, but there were 333 participants in the control phase and 421 in the intervention phase.			
Domain 1b	2a: Recruitment prior to randomisation?	N		Infeasible as the cases were ongoing (open cohort).	Low		
	2b: Signs of selection bias?	PN		All eligible cases were included. *"Individual consent was not required for RACF residents' participation in this project as care provision was an extension of an existing service (ACE) and considered part of usual care for the resident in the event of an acute health episode".*			
	2c: Cluster baseline imbalance?	NI		Cluster size information is not available.			

Table S1 (continues)

Study ID		Country (Region)		Age group	Settings	Intervention (description)		
The TRACE Study Investigators, 2023		Netherlands (Europe)		Adults	Hospitals (clinic patients)	Surgical (routine visits by an anaesthesiologist on postoperative days 1 and 3 added to standard care).		
		Signalling question / Response				RoB Justification	Domain	Overall
Domain	1a	1: Allocation sequence random?	PY			*"The order in which the hospitals started with the intervention was randomized using envelope drawing".*	Some concerns	High
		2: Allocation sequence concealed?	NI			It was said that envelope drawing was used, but no additional information regarding whether the envelopes were sealed / opaque / sequentially numbered.		
		3: Any baseline differences?	PN			*"Patient characteristics and types of surgery were balanced between groups, except for clinically meaningful differences in the prevalence of active cancer and renal disease".*		
	1b	1: Recruitment prior to randomisation?	PN			Based on the setting of postoperative care, patients would be identified after randomisation (continuous recruitment).	High	
		2: Signs of selection bias?	PY			Although no information given whether the recruiting staff was blinded to the allocation status, the possibility of selective recruitment cannot be ruled out. *"Patients were only included in the study after written informed consent was obtained. Participants were recruited by a member of the study team during the preoperative screening or pre-operative hospital admission".*		
		3: Cluster baseline imbalance?	PY			See 1a.3. One hospital discontinued after the randomisation and was replaced. The number of patients per hospital varies noticeably.		

Study ID		Country (Region)		Age group	Settings	Intervention (description)		
Toles et al., 2023		United States (North America)		Adults	Care home and their analogues (patients in skilled nursing facilities)	Educational (Connect-Home included a patient and caregiver booklet with transition plans and a medication list, a schedule for transition care planning, and an electronic health record template for individualizing instructions for home-based care).		
		Signalling question / Response				RoB Justification	Domain	Overall
Domain	1a	1: Allocation sequence random?	PY			*"The randomized allocation sequence of the six SNFs determined the timing of patient and caregiver enrollment at each site in three phases: (1) control: patients and caregivers received usual care, (2) pre-implementation: staff were trained, and (3) intervention: patients and their caregivers received Connect-Home. The study statistician (J.P.) stratified randomization to assign the six SNFs to treatment sequences using codes to conceal allocation".*	Low	Some concerns
		2: Allocation sequence concealed?	Y					
		3: Any baseline differences?	PN			*"Patient characteristics were balanced for age, gender, race, ethnicity, and Charlson score (Table 1). The number of patients in the two treatment groups across different sites was imbalanced, which is expected in a SW-CRT design whereby sites switched from control to the intervention condition at randomly assigned time points. Moreover, caregiver characteristics were balanced with the exception that caregivers in the control group had higher education"*		
	1b	1: Recruitment prior to randomisation?	PN			Infeasible in terms of study setting (continuous recruitment).	Some concerns	
		2: Signs of selection bias?	NI			There is no information whether the recruiting staff was blinded to the allocation status.		
		3: Cluster baseline imbalance?	NI			No data per cluster were provided.		

Table S1 (continues)

Study ID	Country (Region)	Age group	Settings	Intervention (description)			
Vis et al., 2023	Multiple (Europe and Australia)	Adults	Other healthcare facilities (internet services in routine mental health care organizations)	Other (software: the ItFits-toolkit for internet-based cognitive behavioural therapy).			
	Signalling question / Response			RoB Justification		Domain	Overall
Domain 1a	1a: Allocation sequence random?	Y		*"The clusters were randomly allocated with an interval of 3 months, at which the clusters crossed over from the control condition (IAU) to the experimental condition (ItFits-toolkit)".* From protocol: *"Implementation sites will be randomly allocated to one of six groups (two implementation sites per group) prior to the start of the study. Randomization will be conducted by a computerized random number generator using R".* From protocol: *"The allocation scheme can only be accessed by the central trial coordination team".*		Low	Low
Domain 1a	1b: Allocation sequence concealed?	Y					
Domain 1a	1c: Any baseline differences?	NI		The information was provided for all participants in total, no detailed information per intervention arm or per cluster is available.			
Domain 1b	2a: Recruitment prior to randomisation?	NI		The study design was a closed cohort, but no detailed information whether the requirement occurred prior to the randomisation of clusters was provided.		Low	
Domain 1b	2b: Signs of selection bias?	PN		From protocol: *"The allocation scheme can only be accessed by the central trial coordination team. Any other investigators and all study participants within the implementation sites will be blinded to the crossover sequence."*			
Domain 1b	2c: Cluster baseline imbalance?	NI		The information was provided for all participants in total, no detailed information per intervention arm or per cluster is available.			

Study ID	Country (Region)	Age group	Settings	Intervention (description)			
Volandes et al., 2023	United States (North America)	Adults	Hospitals (clinic patients)	Educational (video intervention and goals-of-care documentation)			
	Signalling question / Response			RoB Justification		Domain	Overall
Domain 1a	1a: Allocation sequence random?	Y		*"A set of uniform random numbers was generated and used to assign the order of paired clusters for intervention initiation".*		Some concerns	High
Domain 1a	1b: Allocation sequence concealed?	NI		There is no information describing when the sites were informed about the intervention stage start.			
Domain 1a	1c: Any baseline differences?	PN		Patients in the usual care and intervention groups were similar in baseline demographics. The number of clusters per arm (n=7) was identical. There were more participants in the intervention group (n=6023 for Intervention and n=4779 for Usual care).			
Domain 1b	2a: Recruitment prior to randomisation?	N		Due to the cross-sectional nature of the study, the recruitment of all eligible patients before randomisation was infeasible.		High	
Domain 1b	2b: Signs of selection bias?	PY		*"Newly admitted patients without documented GOC discussions were prioritized over patients with documented GOC recorded prior to the start of the intervention. The PCEs did not engage patients with a documented GOC discussion at admission or those who were receiving palliative care services".*			
Domain 1b	2c: Cluster baseline imbalance?	NI		No data per cluster were available.			

Table S1 (continues)

Study ID	Country (Region)	Age group	Settings	Intervention (description)			
Wang et al., 2023	China (East Asia and Pacific)	Adults	Hospitals (clinic patients)	Management tool (a quality improvement intervention on reperfusion treatment for patients with acute ischemic stroke).			
		Signalling question / Response		RoB Justification		Domain	Overall
Domain	1a	1a: Allocation sequence random?	Y	*"The cluster randomization was performed at the hospital level centrally using a computer-generated random number sequence. The other members of the study team and the selected sites were informed that they would cross over to the intervention period 1 month before each of the predefined steps to maintain allocation concealment while aiding in training logistics".*		Some concerns	Some concerns
		1b: Allocation sequence concealed?	Y				
		1c: Any baseline differences?	PY	Individual baseline characteristics are mainly well-balanced (Table 1). "*The baseline characteristics between the intervention and control periods were generally similar except for a lower proportion of patients with a medical history of stroke or TIA (difference, −8.5%; 95% CI, −10.1% to −6.9%), patients with prior use of antiplatelet agents*". The number of participants in intervention group (n=5689) is slightly smaller than in the control group (n=6443). The number of participants per sequence varied noticeable (Figure 1).			
	1b	2a: Recruitment prior to randomisation?	PN	Clusters were randomised, and then the patients were enrolled (continuous recruitment with cross-sectional design).		Some concerns	
		2b: Signs of selection bias?	PN	Recruiting staff was not aware of randomisation sequence.			
		2c: Cluster baseline imbalance?	PY	Number of patients varies within clusters and within sequences (Figure 1).			

Study ID	Country (Region)	Age group	Settings	Intervention (description)			
Wanyenze et al., 2023	Uganda (Africa)	Adults	Hospitals (clinic patients)	Educational (effect of midwife-provided orientation of birth companions on maternal anxiety and coping during labour).			
		Signalling question / Response		RoB Justification		Domain	Overall
Domain	1a	1: Allocation sequence random?	Y	*"Using a simple random technique, the principal investigator EWW generated a random sequence of the four hospitals. Numbers 1, 2, 3, and 4 were assigned to the different facilities (Mbale 1, Bududa 2, Muyembe 3, Manafwa 4). Using a random sequence generator, a sequence of "2, 4, 1 and 3" was generated. This sequence is what guided which facility crossed over first to the intervention period".*		High	High
		2: Allocation sequence concealed?	NI				
		3: Any baseline differences?	PY	Individual baseline characteristics were mainly well-balanced between intervention and control groups. However, they were statistically significantly different for some clinically important variables (cervical dilatation on admission). The number of participants in intervention and control groups are similar, but cluster size varied from (n=101-153).			
	1b	1: Recruitment prior to randomisation?	N	Participants were recruited after the randomisation (cross-sectional design).		High	
		2: Signs of selection bias?	PY	As the recruitment was after the randomisation, there was no information whether the recruiting staff was blinded to the allocation status. Study participant was voluntary and informed consent was required (as opposed to waived consent and inclusion of all participants).			
		3: Cluster baseline imbalance?	PY	The number of participants in intervention and control groups are similar, but cluster size varied from (n=101-153).			

Table S1 (continues)

Study ID	Country (Region)	Age group	Settings	Intervention (description)			
Wilson et al., 2023	United States (North America)	Adults	Hospitals (clinic patients)	Other (decision aid: an artificial intelligence (AI)/machine learning (ML) decision support tool for predicting patient need for palliative care services in the hospital).			
		Signalling question / Response		RoB Justification		Domain	Overall
Domain	1a	1a: Allocation sequence random?	PY	*"Using the stepped-wedge design, nursing units were randomly and sequentially crossed over from control to intervention until all units were receiving care with the decision support tool. In the stepped-wedge design clusters, in this case floor units, cross over randomly (computer generated) from the control or standard of care condition to the intervention condition in a staggered fashion". "Due to the pragmatic design and intervention, providers were unable to be blinded to whether they were in the intervention unit or control unit."*		High	High
		1b: Allocation sequence concealed?	PN				
		1c: Any baseline differences?	PY	Although no p-values or authors' commentary provided regarding baseline characteristics per group (Intervention / Control), overall, the patients in the Intervention group tended to have fewer comorbidities and rarer received palliative care previously (which might be a significant difference for clinically important variables). Additionally, there were 962 participants in the control group, and 755 in the intervention group.			
	1b	2a: Recruitment prior to randomisation?	N	Patients' recruitment was an ongoing process (due to the nature of the study settings) and occurred after randomisation.		High	
		2b: Signs of selection bias?	PY	Due to the pragmatic design and intervention, providers were unable to be blinded to whether they were in the intervention unit or control unit.			
		2c: Cluster baseline imbalance?	NI	No data per cluster were given.			

Study ID	Country (Region)	Age group	Settings	Intervention (description)			
Yang et al., 2023	China (East Asia and Pacific)	Adults	ICUs (clinic patients)	Management tool (the establishment of reasonable physical restraint process for ICU adult catheterized patients).			
		Signalling question / Response		RoB Justification		Domain	Overall
Domain	1a	1a: Allocation sequence random?	PY	*"The four ICU wards were numbered from high to low according to the floor (1, 2, 3, 4). An independent statistician wrote the four numbers into four pieces of paper with same size and put them into four identical blank envelopes. The intervention order was determined by drawing lots by the head nurse of each ICU".*		High	High
		1b: Allocation sequence concealed?	PN				
		1c: Any baseline differences?	PY	The length of stay is statistically significantly longer for patients in Intervention group (Table 1). Intervention and Control groups drastically vary in size across different ICUs, and consequently, the number of patients per sequence is quite different.			
	1b	2a: Recruitment prior to randomisation?	N	Patient recruitment occurred after the randomisation and was ongoing due to the study settings (relatively short stay in ICUs).		High	
		2b: Signs of selection bias?	PY	The staff was not blinded to the allocation sequence. Huge gap in the number of recruited participants between Control and Intervention groups.			
		2c: Cluster baseline imbalance?	PY	The length of stay is statistically significantly longer for patients in Intervention group (Table 1). Intervention and Control groups drastically vary in size across different ICUs, and consequently, the number of patient per sequence is quite different.			

Table S1 (continues)

Study ID	Country (Region)	Age group	Settings	Intervention (description)
Yeates et al., 2023	Canada (North America)	Children	Hospitals (administrative date from emergency departments)	Educational (the clinical pathway emphasized standardized assessment of risk for persistent post-concussive symptoms using the validated 5P clinical risk score, provision of consistent information about concussion to patients and families, and referral for outpatient follow-up based on the risk stratification tool).

Domain	Signalling question	Response	RoB Justification	Domain	Overall
1a	1a: Allocation sequence random?	PY	*"An independent biostatistician used a computerized algorithm to randomize the implementation sequence, which was revealed to all sites in September 2018. The trial began on February 1, 2019, and concluded on November 30, 2019, although administrative data were collected through February 2020 to ensure capturing all relevant episodes of care".*	High	High
1a	1b: Allocation sequence concealed?	PN			
1a	1c: Any baseline differences?	PY	Descriptive characteristics are mostly balanced (Table 1). *"[Participants] completed 3009 visits to the 5 sites (1023 before and 1986 after implementation) and 781 follow-up visits to outpatient settings".*		
1b	2a: Recruitment prior to randomisation?	N	Due to the cross-sectional nature of the study, identification of all participants before the randomisation was infeasible.	High	
1b	2b: Signs of selection bias?	PY	The randomisation sequence was revealed for all sites in advance.		
1b	2c: Cluster baseline imbalance?	PY	*"[Participants] completed 3009 visits to the 5 sites (1023 before and 1986 after implementation) and 781 follow-up visits to outpatient settings".*		

Study ID	Country (Region)	Age group	Settings	Intervention (description)
Ziegler et al., 2023	Germany (Europe)	Adults	ICUs (clinic patients)	Management tool (the Mobility Monitor (MoMo) is a technical device that records a patient's movements and transmits the data to a monitor. This study investigated the extent to which the MoMo sensor system, which records and visualises patients' movements in bed, supports nurses in performing pressure-relieving repositioning in neurological and neurosurgical intensive care units).

Domain	Signalling question	Response	RoB Justification	Domain	Overall
1a	1a: Allocation sequence random?	PY	*"This process was performed by opening a randomisation envelope at each ICU. To guarantee concealment of the randomisation, the envelopes were centrally prepared by the Clinical Trials Unit at the beginning of the study using the random number generator of the function uniform available in SAS version 9.2".*	Low	Some concerns
1a	1b: Allocation sequence concealed?	PY			
1a	1c: Any baseline differences?	PN	Individual baseline characteristics were mainly well-balanced.		
1b	2a: Recruitment prior to randomisation?	PN	Infeasible due to the relatively short duration of stay for patients in ICUs.	Some concerns	
1b	2b: Signs of selection bias?	PN	Although "*neither the study team nor the scientists who analysed the data were blinded*", "*ethics committee approved a waiver for obtaining informed consent*" and therefore all eligible patients were included in the study.		
1b	2c: Cluster baseline imbalance?	PY	Number of participants per sequence varies (302 and 506).		
