# Supplementary material for: Role of recruitment bias in stepped-wedge cluster randomised controlled trials: a systematic review
Source: BMJ Open. 2025 Nov 28;15(11):e096281. doi: 10.1136/bmjopen-2024-096281 (PMC12666225; doi:10.1136/bmjopen-2024-096281)
Supplement: Supplementary file 6 [file bmjopen-2024-096281.reviewer_comments.pdf]

PEER REVIEW HISTORY

BMJ Open publishes all reviews undertaken for accepted manuscripts. Reviewers are asked to complete a checklist review form and are provided with free text boxes to elaborate on their assessment. These free text comments are reproduced below.

ARTICLE DETAILS

Title (Provisional)

The role of recruitment bias in stepped-wedge cluster randomised controlled trials: a systematic review

Authors

Yakimova, Antonina; Wiggins, Fraser; Kanaan, Mona; Keding, Ada; Torgerson, David

---

VERSION 1 - REVIEW

**Reviewer**	**1**
**Name**	**Ryan Baumann, Mary**
**Affiliation**	**University of Wisconsin-Madison School of Medicine and Public Health, Population Health Sciences; Biostatistics and Medical Informatics**
**Date**	**21-Feb-2025**
**COI**	**None**

This manuscript presents a systematic review of 65 SW-CRTs with results published in 2023 and how their designs and recruitment processes may increase their risk of recruitment bias.

One of my primary concerns is that the authors are not very clear about their definitions of biases and their sources. For example, I am unclear by what the authors mean when they refer to “diKerential recruitment of participants” or “diKerential randomization processes” (page 2, paragraph 2) so I cannot assess the mechanisms by which they would contribute to recruitment bias. In addition, when the authors say “recruitment bias”, it is unclear whether they mean bias due to self-selection of the participants or recruitment tactics by the study team. While both may be categorized under the broad umbrella of “recruitment bias”, I’d argue that they are two distinct sources with quite diKerent mitigation strategies. I would appreciate if the authors would clarify these points, as I think it will make the narrative of the manuscript and the aims of the review more clear.

I outline my additional comments below in order of where they appear in the manuscript.

- Page 3, line 7: The authors should make more clear somewhere in the manuscript that unrestricted randomization in CRTs only guarantees balance of cluster-level baseline characteristics, and does not guarantee balance of individual-level characteristics.
- Page 2, line 46: “more sources of bias” – you’ve not yet mentioned any potential sources or risks of bias in SW-CRTs. I would consider outlining these first before moving into bias due to recruitment.
- Page 4, line 30: The authors should clarify what they mean by “study setting”, and how they may diKerent from the definition of the unit of randomization.
- Page 5, lines 3-4: I would suggest the authors use some other phrase for the total RoB in this review than “overall RoB” if they are only tallying it over Domain 1 – perhaps “overall Domain 1 RoB”.
- Page 5, lines 23-24: what is the diKerence between “low risk”, “high risk”, and “some concerns”. Is “some concerns” lower risk than “low risk”? Between low and high risk? In addition, why are “high risk” and “some concerns” grouped together in Figure 2?
- Page 6, line 8: What do the authors mean by “random tiers”?
- Page 7, line 39-40: I would not say that no problems were identified with the random allocation process. I believe what question 1a.1 gets at is whether random allocation happened. There may still issues with that process even though random allocation did occur.
- Page 7, line 42: Do the authors mean baseline diKerences in cluster-level characteristics or in individual-level characteristics?
- Page 7, lines 48-51: “More than a half of the papers (n = 40 out of 60 papers where baseline characteristics between intervention groups were presented, 67%) suggested diKerential identification and/or recruitment of individuals in clusters.” I’m unclear of what the authors mean by “diKerential identification and/or recruitment of individuals in clusters”.
- Page 8, lines 56-60: If the authors collected specific data on the masking of recruitment team members, I think that would be very useful to present. As it currently stands, it’s diKicult to disentangle the sources of recruitment bias in the trials reviewed – are the majority of trials at risk because randomization happens prior to recruitment? How common is masking of the recruitment teams?

**Reviewer**	**2**
**Name**	**Kolokythas, Nico**
**Affiliation**	**University of Wolverhampton, Nico Kolokythas**
**Date**	**15-Apr-2025**
**COI**	**None**

This systematic review addresses an important methodological concern in stepped-wedge cluster randomised trials (SW-CRTs): the risk of bias arising from recruitment and the randomisation process. The topic is timely and relevant, particularly given the growing use of SW-CRTs in policy-facing interventions where methodological rigour must be beyond reproach. The decision to focus solely on Domain 1 of the Cochrane RoB 2.0 tool for cluster RCTs—specifically subdomains 1a (randomisation process) and 1b (identification and recruitment of participants)—is both justifiable and appropriate. However, I am concerned that the narrow scope is not matched by the required depth or analytical clarity. Given the specific aim of the paper, the level of detail and critical synthesis in both the results and discussion is insufficient and therefore **major amendments** are needed.

Lack of Depth Despite Narrow Scope

The restriction of this review to only two subdomains of a single RoB domain naturally invites a more detailed treatment of the methodological issues at hand. However, the analysis presented is relatively superficial.

For instance, in Domain 1a:

- While it is noted that random allocation was confirmed in 62 out of 64 studies, there is no systematic breakdown of the method of randomisation (e.g. simple, stratified, constrained), nor is there clarity on how this was verified.
- Similarly, allocation concealment is discussed as being adequate in 75% of cases, but there is no explanation of how this judgement was reached. Did the authors report use of centralised randomisation or sealed envelopes? This detail is essential in a review focused on bias.

In Domain 1b:

- The authors report that 75% of studies recruited participants after randomisation, and that around one-third had evidence of selective recruitment. However, the operational definitions of "selective recruitment" and "differential identification" are vague.
- It is unclear how masking of recruitment staff was determined, or whether this was based on explicit statements, indirect inference, or assumptions. Given the centrality of this point to Domain 1b, the lack of transparency here undermines the strength of the RoB judgements.

Underuse of Study-Level Examples

The review would be considerably strengthened by highlighting examples of both good and poor practice. For instance:

- In the paper by *Irvine et al.* (ref. 34), the authors restricted the enrolment window for each implementation period, allowing sufficient follow-up time while limiting the risk of time-related confounding. This is a pragmatic strategy worth highlighting as good practice.
- Conversely, in *Gyedu et al.* (refs. 31, 32), substantial baseline imbalances in both comorbidity and hospital admissions are reported. The review mentions these issues briefly but does not evaluate whether these were adjusted for or addressed analytically. Nor is there sufficient discussion of whether such imbalances reflect differential recruitment, structural design weaknesses, or both.

Without such illustrations, the risk of bias assessment remains abstract and potentially unconvincing to a critical reader.

Overreliance on Baseline Imbalance as a Proxy

The discussion often implies that baseline imbalance signals recruitment bias. While this may be true in some cases, it conflates a symptom with a mechanism. Imbalances can arise due to a range of factors, including small cluster numbers, time-varying site characteristics, or inadequate randomisation methods. Unless tied directly to unmasked recruitment or inappropriate allocation procedures, imbalance alone does not confirm a flaw in Domain 1a or 1b.

The review would benefit from explicitly distinguishing between:

- Structural/design-driven imbalances (e.g. seasonality, site variation),
- Procedural flaws (e.g. unmasked recruitment, foreknowledge of allocation),
- Chance variation due to underpowered designs.

This nuance is currently missing.

Missed Opportunity for Thematic Synthesis

Beyond reporting frequencies, the review does little to extract cross-cutting themes from the data. For example:

- How often did studies mitigate risks through automatic enrolment, waiver of consent, or analytic adjustment?
- Was there a pattern in risk of bias by study setting, region, or type of intervention?
- Were certain journals or trialists more consistent in reporting allocation methods?

A simple table listing mitigation strategies by study would offer far more value than the current results section provides.

Weak and Inconclusive Final Discussion

Given the findings—particularly that over 40% of trials were at high risk of recruitment or randomisation bias—the final conclusion feels underpowered. The claim that “some bias is unavoidable” is true, but it lacks critical edge. The review does not draw a line between inherent design constraints and avoidable methodological weaknesses. Nor does it offer concrete recommendations to funders, journal editors, or ethics committees on improving the quality of SW-CRT design and reporting.

Consider using this opportunity to advocate for:

- Improved pre-registration of randomisation and recruitment protocols,
- Mandatory reporting of allocation concealment methods in SW-CRTs,
- Development of a tailored RoB tool or checklist for stepped-wedge designs.

Reviewer 3

Name Wogu, Adane

Affiliation Colorado School of Public Health, Department of Biostatistics and Informatics

Date 06-Jun-2025

COI **None**

Dear authors,

Thank you for the opportunity to review the manuscript titled “The role of recruitment bias in stepped-wedge cluster randomised controlled trials: a systematic review,” which presents a systematic review examining recruitment bias in stepped-wedge cluster randomized trials published in 2023.

This manuscript tackles a relevant and timely research question, as SW-CRTs have gained significant popularity in recent years, particularly in healthcare settings. The finding that approximately 42% of SW-CRTs demonstrated a high risk of recruitment bias is concerning and warrants attention.

While it doesn’t violate any merit, my biggest concern on this manuscript is restricting the search to a single year (2023), which [in my opinion] significantly limits the generalizability of findings and may not capture the full spectrum of recruitment bias patterns in SW-CRTs.

Another point is that the exclusive use of two subdomains from the Cochrane RoB 2.0 tool may not fully capture the complexity of bias assessment in SW-CRTs.

Finally, the discussion section requires substantial revision to improve clarity, strengthen arguments, and better integrate findings with existing literature.

I appreciate the authors’ efforts in addressing an important topic, and, in the pdfi file of the manuscript itself (attached), I have provided a detailed review that I hope will be helpful in strengthening the manuscript. My comments reflect areas where clarification or further development may be needed, particularly in terms of data analysis and relevant discussions.

Please don’t hesitate to reach out if any clarification is needed regarding my review.

Sincerely,

**** **The reviewer provided a marked copy with additional comments. Please contact the publisher for full details.** ****

VERSION 1 - AUTHOR RESPONSE

	**Reviewer 1:** Lack of Depth Despite Narrow Scope:<br><br>The restriction of this review to only two subdomains of a single RoB domain naturally invites a more detailed treatment of the methodological issues at hand. However, the analysis presented is relatively superficial.	Thank you for your comments and giving us a chance to elaborate further on our paper.<br><br>Firstly, we have expanded the Methods section to include information on how the risk of bias assessment was performed, and on what factors our justifications were based for Domain 1 (separately for each included subdomain and signalling question) (please see the responses to individual domains below).

	For instance, in Domain 1a:<br>• While it is noted that random allocation was confirmed in 62 out of 64 studies, there is no systematic breakdown of the method of randomisation (e.g. simple, stratified, constrained), nor is there clarity on how this was verified.	Domain 1a:<br>• We have now expanded further on the results by including the method of randomisation. We also have added further details in the methods section to explain how the allocation concealment was judged.
Lines 149-163		In the Methods section:<br>*The following data were extracted from all included studies:1) general study information (title, country in which the study was conducted, authors, year, journal), 2) intervention characteristics (type and description of the intervention, study settings), 3) population characteristics (method of participants' recruitment and its description, age group (adults and/or children)) and 4) quality assessment (RoB) (Supplementary Material File 3, Table S2).* ***As part of quality evaluation, we extracted information about the type of randomisation (signalling question 1a.1) (e.g., simple, blocked, stratified)*** *and whether experienced independent personnel (e.g., a statistician) was responsible for randomisation sequence generation (signalling questions 1a.1 and 1a.2). We also looked for any mitigation strategies to avoid bias arising from identification and recruitment of participants in clusters (signalling questions 1b.1 and 1b.2), such as use of waiver of consent or retrospective medical records. To assess signalling question 1a.3 (individual-level baseline imbalances), we judged whether authors adjusted their analysis models for imbalanced baseline covariates. Such adjustments could have been either specified a priori, for all clinically meaningful covariates, or added in post-hoc analysis.*
Lines 188-189		*We ascertained random allocation if the term 'random' was mentioned in sequence generation.*
302-303		In the Results section:<br>*Where reported, restricted randomisation was commonly used (n=11, 17%); simple (n=1, 1.5%) and matched-pair (n=1, 1.5%) were also reported.*
Lines 339-341		In the Discussion section:<br>*When reported, stratification was used for randomisation to ensure the better balance of baseline characteristics between cluster-level and individual-level characteristics.*
	• Similarly, allocation concealment is discussed as being adequate in 75% of cases, but there is no explanation of how this judgement was reached. Did the authors report use of centralised randomisation or sealed envelopes? This detail is essential in a review focused on bias.	• We have added further details in the methods section to explain how the allocation concealment was judged. Also, we have expanded the Discussion section to address issues related to allocation concealment.
Lines 189-193		In the Methods section:<br>*Allocation was considered concealed when the sequence was controlled by personnel independent to the enrolment process. Due to the nature of SW-CRTs, all clusters eventually reach the intervention stage; therefore, in cases when all clusters became aware of*

		the allocation sequence at the beginning of the trial, we decided that allocation sequence was not concealed.
Lines 354-371		In the Discussion section:<br>*Allocation concealment (signalling question 1a.2) was underreported in four papers where no valuable information to address this element could be extracted [25,26,28,73]. In one paper [38], allocation sequence could be not concealed because the randomisation and recruitment were performed by one individual, although it is generally expected that enrolment is performed by an independent person. Moreover, in several trials with cross-sectional design (and therefore continuous recruitment), sites were informed of their allocated sequence before the enrolment [53,62,66,78], and unmasked recruiting staff were likely to contribute to differential identification and recruitment of potential participants. Shete et al [62] reported that the sequence order was announced to all sites during the stakeholder-led randomisation ceremony with health centre representatives choosing a numbered ball with their sequence order from an opaque bag. Since revealed allocation sequence with continuous recruitment design are likely to lead to potential selectivity when enrolling new participants, this paper had a high RoB. Additionally, the use of envelopes containing allocation information was reported in three papers [51,71,79]. Although the use of sealed envelopes is more likely to lead to the subversion in allocation concealment than a centralised computer system [82], we considered the use of opaque sealed envelopes (as reported in the trial) as concealed allocation [51], while in one trial [71] no information regarding whether the envelopes were sealed, opaque, or sequentially numbered was given, leading to the higher risk of bias for this signalling question.*
Lines 90-95<br><br>Lines 199-200	In Domain 1b:<br>• The authors report that 75% of studies recruited participants after randomisation, and that around one-third had evidence of selective recruitment. However, the operational definitions of "selective recruitment" and "differential identification" are vague.	The term 'differential identification and recruitment' has been used in Cochrane risk of bias tool 2.0 for cluster trials, therefore we have renamed 'selective recruitment' to 'differential recruitment' in line with this (previously we used 'selective' and 'differential' interchangeably).<br><br>We have added a paragraph to the Methods section, to define these terms:<br><br>*Selection bias in this review is defined as issues arising from differential identification and recruitment of potential participants in clusters, for example, if recruiting staff and/or potential participants are aware of allocation status. Such awareness is likely (either consciously or subconsciously) to affect the recruitment process [13] (e.g., recruit healthier patients in the control phase, and patients with multiple comorbidities during the intervention condition).*<br><br>*Subdomain 1b is a marker for differential identification and recruitment of participants (during screening and enrolment in clusters), and exclusive for cluster trials.*
	• It is unclear how masking of recruitment staff was determined, or whether this was based on explicit statements, indirect inference, or assumptions. Given the centrality of this point to Domain 1b, the	We have added further details in the methods section to explain how the masking of recruiting staff (and/or potential participants) was judged.<br><br>In the Methods section:

Lines 218-226	lack of transparency here undermines the strength of the RoB judgements.	*When assessing the possibility of differential recruitment, we focused on whether the recruiting personnel and potential participants were aware of the allocation sequence, and in cases when allocation sequence was revealed in advance, we answered 'Yes' / 'Possibly yes' (depending on whether explicit information was provided). In cases, when direct mentioning of masking of recruiting team was missing, we took into account whether the allocation sequence was revealed to a site trial team in advance and whether it was possible to mask recruiting staff to the implemented intervention (e.g., the research team could actually see that the intervention had been rolled out, for example, a new hospital facility), and answered 'Possibly yes' / 'Possibly no' / 'No information'.*
Lines 453-457<br><br>Lines 361-365	Underuse of Study-Level Examples:<br><br>The review would be considerably strengthened by highlighting examples of both good and poor practice. For instance:<br><br>• In the paper by Irvine et al. (ref. 34), the authors restricted the enrolment window for each implementation period, allowing sufficient follow-up time while limiting the risk of time-related confounding. This is a pragmatic strategy worth highlighting as good practice.<br>• Conversely, in Gyedu et al. (refs. 31, 32), substantial baseline imbalances in both comorbidity and hospital admissions are reported. The review mentions these issues briefly but does not evaluate whether these were adjusted for or addressed analytically. Nor is there sufficient discussion of whether such imbalances reflect differential recruitment, structural design weaknesses, or both.<br><br>Without such illustrations, the risk of bias assessment remains abstract and potentially unconvincing to a critical reader.	Thank you for suggesting this, we have included your suggested examples in the paper. We have also included more examples from the papers to the Discussion section to highlight the role of mitigation strategies in tackling the risk of bias.<br><br>*In the paper by Irvine with colleagues [36], the authors restricted the enrolment window for each implementation period, permitting more outcome observation time for each participant before the next step and limiting the risk of time-related confounding. This was a pragmatic strategy and example of a good practice.*<br><br>*Shete et al [62] reported that the sequence order was announced to all sites during the stakeholder-led randomisation ceremony with health centre representatives choosing a numbered ball with their sequence order from an opaque bag. Since revealed allocation sequence with continuous recruitment design are likely to lead to potential selectivity when enrolling new participants, this paper had a high RoB.*
Lines 391-397<br><br>Lines 383-386		*It is worth noting that the use of a waived informed consent could be an effective mitigation strategy only if inclusion criteria for participation are based on objective measures, e.g. age [31,35,43–46,52,68], diagnosis [45,49,68], laboratory values [45], or number of visits [35,68]. However, where a subjective measure is used, such as expected length of stay in healthcare facility (generally based on clinical judgement rather than objective tests) [22,23], the use of a waived informed consent cannot eliminate the risk of differential recruitment.*<br><br>*Schafthuizen et al [60] also mentioned that referral to gatekeepers' assistance for recruitment processes also may violate the unbiased approach to treatment, especially when gatekeepers are aware of the sequence allocation and recruitment is continuous.*
Lines 398-420	Overreliance on Baseline Imbalance as a Proxy:<br><br>The discussion often implies that baseline imbalance signals recruitment bias. While this may be true in some cases, it conflates a symptom with a mechanism. Imbalances can arise due to a range of factors, including small cluster numbers,	Thank you for your comment. We have added several points to the Discussion check to address issues related to baseline imbalances.<br><br>*Imbalanced baseline characteristics might be used as an indicator of recruitment bias. The review by Bolzern et al. [11] found that the risk of recruitment bias was much higher for cluster trials compared to individually randomised trials, particularly in terms of a baseline*

	time-varying site characteristics, or inadequate randomisation methods. Unless tied directly to unmasked recruitment or inappropriate allocation procedures, imbalance alone does not confirm a flaw in Domain 1a or 1b.<br><br>The review would benefit from explicitly distinguishing between:<br><br>• Structural/design-driven imbalances (e.g. seasonality, site variation),<br>• Procedural flaws (e.g. unmasked recruitment, foreknowledge of allocation),<br>• Chance variation due to underpowered designs.<br><br>This nuance is currently missing.	*characteristic such as age. The authors compared age imbalances in cluster and individual RCTs in meta-analyses and revealed that while in all individually randomised trials age was balanced between intervention groups, in 14 out of 23 cluster trials (61%) age imbalances were identified. In our review, a number of the included studies had individual-level covariate imbalance in baseline characteristics (n = 40, 63%) particularly in clinically important variables (e.g., number of comorbidities and severity of disease [17,28,30,54,57] number of hospital admissions or length of stay in hospital [28,54,77]). Generally, the number of participants per cluster was well-reported; however, the assessment of inter-cluster differences in participants' characteristics was problematic as only a few papers (n = 10, 16%) included data by cluster. Shields et al [63] and Krauss et al [41] reported participants characteristics by clusters but not a comparison between intervention and control conditions; Yang et al [77] and Leal et al [43] reported characteristics separately by study step and allocation; and in four papers [27,29,30,37] data were reported by allocation and clusters. Inclusion of baseline characteristics in statistical analyses as covariates in adjusted regression models in either primary or sensitivity analyses was a method commonly used in included trials to mitigate potential baseline imbalances [17,22 –25,29–31,33,35,40,41,46,47,49,53,56,61–63,67,75,78]. In a few cases, adjustments for models were not specified a priori: several authors [22,53,63] acknowledged the need for post-hoc adjustment for imbalanced baseline characteristics. Importantly, these imbalances can be symptomatic of other issues such as small cluster numbers, time-varying site characteristics, or inadequate randomisation methods.*
Lines 426-440		*Importantly, imbalances in baseline characteristics do not necessarily indicate problems with randomisation or recruitment, and their sole presence might not necessarily lead to a high risk of bias. Among the trials with identified baseline imbalances in either subdomain 1a or 1b (n=39, 61%), 20 trials demonstrated signs of differential recruitment, but only in 7 of them the authors associated imbalances with recruitment performed by unmasked research team [22,38,42,43,54,59,66]. In some trials (n=9) the authors acknowledged that recruitment was below the recruitment target, leading to underpowered results [22,26,38,56,57,59,60,71,77]. In the majority of these cases (n=14 out of 20), imbalance problems were linked to variations in site demographics and processes [27,30,33,46,49,50,60–62,67,71] or site retention [37,38,55]. In one paper [25] the rapid increase in the number of eligible participants was associated with the change of electronic health records provider. Another major reason for imbalances was COVID-19 pandemic, causing early termination or halt of some trials [17,23,31,32,41,56,73,74,76], withdrawals of clusters [28,37], and restructuring of wards [17,37], which affected the balance between allocation sequences, and the comparability of clusters.*

Lines 441-457		*Although some of these imbalances might have been due to chance, the risk of recruitment bias could not be ruled out completely. Importantly, regardless of the study design (cross-sectional or open cohort), time-related (e.g., seasonal) imbalances in site-level covariates might be expected [84]. Some of these time-related factors are usually uncontrollable and/or unavoidable (e.g., general policy changes, staff turnover, seasonal changes such as different hospital load in winter and summer, different infection rates throughout the year [46,85]), whereas other factors (e.g., staff rotation or recruitment performed by unmasked team members) can be avoided. Hypothetically, in cases when the potential risk of any time-related changes on site level required mitigation actions, implementation of fewer cluster sequences with greater number of clusters per sequence might be more effective because trial duration (and consequently the risk of any uncontrollable changes) will be minimised. However, it might cause logistical and financial problems. Time constraints might also negatively affect outcome measures as for some interventions their effect could be delayed. In the paper by Irvine with colleagues [36], the authors restricted the enrolment window for each implementation period, permitting more outcome observation time for each participant before the next step and limiting the risk of time-related confounding. This was a pragmatic strategy and an example of good practice.*
 <br><br><br><br>Lines 280-282<br><br><br>Lines 287-289<br><br><br><br>Lines 310-315	Missed Opportunity for Thematic Synthesis:<br><br>Beyond reporting frequencies, the review does little to extract cross-cutting themes from the data. For example:<br><br>• How often did studies mitigate risks through automatic enrolment, waiver of consent, or analytic adjustment?<br>• Was there a pattern in risk of bias by study setting, region, or type of intervention?<br>• Were certain journals or trialists more consistent in reporting allocation methods?<br><br>A simple table listing mitigation strategies by study would offer far more value than the current results section provides.	We thank the reviewer for these further suggested items and have included most of them in Table 1.<br><br>As there tended to be only one included paper per journal, we added an explanation of this to the results:<br><br>*We planned to summarise data by journals, but it was not feasible as usually there was only one trial per journal.*<br><br>Please see the updated Table 1 (by Risk of bias).<br><br>Also, we could not produce a simple table with mitigation strategies as the strategies were quite varied, and the table was uninformative. We added the narrative description of the mitigation strategies to the Result and Discussion sections:<br><br>*The mitigation strategies to tackle potential selection bias included:*<br>*1) inclusion of all eligible participants during recruitment [30,61,64,69,79], however, using this strategy still required seeking participants' informed consent.*<br>*2) inclusion of retrospective medical records for all eligible patients, using such a strategy did not require informed consent to be sought [29,37,39]; or*<br>*3) the use of waived consent [22,23,28,31,35,43–46,49,52,68].*

Lines 387-397		*In order to minimise any potential selectivity while recruiting continuously in open cohort and cross-sectional designs (signalling question 1b.2), the authors used different mitigation strategies by including all eligible participants, using a consent waiver, or using the medical records of all eligible patients. These strategies allow the inclusion of all or almost all patients, therefore minimising the role of selectivity in recruitment. It is worth noting that the use of a waived informed consent could be an effective mitigation strategy only if inclusion criteria for participation are based on objective measures, e.g. age [31,35,43–46,52,68], diagnosis [45,49,68], laboratory values [45], or number of visits [35,68]. However, where a subjective measure is used, such as expected length of stay in healthcare facility (generally based on clinical judgement rather than objective tests) [22,23], the use of a waived informed consent cannot eliminate the risk of differential recruitment.*
Lines 475-540	Weak and Inconclusive Final Discussion<br><br>Given the findings—particularly that over 40% of trials were at high risk of recruitment or randomisation bias—the final conclusion feels underpowered. The claim that “some bias is unavoidable” is true, but it lacks critical edge. The review does not draw a line between inherent design constraints and avoidable methodological weaknesses. Nor does it offer concrete recommendations to funders, journal editors, or ethics committees on improving the quality of SW-CRT design and reporting.<br><br>Consider using this opportunity to advocate for:<br><br>• Improved pre-registration of randomisation and recruitment protocols,<br>• Mandatory reporting of allocation concealment methods in SW-CRTs,<br>• Development of a tailored RoB tool or checklist for stepped-wedge designs.	We have expanded the recommendation sections and provided reasoning for such recommendations.<br><br>Please refer to subsection Recommendation (as part of the Discussion section).
	**Reviewer 2**	
Lines 87-96	One of my primary concerns is that the authors are not very clear about their definitions of biases and their sources. For example, I am unclear by what the authors mean when they refer to “differential recruitment of participants” or “differential randomization processes” (page 2, paragraph 2) so I cannot assess the mechanisms by which they would contribute to recruitment bias. In addition, when the authors say “recruitment bias”, it is unclear whether they mean bias due to self-selection of the participants or recruitment tactics by the study team. While both may be categorized under the broad umbrella of “recruitment bias”, I’d argue that they are	Thank you for your comments, they were very helpful and encourages us to provide a more in-depth analysis of the results and elaborate on the methods.<br><br>We have renamed ‘selective recruitment’ to ‘differential recruitment’ in line with ‘differential identifications’ (previously we used ‘selective’ and ‘differential’ interchangeably). The term ‘differential identification and recruitment’ has been used in Cochrane risk of bias tool 2.0 for cluster trials, so we have added a paragraph to the Methods section, to define and differential the terms. We have also included description of ‘recruitment bias’ and ‘selection bias’ as they are being used in this review.<br><br>*In this review, we use terms ‘recruitment bias’ and ‘selection bias’ as per the following descriptions. Recruitment bias in this review is a broader term,*

Lines 199-200	two distinct sources with quite different mitigation strategies. I would appreciate if the authors would clarify these points, as I think it will make the narrative of the manuscript and the aims of the review more clear.	*addressing any issues arising due to randomisation process, allocation concealment, recruitment process and masking of recruiting staff and potential participants. Selection bias in this review is defined as issues arising from differential identification and recruitment of potential participants in clusters, for example, if recruiting staff and/or potential participants are aware of allocation status. Such awareness is likely (either consciously or subconsciously) to affect the recruitment process [13] (e.g., recruit healthier patients in the control condition, and patients with multiple comorbidities during the intervention condition). Therefore, in this review selection bias is deemed one of the components of recruitment bias.*<br><br>*Subdomain 1b is a marker for differential identification and recruitment of participants (during screening and enrolment in clusters), and exclusive for cluster trials.*
Lines 73-76	The authors should make more clear somewhere in the manuscript that unrestricted randomization in CRTs only guarantees balance of cluster-level baseline characteristics, and does not guarantee balance of individual-level characteristics.<br>3 (line 7)	Thank you for the comment, the note has been added to the Introduction sections:<br><br>*It is worth mentioning that simple (unrestricted) randomisation can guarantee balance on baseline characteristics only on the cluster level, but not on the individual one, particularly when sample size is small; the balancing characteristics on individual level would require the use of restricted randomisation [7].*
Lines 54-58	"more sources of bias" – you've not yet mentioned any potential sources or risks of bias in SW-CRTs. I would consider outlining these first before moving into bias due to recruitment.<br><br>Page 2 (line 46) – original pdf document	We have reworded the sentence:<br><br>*Since the outcomes of these trials affect the direction and implementation of healthcare standards, the results of such trials should be robust and unbiased; however, the complex study design and often continuous recruitment during both study conditions can lead to* ***various sources*** *of bias, such as, awareness of the recruitment team of the allocation status and therefore selective recruitment of participants.*
Lines 237-239	The authors should clarify what they mean by "study setting", and how they may different from the definition of the unit of randomization.<br><br>Page 4 (line 30)) – original pdf document	We have added clarification to the Methods section:<br><br>*3) study setting – organisational units (e.g., hospitals, schools, primary care facilities) which were not necessarily used as the unit of randomisation (e.g., hospital wards within a hospital).*
Line 245	I would suggest the authors use some other phrase for the total RoB in this review than "overall RoB" if they are only tallying it over Domain 1 – perhaps "overall Domain 1 RoB".<br><br>Page 5 (lines 3-4) – original pdf document	It was renamed to *'Domain 1 overall RoB'*
Lines 176-183	what is the difference between "low risk", "high risk", and "some concerns". Is "some concerns" lower risk than "low risk"? Between low and high risk? In addition, why are "high risk" and "some concerns" grouped together in Figure 2?<br><br>Page 5 (lines 23-24) – original pdf document	Thank you for your comment. Additional clarifications have been added to the paper in the Methods section:<br>*RoB reporting includes a traffic-light system, where 'low risk' shows minimal concerns regarding sources of bias, 'some concerns' indicates one or two moderate issue or several minor ones, and 'high risk' signals major issues or the combination of moderate ones). When assessing each signalling questions, we responded 'Yes' / 'No', when clear information was provided, and 'Possibly yes' / 'Possibly no' when reported information is limited but*

Figure 2		*implies a certain answer. In cases where no information was provided, we selected the option 'No information' (for detailed information on RoB algorithms refer to Supplementary Material File 4, Table S1 and Table S2).*<br><br>For Figure 2, the idea was to present separately responses indicating any risk of bias versus responses indicating no risk of bias. We have relabelled the response options for Figure 2:<br>– A score of "High risk" or "Some concerns" was considered as an indicator of potential "risk of bias".<br>– A score of "Low risk" was indicative of the study not posing a risk of bias<br>– No information to allow assessment of risk of bias<br>– Not applicable (a default option for Selection bias signalling question 1b.2 in cases when recruitment was conducted prior to the randomisation)
Line 277	What do the authors mean by "random tiers"?<br><br>Page 6 (line 8) – original pdf document	Corrected in the manuscript:<br><br>*Special needs school*
Lines 184-189	I would not say that no problems were identified with the random allocation process. I believe what question 1a.1 gets at is whether random allocation happened. There may still issues with that process even though random allocation did occur.<br><br>Page 7 (lines 39-40) – original pdf document	Thank you for your comment. We have added explanations to the Methods section on how we approached assessment of individual singling questions:<br><br>*Both subdomains 1a and 1b are required for the assessment of recruitment bias. Subdomain 1a is an indicator of problems with the randomisation process. It includes assessment of whether randomisation was performed (signalling questions 1a.1), the allocation sequence was concealed (signalling question 1a.2), and if any baseline differences of individual participants between intervention groups resulted by chance (signalling question 1a.3). We ascertained random allocation if the term 'random' was mentioned in sequence generation.*
Lines 193-198<br><br><br><br><br><br>Lines 226-230	Do the authors mean baseline differences in cluster-level characteristics or in individual-level characteristics?<br><br>Page 7 (line 42) – original pdf document	Thank you for your comment. We referred to baseline differences on individual level when assessing data between allocation and control conditions for signalling question 1a.3, and for inter-cluster differences when assessing signalling question 1b.3. We have added explanations to the Methods and Results sections.<br><br><u>In the Methods section:</u><br>*When assessing the baseline differences between intervention conditions, we evaluated data from baseline tables with sociodemographic and important clinical variables, comparing the conditions either with proportions or means and standard deviations (or more suitable summary statistics) where provided or with p-values when available. We have not compared differences between clusters in this subdomain.*<br><br>*When assessing inter-cluster differences, we evaluated the imbalances in baseline characteristics between clusters (where reported) and the number of participants per cluster. Imbalances in baseline characteristics were assessed by comparing proportions, means and standard deviations as appropriate, or p-values where available.*

Lines 299-319		In the Results section:<br>*Results of Subdomain 1a revealed that 63 studies (98%) had clearly described the random allocation process whereas only 48 studies (75%) reported allocation concealment, see Figure 2.*<br>*Where reported, restricted randomisation was commonly used (n=11, 17%); simple (n=1, 1.5%) and matched-pair (n=1, 1.5%) were also reported. In one third of all included studies (n=24, 38%) the authors mentioned a statistician was involved in the randomisation process.*<br>***Individual-level baseline differences were identified in 25 (39%) studies.***<br>*Analysis of subdomain 1b revealed that the majority of the included studies recruited participants after randomisation (n=52; 81%). Just under 60% of included studies (n=30 out of 52) were prone to selective recruitment of participants; these are studies where recruiting staff were not masked to the allocation status or no sufficient information was available to assess the question. The mitigation strategies to tackle potential selection bias included:*<br>*1) inclusion of all eligible participants during recruitment [30,61,64,69,79], however, using this strategy still required seeking participants' informed consent.*<br>*2) inclusion of retrospective medical records for all eligible patients, using such a strategy did not require informed consent to be sought [29,37,39]; or*<br>*3) the use of waived consent [22,23,28,31,35,43–46,49,52,68].*<br>***In 60 (94 %) manuscripts, individual-level baseline characteristics by intervention conditions were presented****. Of these, 40 (67%) suggested differential identification and/or differential recruitment of individuals to clusters. In 23 manuscripts (36%), the authors adjusted statistical models to imbalanced covariates.*
Lines 208-211	"More than a half of the papers (n = 40 out of 60 papers where baseline characteristics between intervention groups were presented, 67%) suggested differential identification and/or recruitment of individuals in clusters." I'm unclear of what the authors mean by "differential identification and/or recruitment of individuals in clusters"<br><br>Page 7 (lines 48-51) – original pdf document	Thank you for your comment. Explanation was added to the manuscript:<br>'*Differential identification and/or recruitment of individuals in clusters' refers to the problems identified with signalling question 1b.2. Differential identification refers to the identification and screening process, and differential recruitment refers to the actual recruitment (often by research teams unmasked to allocation status).*
Lines 380-397	If the authors collected specific data on the masking of recruitment team members, I think that would be very useful to present. As it currently stands, it's difficult to disentangle the sources of recruitment bias in the trials reviewed – are the majority of trials at risk because	Thank you so much for your comment, we added how often unmasked recruitment was mentioned in papers, and what mitigation strategies were used to reduce the risk of bias:<br><br>*In cases when recruitment is ongoing and/or allocation sequence is revealed to all sites before the recruitment*

	randomization happens prior to recruitment? How common is masking of the recruitment teams?<br><br>Page 8 (lines 56-60) – original pdf document	*starts, it is crucial to mask recruiting staff to avoid selection bias (signalling question 1b.2). The details of recruitment process and staff masking were generally underreported (n=7, 11%) or staff was unmasked (n = 23 trials, 36%). Schafthuizen et al [60] also mentioned that referral to gatekeepers' assistance for recruitment processes also may violate the unbiased approach to treatment, especially when gatekeepers are aware of the sequence allocation and recruitment is continuous.*<br><br>*In order to minimise any potential selectivity while recruiting continuously in open cohort and cross-sectional designs (signalling question 1b.2), the authors used different mitigation strategies by including all eligible participants, using a consent waiver, or using the medical records of all eligible patients. These strategies allow the inclusion of all or almost all patients, therefore minimising the role of selectivity in recruitment. It is worth noting that the use of a waived informed consent could be an effective mitigation strategy only if inclusion criteria for participation are based on objective measures, e.g. age [31,35,43–46,52,68], diagnosis [45,49,68], laboratory values [45], or number of visits [35,68]. However, where a subjective measure is used, such as expected length of stay in healthcare facility (generally based on clinical judgement rather than objective tests) [22,23], the use of a waived informed consent cannot eliminate the risk of differential recruitment.*
	**Reviewer 3:**	
Lines 101-111	While it doesn't violate any merit, my biggest concern on this manuscript is restricting the search to a single year (2023), which [in my opinion] significantly limits the generalizability of findings and may not capture the full spectrum of recruitment bias patterns in SW-CRTs.	Thank you for your comments and your concerns. They were extremely helpful in shaping our paper and providing additional clarifications and justifications.<br><br>Although the initial idea for this review was not to limit the search results to any particular publication year, the annually increasing number of published stepped-wedge reviews made this wider inclusion criteria impractical and less feasible (particularly as the review was not funded research and had limited personnel).<br><br>The limitation to the one most recent completed calendar year (at the moment of submission) was guided by the necessity to provide a 'snapshot' of current practices and standards in reporting the results of stepped-wedge cluster randomised controlled trials with focus on recruitment bias.<br><br>Also, it was expected that the quality of reporting and mechanism of addressing potential sources of recruitment bias in trials should continuously improve (as more and more authors follow closely the CONSORT reporting guidelines), the most recent calendar year has also been chosen as potentially an example of current best practices in addressing recruitment bias issues and higher research transparency.<br><br>We have added additional explanations to the Methods and Discussion sections.<br><u>In the Methods section:</u><br>*As it was anticipated that the quality of reporting trials continues to improve over time [2,14], the publication*

Lines 459-464		*date was limited to the calendar year 2023 due to the significant increase in annual number of registered stepped-wedge trials since Hussey and Hughes paper in 2007 [15]. Based on a PubMed database search [5], since 2021 over two hundred newly published papers that use the design were added annually. Therefore, we focused our review on the calendar year 2023 (the most recent complete calendar year at the time of data retrieval). Furthermore, it was assumed that articles published in that year would potentially be conducted and reported to higher standards than earlier years due to the increased awareness and use of the CONSORT statements partly driven by high impact journals consistently insisting on the reporting of randomised controlled trials to these guidelines.*<br><br>In the Discussion section:<br>*This review has several limitations. The search strategy was limited to only one year (2023), and although expected to demonstrate the most current practices in addressing the risk of recruitment bias, it also violates generalisability of the review results. Importantly, the choice of 2023 implied taking into consideration direct effects or delayed consequences of the recent coronavirus pandemic (COVID-19), which might have affected recruitment process [86]; however, in this review no adjustments for the recent pandemic were made.*
Lines 165-172	Another point is that the exclusive use of two subdomains from the Cochrane RoB 2.0 tool may not fully capture the complexity of bias assessment in SW-CRTs.	Thank you for your comment. It is definitely true that measuring all five domains would provide potentially a more detailed quality assessment. However, our review focuses only on sources of recruitment bias and strategies of its mitigation, therefore we used only Domain 1 as it is the only domain that would be fundamental for recruitment bias assessment. The use of Domain 1 exclusively was intentionally driven by decision to assess risks associated with the randomisation process itself (Domain 1a) and additional risk associated with using a cluster design (Domain 1b). We have also added this justification to the paper:<br><br>*The current version 2.0 of the Cochrane Collaboration tool for assessing the risk of bias (RoB 2.0) for cluster RCTs [13] was used. The tool incorporates five domains. Domain 1 includes assessment of bias arising from randomisation process and identification and recruitment of participants in a cluster RCT and was selected for this review for assessment of recruitment bias. The other four domains assess other sources of bias (deviation from intended interventions, missing data, outcome measurement, and selective result reporting) and do not contribute directly to the assessment of recruitment bias; these were not evaluated within the current review since our focus was limited to recruitment bias.*
Lines 320-540	Finally, the discussion section requires substantial revision to improve clarity, strengthen arguments, and better integrate findings with existing literature.	Thank you for your suggestion, we provided additional justifications and recommendations in the Discussion section.<br><br>Please refer to the Discussion section

Line 64	Do you mean SW-CRT? Otherwise spell it out please.<br><br>Page 2 – original pdf document	Corrected in the manuscript:<br><br>*stepped-wedge cluster design*
Lines 82-86	This systematic review was conducted in accordance with established methodological principles and reported following the Preferred Reporting Items for Systematic Reviews and Meta-Analyses (PRISMA) guidelines [12]. The complete PRISMA checklist is provided in Supplementary Material File 1. No public or patient involvement was included in this review.<br><br>Page 3 – original pdf document	Thank you for the suggested changes to the wording, we have made these changes to the manuscript:<br><br>*This systematic review was conducted in accordance with established methodological principles and reported following the Preferred Reporting Items for Systematic Reviews and Meta-Analyses (PRISMA) guidelines [12]. The complete PRISMA checklist is provided in Supplementary Material File 1. The review uses published results of SW-CRTs, therefore no ethical approval was required.*
Lines 120-125	Could you be more specific about grey literature?<br><br>While "unpublished sources," was mentioned, it doesn't specify what grey literature databases or sources were actually searched (e.g., conference proceedings, theses, clinical trial registries)<br><br>Page 3 – original pdf document	Although it is expected that all included trials will be peer-reviewed, there was no exclusion criteria for the ones without a peer-review. Therefore, several included papers identified with the search strategy (n=6) were preprints.<br><br>We made changes to the manuscript:<br><br>*Searches for published materials (both peer-reviewed and pre-prints) were conducted in four electronic databases (MEDLINE (Ovid), Embase (Ovid), Cochrane Library (Wiley, 2024, Issue 1), and CINAHL Ultimate (EBSCO)). The search strategy for literature searches electronic databases was developed with use of medical subject headings initially for MEDLINE and later adapted for other databases (Supplementary Material File 2). All papers meeting the inclusion criteria (SW-CRT design and 2023 publication year) were included.*
Lines 120-122	"sources" and "materials" coming together made the statement awkward and redundant.<br><br>Page 3 – original pdf document	We have reworded the sentence to avoid confusion:<br><br>*Searches for published materials (both peer-reviewed and pre-prints) were conducted in four electronic databases (MEDLINE (Ovid), Embase (Ovid), Cochrane Library (Wiley, 2024, Issue 1), and CINAHL Ultimate (EBSCO)).*
Lines 143-146	Grammar correction<br><br>Page 4 (line 23) – original pdf document	Thank you for spotting this, the change has been made:<br><br>*A calibration exercise with two included studies took place before the extraction process to confirm the legibility of extraction form and ensure that both reviewers extract data in the same way and deal similarly with ~~the~~ missing data.*
Lines 149-153	This paragraph needs some improvements for clarity and methodological rigor.<br><br>Some points:<br>1. The section uses "statistical analysis" as the heading but describes mostly descriptive data management rather than statistical analysis. Consider "Data	Thank you for your comment. The paragraphs have been re-written to incorporate explanation of the categories:<br><br>*The following data were extracted from all included studies:1) general study information (title, country in which the study was conducted, authors, year, journal), 2) intervention characteristics (type and description of the intervention, study settings) and 3) population characteristics (method of participants' recruitment and*

Lines 231-243	Analysis and Synthesis" or "Data Management and Analysis."<br>2. The ad hoc approach to age grouping is problematic. The statement "no specific guidelines for age limits for the review were followed" suggests inconsistent methodology. This needs justification or standardization.<br>3. "Combined into informative categories where possible" is too vague. The criteria and process for categorization should be specified.<br><br>[The paragraph has or (it conveys) the intended methods, but it has several issues related to clarity, structure, grammar, and consistency with standard systematic review reporting.]<br><br>Page 5 – original pdf document	*its description, age group (adults and/or children)) (Supplementary Material File 3, Table S2).*<br><br>***Data management and analysis***<br>*Data extracted in Covidence were exported to Microsoft Excel for cleaning and summarising. Extracted categorical data were categorised into: 1) geographic region (based on World Bank classification (www.data.worldbank.org) (Europe, South Asia, East Asia and Pacific, Middle East, North America, Latin America and Caribbean, Africa, or multiple regions); 2) population – based on participants' age (over 18 years old – adults, under 18 years old – children and adolescents); 3) study setting – organisational units (e.g., hospitals, schools, primary care facilities) which were not necessarily used as the unit of randomisation (e.g., hospital wards within a hospital); 4) type of intervention (e.g., educational, screening tool) – the decision based on how the authors of the original paper described their intervention. The data were presented as frequencies and percentages. The data were presented overall and by risk of bias category (high, some concerns, low). The differences between RoB groups were described narratively, without formal statistical testing due to a small number of studies per group.*
Line 232	Wording of:<br>*After being collected in Covidence, the data were exported to Microsoft Excel Spreadsheet for further cleaning and summarising.*<br><br>Page 5 – original pdf document	Thank you for suggesting this, the change has been made:<br><br>*Data extracted in Covidence were exported to Microsoft Excel for cleaning and summarising.*
Line 254	Formatting<br><br>Page 5 – original pdf document	Additional 'paragraph' sign between words 'see' and 'Figure 1' has been removed.
	~~I~~ will put the "strength" of the Discussion Section here.<br><br>Strength<br>The discussion follows logical progression from presenting key findings to discussing implications, limitations, and future directions. The authors address multiple important aspects including recruitment bias patterns, mitigation strategies, comparisons with other study types, and practical recommendations. Especially, they draw relevant comparisons with other systematic reviews and appropriately reference limitations of comparing across studies due to different RoB tools. Further, they acknowledge several methodological limitations,	Thank for you very kind comment!

	including the single-year search strategy and language bias.<br><br>Page 7 – original pdf document	
Lines 323-324	Wording correction:<br>388 potentially relevant papers published in 2023 were screened, of which 64 met the inclusion criteria. Nearly half of these exhibited a high risk of recruitment bias.<br><br>Page 7 – original pdf document	We have reworded the paragraph:<br><br>*In this review, 388 potentially relevant papers published in 2023 were screened, of which 64 met the inclusion criteria. Nearly half of these exhibited a high risk of recruitment bias.*
Lines 426-457	This paragraph needs some reorganization and, most importantly, clarification on "causality".<br><br>The paragraph implies that imbalance always signals "s, which is not necessarily true (it could be random). Phrases like "usually demonstrated by poor balance" suggest causation without sufficient evidence. The connection between unmasked staff and covariate imbalance needs clearer substantiation or more cautious language.<br><br>Page 8 – original pdf document	Thank you for your suggestion, we provided additional justifications and recommendations in the Discussion section.<br><br>*Importantly, imbalances in baseline characteristics do not necessarily indicate problems with randomisation or recruitment, and their sole presence might not necessarily lead to a high risk of bias. Among the trials with identified baseline imbalances in either subdomain 1a or 1b (n=39, 61%), 20 trials demonstrated signs of differential recruitment, but only in 7 of them the authors associated imbalances with recruitment performed by unmasked research team [22,38,42,43,54,59,66]. In some trials (n=9) the authors acknowledged that recruitment was below the recruitment target, leading to underpowered results [22,26,38,56,57,59,60,71,77]. In the majority of these cases (n=14 out of 20), imbalance problems were linked to variations in site demographics and processes [27,30,33,46,49,50,60–62,67,71] or site retention [37,38,55]. In one paper [25] the rapid increase in the number of eligible participants was associated with the change of electronic health records provider. Another major reason for imbalances was COVID-19 pandemic, causing early termination or halt of some trials [17,23,31,32,41,56,73,74,76], withdrawals of clusters [28,37], and restructuring of wards [17,37], which affected the balance between allocation sequences, and the comparability of clusters.*<br><br>*Although some of these imbalances might have been due to chance, the risk of recruitment bias could not be ruled out completely. Importantly, regardless of the study design (cross-sectional or open cohort), time-related (e.g., seasonal) imbalances in site-level covariates might be expected [84]. Some of these time-related factors are usually uncontrollable and/or unavoidable (e.g., general policy changes, staff turnover, seasonal changes such as different hospital load in winter and summer, different infection rates throughout the year [46,85]), whereas other factors (e.g., staff rotation or recruitment performed by unmasked team members) can be avoided. Hypothetically, in cases when the potential risk of any time-related changes on site level required mitigation actions, implementation of fewer cluster sequences with greater number of clusters per sequence might be more effective because trial duration (and consequently the risk of any uncontrollable changes) will be minimised. However, it might cause logistical and financial problems. Time constraints might also negatively affect outcome measures as for some*

		interventions their effect could be delayed. In the paper by Irvine with colleagues [36], the authors restricted the enrolment window for each implementation period, permitting more outcome observation time for each participant before the next step and limiting the risk of time-related confounding. This was a pragmatic strategy and an example of good practice.
Lines 87-96	"Bias"<br><br>You occasionally switch between "selection bias" and "recruitment bias"-- these are related but not always interchangeable.<br><br>Page 8 – original pdf document	We added definition of the terms to the manuscript and rectified all inconsistencies in the text:<br><br>*In this review, we use terms 'recruitment bias' and 'selection bias' as per the following descriptions. Recruitment bias in this review is a broader term, addressing any issues arising due to randomisation process, allocation concealment, recruitment process and masking of recruiting staff and potential participants. Selection bias in this review is defined as issues arising from differential identification and recruitment of potential participants in clusters, for example, if recruiting staff and/or potential participants are aware of allocation status. Such awareness is likely, either consciously or subconsciously, to affect the recruitment process [13], for example, increased tendency to recruit healthier patients during the control phase in contrast to increased tendency to recruit patients with multiple comorbidities during the intervention phase. Therefore, in this review selection bias is deemed one of the components of recruitment bias.*
Lines 320-540	These three paragraphs, while informative, would benefit from clearer contextual paraphrasing or reorganization. There are two (or possibly three) distinct elements at play: a comparison with other reviews ("Comparison"), and a subsequent discussion on mitigations and analytic adjustments ("Recommendation"). While the "Comparison" part is generally sound (though with limited contextual framing) the "Recommendation" part is critically underdeveloped, despite its importance. The authors should expand on this section; for instance, by clarifying what constitutes "appropriate analysis" or specifying how masking should be implemented in practice.<br><br>Page 8 – original pdf document	Thank you for your suggestion, we provided additional justifications and recommendations in the Discussion section as per the suggested structure.<br><br>Please refer to the Discussion section
Lines 516-540	The critique of RoB 2.0 for SW-CRTs is valuable but could be expanded with specific recommendations for tool modification.<br><br>Page 9 – original pdf document	We have expanded the recommendation sections and provided reasoning for such recommendations.<br><br>Please refer to subsection Recommendation (as part of the Discussion chapter).<br><br>*Theoretically, a combination of the RoB tool for cluster trials [13] with the RoB tool for cross-over trials [89] would address some design challenges of SW-CRTs underlooked by the cluster RoB tool, including adjusting for a trial period. The RoB tool for cross-over trials include three signalling questions in the special Domain*

		S: confirmation of the equal number of participants per sequence (signalling question S1), inclusion of period-effects in the analysis in cases where there is an imbalance between sequences (signalling question S2), and appropriate duration of a transition period to allow any carry-over effects to disappear (signalling question S3). The suggested combination of these tools is given in Table 3.<br><br>*Such a tool would still demand high quality and transparency when reporting the details of the randomisation process (which is also in line with CONSORT guidelines). The role of recruitment before randomisation would be less significant; however, the role of masking of research staff involved in recruitment would still be essential. Also, the baseline comparison between sequences (rather than between clusters) would be more reasonable as more than one cluster can be part of a sequence. When only one cluster is randomised per sequence the baseline comparison between sequences will be equivalent to the comparison between clusters. Furthermore, temporal effects might be crucial in SW-CRTs to account for any trends over time (either external (e.g., seasonal changes) or internal (e.g., more active recruitment in the intervention phase)).Where possible, a transition period should be incorporated into the trial design to reduce any carryover effects, and allow for a smooth implementation of the intervention in the next step of the sequence.*<br><br>*Further methodological research is required to investigate the adherence SW-CRTs to CONSORT guidelines. The development of a new tool for risk of bias assessment of SW-CRTs is also highly desirable.*
Lines 252-254	419 and 388 do not add up to 808.<br><br>Page 17 – original pdf document	Thank you for checking this. There was a discrepancy between the number of papers and trials, Figure 1 has been updated, and narrative description has been changes:<br><br>*Overall, 808 study records (from 807 trials) published in 2023 were identified. After removal of duplicates, 388 titles and abstracts were screened, 144 full texts were examined, and 64 trials (reported in 65 papers) were ultimately included in the final review [16–80], see Figure 1.*
Suppl. file 5	Table S1 or Table 1?<br><br>Page 22-54 – original pdf document (Supplementary File 5)	Thank you for spotting this, Corrected to ***Table S1*** throughout the Supplementary File 5.

VERSION 2 - REVIEW

Reviewer 1

Name Ryan Baumann, Mary

Affiliation University of Wisconsin-Madison School of Medicine and Public Health, Population Health Sciences; Biostatistics and Medical Informatics

Date 03-Sep-2025

COI

Review of bmjopen-2024-096281.R1

I thank the authors for addressing many of my comments from the first review – the clarifications are much appreciated. I have several additional (mostly minor) suggestions and clarifications that I believe will improve the quality of the manuscript; I list them below in order of where they appear:

1. Throughout, I would appreciate that if the authors report a percentage, that they include a denominator along with their reported frequency. There are several places throughout the text where I tried to back-calculate the percentage based on the reported frequency and my assumption of the denominator, only to obtain a percentage that did not match with the one reported.

2. Page 5, lines 203-204: "It includes an assessment of whether the randomization of clusters was performed prior to or after randomization…" I think one of the randomizations in this sentence is a typo.

3. Page 8, lines 298-299: There is an internal reference error

4. Page 9, lines 307-309: The types of randomization scheme reported only account for 20% of those studies that reported their randomization allocation process; what types of randomization methods were used for the other 80% of studies?

5. Page 9, lines 311-312: I would be interested to see a breakdown of the sampling scheme (closed-cohort vs cross-sectional/open-cohort) of studies that recruited participants after randomization. I think this would allow us to see if most of these studies were prone to this bias by choice of design (such as in the case of a cross- sectional scheme), or if they were disregarding best practices (such as in the case of a closed-cohort design where clusters are randomized, but the cohort within the clusters is not recruited until after randomization occurs).

6. Page 9, lines 322-323: If possible, I would appreciate a breakdown in why these 40 studies had a suggestion of differential identification and/or differential recruitment of individuals to clusters. Was it solely based on differences in (individual-level) baseline characteristics? Differences in cluster sizes?

7. Page 10, lines 335-341: I'm unclear on where the percentages from the current review are coming from, as they don't line up with what is reported in Table 2 (they all seem to be off by +/- 2%).Page 11, lines 412-415: You report thar 40 studies had imbalance on baseline characteristics, but report on page 9 (line 310) that 25 studies were identified as having individual-level baseline differences. Are these two referring to different concepts? Could the 40 mentioned in the discussion be referring to the 40 studies on lines 322-323 that suggested differential identification and/or differential recruitment of individuals to clusters?

**Reviewer**	**2**
**Name**	**Kolokythas, Nico**
**Affiliation**	**University of Wolverhampton, Nico Kolokythas**
**Date**	**12-Aug-2025**
**COI**	

Thank you for responding to my comments. You have made several meaningful improvements since the last version, and it's worth acknowledging those first. The abstract now follows BMJ Open's required format, and you have included PPI and ethics statements in the Methods (Page 4, lines 131-136). The methods section is clearer in setting out what was extracted for Domain 1a and 1b, including the type of randomisation and how allocation concealment was judged. You have also fixed the earlier ambiguity by distinguishing individual-level from cluster-level baseline imbalances, and the examples you added in the Discussion (such as the stakeholder randomisation ceremony and the envelope methods) bring the text to life. The moderation of your earlier imbalance-as-bias language, along with the shift to "Domain 1 overall RoB," are also welcome changes.

That said, several issues remain that prevent this from meeting BMJ Open standards and the expectations of the Catalogue of Bias. I am offering some recommendations.

The strengths and limitations section still includes non-methodological claims. For example, you write "This review presents an in-depth analysis of recruitment bias sources and its mitigation strategies…" and "We suggested adaptation to the currently available risk of bias tools…" (Page 1, lines 28-35). Instead of describing the impact of your work, this section should set out concrete methodological features. For example:

Instead of: "This review presents an in-depth analysis of recruitment bias sources and its mitigation strategies…"
Consider: "Summarises sources of recruitment bias and mitigation strategies in stepped

wedge cluster randomised trials."
Instead of: "We suggested adaptation to the currently available risk of bias tools…"
Consider: "Proposes adaptations to existing RoB tools tailored to SW-CRTs."

In the Methods, the way you determine randomisation is still too permissive. You currently state: *"We ascertained random allocation if the term 'random' was mentioned in sequence generation"* (Page 5, lines 191-192). That threshold is too low.

Instead of: "We ascertained random allocation if the term 'random' was mentioned in sequence generation."
Consider: "We judged sequence generation 'random' only when methods consistent with true randomisation were reported (for example, computer-generated sequences or an independent statistician's algorithm). Where authors merely wrote that allocation was 'random' without method details, we coded 1a.1 as some concerns."

Similarly, your baseline difference assessment still relies on p values. In the Methods you write: *"...comparing the conditions either with proportions or means and standard deviations... or with p-values when available"* (Page 5, lines 197-200; also Page 6, lines 226-233 for inter-cluster differences).

Instead of: "…or with p-values when available."
Consider: "We summarised baseline balance descriptively and quantified differences using absolute standardised differences. We did not use hypothesis tests for baseline characteristics."

Your handling of 1b also needs tightening. In the Results and Discussion you write that when recruitment occurred after randomisation, *"we labelled such papers with high RoB in subdomain 1b"* (Page 8, lines 384-387). This reads as a design-triggered default rather than an assessment based on actual evidence.

Instead of: "…we labelled such papers with high RoB in subdomain 1b."
Consider: "When individual recruitment followed cluster randomisation, we rated 1b as some concerns by default. We upgraded to high risk only when staff or participants were unmasked in ways plausibly able to influence who was approached, enrolled, or analysed."

Masking criteria also remain too subjective. In your Methods you note: *"...when direct mentioning of masking... was missing, we took into account whether the allocation sequence was revealed... and whether it was possible to mask recruiting staff..."* (Page 6, lines 224-229).

Instead of: "…and whether it was possible to mask recruiting staff…"
Consider: "We coded masking strictly from explicit statements. Where masking was not reported and the intervention could not reasonably be hidden at the site level, we recorded 'no information' rather than inferring masking from context."

There are also clarity and consistency issues in the Results. For example, *"restricted randomisation was commonly used (n=11, 17%)"* (Page 9, lines 307-309) is ambiguous 17% of what? Every percentage should show n/N, and denominators should be consistent across the manuscript. Similarly, check that the switch from "62" to "63" studies as clearly randomised is reconciled across all locations.

Table 2 is captioned as Table but in the text you talk about Figure 2, the authors' Revisions file states: *"A score of 'High risk' or 'Some concerns' was considered as an indicator of potential 'risk of bias'..."* (Revisions doc, Figure 2 note). This grouping obscures the difference between these categories. The main figure should present low, some concerns, and high separately; if you want an "any risk" summary, make it a secondary panel or a supplementary figure.

Instead of: grouping high risk and some concerns in the main display.
Consider: "Show all three RoB 2.0 categories separately in the main figure, with 'any risk' presented only as an additional panel."

You also have useful allocation concealment detail central systems, envelope methods, sequence foreknowledge but it sits in the Discussion (Page 12, lines 354-371). That information belongs in the Results, ideally in a concise table showing method, independence of allocator, and sequence knowledge.

The mitigation strategies for 1b.2, currently listed in prose (Page 9, lines 299-319), would be far clearer in a table mapping each strategy to whether eligibility was objective, whether masking was achieved, and the resulting RoB judgement.

Finally, while you have softened your reliance on imbalance as a proxy for bias, the Results still state *"40 (67%) suggested differential identification and/or differential recruitment"* (Page 9, lines 299-319) without making it clear which had imbalance only and which had documented recruitment pathways. That distinction should be reported.

The one-year sampling frame also needs more frank discussion in the Limitations: recency may improve reporting but it introduces an epoch effect, and should be described as a trade-off. And your terminology around "recruitment bias," "selection bias," and "allocation concealment" still does not align with the Catalogue of Bias allocation concealment should not sit under recruitment bias, and selection bias should not be limited to 1b.2 outcomes.

Overall, the manuscript is improved and reads more clearly, but these remaining issues need some minor amendments. Thank you again for your effort.

Reviewer 3

Name Wogu, Adane

Affiliation Colorado School of Public Health, Department of Biostatistics and Informatics

Date 05-Sep-2025

COI

Dear Authors,

Thank you for thoughtfully addressing the reviewers' comments, suggestions, and recommendations. The manuscript has improved substantially, and I appreciate your diligent efforts in bringing it to this stage.

I have provided a few additional comments and minor edits on the revised version, attached here.

With best regards,

**** The reviewer provided a marked copy with additional comments. Please contact the publisher for full details. ****

VERSION 2 - AUTHOR RESPONSE

**Reviewer 1**		
		First and foremost, thank you for your time reviewing our manuscript. Your suggestions are extremely useful and helped us to pay more attention to the elements requiring more transparency and justifications.<br><br>The wording corrections were addressed in the manuscript as such:
Lines 65-67, Page 2	Wording corrections:<br>- replace "unfeasible" with "infeasible"	*Another reason for concern is that the robustness of the trial results might be hindered, as certain crucial elements of RCTs are likely to be* ***infeasible*** *or impractical in SW-CRT (e.g., recruitment of all participants prior to the randomisation in cross-sectional designs).*
Lines 72-75, Page 1	- replace "one" with "level" or "participants"	*It is worth mentioning that simple (unrestricted) randomisation can guarantee balance on baseline characteristics only on the cluster level, but not on the* ***individual (participants) level****, particularly when sample size is small; the balancing characteristics on individual level would require the use of restricted randomisation [7].*
Lines 114-118, Page 3	- define the abbreviations: the CONSORT (Consolidated Standards of Reporting Trials)<br>- use the abbreviation "RCTs" instead of "randomised controlled trials"	*Furthermore, it was assumed that articles published in that year would potentially be conducted and reported to higher standards than earlier years due to the increased awareness and use of* ***the Consolidated Standards of Reporting Trials (CONSORT)*** *statements partly driven by high impact journals consistently insisting on the reporting of* ***RCTs*** *to these guidelines.*
Lines 154-156, Page 4	- replace "reviews" with "reviewers"<br>- replace "author" with "reviewer"	*During data extraction, any disagreements between the* ***reviewers*** *were solved in the discussion, and a third* ***reviewer*** *would be asked to be an arbitrator for any unresolved disagreements to reach a consensus.*
Line 162, Page 5	- spell RoB out: "Risk of bias, RoB"	*4) quality assessment (****Risk of Bias, RoB****)*
	- replace "low risk" with 'low risk' (green)"; "minimal" with	*RoB reporting includes a traffic-light system, where* ***'low risk' (green)*** *shows* ***minimal or no***

Lines 186-189, Page 5	“minimal or no”, “some concerns” with “‘some concerns’ (yellow/amber)”, “high risk” with “‘high risk’ (red)”<br>- replace “high risk” with “high risk of RoB”	*concerns regarding sources of bias,* ***‘some concerns’ (yellow/amber)*** *indicates one or two moderate issue or several minor ones, and* ***‘high risk’ (red)*** *signals major issues or the combination of more than two moderate ones.*
Lines 314-317, Page 9	- check the brackets in the sentence	*Among 64 included studies, 35 (55%) were deemed as* ***high risk of bias*** *[18, 21-24, 26, 28, 29, 33-35, 38, 39, 42-44, 50-52, 54, 55, 58, 60-64, 67, 69, 72, 74, 76-79], 20 (31%) as some concerns [20, 25, 27, 30, 31, 32, 37, 41, 45, 47, 48, 53, 56, 57, 66, 68, 70, 71, 75, 80], and 9 (14%) as low risk [17, 19, 36, 40, 46, 49, 59, 65, 73], see Table 2 for further information.*
Page 16-17	- standardise wording of “crossover”<br>- be uniform. Either "carry-over" or "carryover" but not both in the manuscript	The words “crossover” and “carryover” are used in the manuscript.
Lines 86-103, Page 3	Aside from language polishing...<br><br>The way **recruitment bias** and **selection bias** are defined in this paragraph may be confusing to readers, as it differs from standard usage in the epidemiological literature where **selection bias** is typically the broader concept. I suggest clarifying upfront that these are review-specific definitions and briefly explaining the rationale for reversing the conventional hierarchy. It would also help to simplify the description of processes grouped under **recruitment bias** (since randomization, allocation concealment, and masking are often treated separately) and to provide a citation to conventional usage for context. Finally, because terminology can vary, consider including a brief disclaimer such as: *“We acknowledge that these terms are sometimes used differently in the broader methodological literature; however, for the purposes of this review, we adopted the above framework for clarity in the context of cluster randomized trials.”* This would reassure readers that the choice is deliberate and not an oversight.	We now have attempted to provide additional clarifications on the language used:<br><br>*In this review, we use terms ‘recruitment bias’ and ‘selection bias’ as per the following descriptions. Although selection bias is generally considered to be a broader term [2, 13], we deemed recruitment bias in this review as a broader term, addressing any issues arising from the randomisation process and the timing of identification or recruitment of participants (i.e., any bias identified with Cochrane Risk of bias 2.0 tool (subdomain 1a and 1b)) [14]. Selection bias in this review is defined as issues arising from differential identification and recruitment of potential participants in clusters (i.e., signalling question 1b.2 in subdomain 1b) [14], for example, if recruiting staff and/or potential participants are aware of allocation status. Such awareness is likely (either consciously or subconsciously) to affect the recruitment process [14] (e.g., recruit healthier patients in the control condition, and patients with multiple comorbidities during the intervention condition). Therefore, in this review selection bias is deemed one of the components of recruitment bias. Such approach was chosen as sometimes participants are not identified and/or recruited before randomisation of clusters, and selectivity in identification and/or recruitment can become an element in the wider recruiting process. We acknowledge that these terms are sometimes*

		used differently in the broader methodological literature [2, 13]; however, for the purposes of this review, we adopted the above framework for clarity in the context of cluster randomised trials. For further information on risk of bias assessment see Risk of bias assessment.
Lines 127-134, Page 4	In the previous version, the manuscript stated:<br>*“Searches for both published and unpublished source materials were included.”*<br><br>In the current version, after revision, this was changed to:<br>*“Searches for published materials (both peer-reviewed and preprints) were conducted in four electronic databases...”*<br><br>While the clarification on published sources is appreciated, the omission of “unpublished materials” raises concern. It remains unclear whether unpublished sources were actually included. Either answer – yes or no – raises questions about the authors’ transparency in reporting their methods.	Thank you for pointing this out. We acknowledge that this was a poorly chosen wording on our behalf that have caused some confusion. Neither grey literature nor citation searching were used for searches, and the term “unpublished sources” simply refers to pre-prints. We have added an additional sentence to highlight that no “grey literature” searches were conducted. Please let us know if current version of the search process is not transparent enough.<br><br>*Searches for **published materials (both peer-reviewed and pre-prints (i.e., reviews available online but not being peer-reviewed yet**) were conducted in four electronic databases (MEDLINE (Ovid), Embase (Ovid), Cochrane Library (Wiley, 2024, Issue 1), and CINAHL Ultimate (EBSCO)). The search strategy for literature searches electronic databases was developed with use of medical subject headings initially for MEDLINE and later adapted for other databases (Supplementary Material File 2). All papers meeting the inclusion criteria (SW-CRT design and 2023 publication year) were included. **Neither grey literature search nor citation searching were conducted.***
**Reviewer 2**		
Lines 27-30, Page 1	The strengths and limitations section still includes non-methodological claims. For example, you write “This review presents an in-depth analysis of recruitment bias sources and its mitigation strategies...” and “We suggested adaptation to the currently available risk of bias tools...” (Page 1, lines 28-35). Instead of describing the impact of your work, this section should set out concrete methodological features.<br><br>For example:<br>Instead of: “This review presents an in-depth analysis of recruitment bias sources and its mitigation strategies...”<br>Consider: “Summarises sources of recruitment bias and mitigation strategies in stepped wedge cluster randomised trials.”	We are extremely grateful for your comments and suggestions as they made us look back to some of our decisions and improve not only the wording but also the standards of the manuscript. We believe that the corrections made based on your comments will strengthen our review.<br><br>• ***Summarises sources of recruitment bias and mitigation strategies in stepped-wedge cluster randomised trials.***<br><br>• ***Proposes adaptations to existing risk of bias tools tailored to stepped-wedge cluster randomised trials.***

	Instead of: “We suggested adaptation to the currently available risk of bias tools...”<br>Consider: “Proposes adaptations to existing RoB tools tailored to SW-CRTs.”	
Lines 199-209, Page 6	In the Methods, the way you determine randomisation is still too permissive. You currently state: “We ascertained random allocation if the term ‘random’ was mentioned in sequence generation” (Page 5, lines 191-192). That threshold is too low.<br><br>Instead of: “We ascertained random allocation if the term ‘random’ was mentioned in sequence generation.”<br>Consider: “We judged sequence generation ‘random’ only when methods consistent with true randomisation were reported (for example, computer-generated sequences or an independent statistician’s algorithm). Where authors merely wrote that allocation was ‘random’ without method details, we coded 1a.1 as some concerns.”	Thank you for your suggestions, we have reviewed and re-assessed some of our previously made decisions on RoB. We now use only explicit statements to assess randomisation (please see below). Using this approach led to a number of slight amendments made, mainly affecting responses to signalling questions regarding allocation concealment (for signalling question 1a.1: previously 63 papers were deemed having low RoB and 1 paper as ‘No information’; now 60 papers have low RoB, and 4 papers were deemed ‘no information’. For signalling question 1a.2: previously 10 papers were deemed high RoB, 48 papers were low RoB, and 6 papers were marked as ‘No information’. Now we have 15 papers as high RoB, 26 papers as low RoB, and 23 papers as ‘No information’).<br><br>***We judged sequence generation ‘random’ only when methods consistent with true randomisation were reported (for example, computer-generated sequences or an independent statistician’s algorithm). Where authors merely wrote that allocation was ‘random’ without method details, we coded 1a.1 as ‘no information’, automatically raising the RoB for Domain 1a to ‘Some concerns’ as default.*** *Allocation was considered concealed when the sequence was controlled by personnel independent to the enrolment process. Due to the nature of SW-CRTs, all clusters eventually reach the intervention condition; therefore, in cases when all clusters became aware of the allocation sequence at the beginning of the trial, we considered that the allocation sequence was not concealed* ***(explicit statements on when the allocation sequence was revealed, otherwise we recorded ‘no information’).***

	Similarly, your baseline difference assessment still relies on p values. In the Methods you write: "...comparing the conditions either with proportions or means and standard deviations... or with p-values when available" (Page 5, lines 197-200; also Page 6, lines 226-233 for inter-cluster differences).<br><br>Instead of: "...or with p-values when available."<br>Consider: "We summarised baseline balance descriptively and quantified differences using absolute standardised differences. We did not use hypothesis tests for baseline characteristics."	As you rightly point out, reliance on potential hypothesis tests for baseline characteristics should be avoided, however, the Cochrane risk of bias tool 2.0 mentions applicability of p-values in the assessment of baseline imbalances. Therefore, in our justifications we used the statements provided by the authors of the included papers, and – if such statements were not reported and only a reference to 'Table 1' was given – we narratively described potential baseline differences and included p-values when reported. We did not quantify differences with absolute standardised differences, and we did not calculate p-values, using the reported ones only for RoB assessment. We have added this drawback to the review's limitations.
Lines 209-215, Page 6		*Methods:* ***When assessing the baseline differences between intervention conditions, we used the following sequential approach. We used authors' statements when these were explicitly provided. When authors' statements were not provided, we examined baseline characteristics tables and narratively compared the reported summary statistics by the intervention conditions. Where p-values for potential imbalances were reported, we noted them. We did not quantify differences with absolute standardised differences. We have not compared differences between clusters in this subdomain.***
Lines 516-519, Page 15		*Discussion (limitations):* ***When p-values were reported in baseline tables, we used them to assess signalling questions 1a.3 and 1b.3 alongside with the authors' comments, acknowledging that using p-values is not recommended for differences assessment.***
Lines 235-239, Page 6	Your handling of 1b also needs tightening. In the Results and Discussion you write that when recruitment occurred after randomisation, "we labelled such papers with high RoB in subdomain 1b" (Page 8, lines 384-387). This reads as a design-triggered default rather than an assessment based on actual evidence.<br><br>Instead of: "...we labelled such papers with high RoB in subdomain 1b."<br>Consider: "When individual recruitment followed cluster randomisation, we rated 1b as some concerns by default. We upgraded to high risk only when staff or participants were unmasked in ways plausibly able	We have tightened the wording of this part to reflect your suggestion. We have also expanded the statement as the risk of bias could be not only upgraded but also downgraded to 'Low' if masking of recruiting team was achieved and no cluster imbalances were noticed.<br><br>***... when individual recruitment followed cluster randomisation, we rated 1b as some concerns by default. We upgraded to high risk only when staff or participants were unmasked in ways plausibly able to influence who was approached, enrolled, or analysed. However, in cases when masking was achieved, and cluster characteristics are balanced, we downgraded 1b to low risk of bias.***

	to influence who was approached, enrolled, or analysed.”	
Lines 239-248, Page 6-7	Masking criteria also remain too subjective. In your Methods you note: “…when direct mentioning of masking… was missing, we took into account whether the allocation sequence was revealed… and whether it was possible to mask recruiting staff…” (Page 6, lines 224-229).<br><br>Instead of: “…and whether it was possible to mask recruiting staff…” Consider: “We coded masking strictly from explicit statements. Where masking was not reported and the intervention could not reasonably be hidden at the site level, we recorded ‘no information’ rather than inferring masking from context.”	Thank you for your suggestion, we have revised our approach to align with the suggestion and updated our results, accordingly.<br><br>***When assessing the possibility of differential recruitment, we focused on whether the recruiting personnel and potential participants were aware of the allocation sequence, and in cases when allocation sequence was revealed in advance, we answered ‘Yes’ / ‘Possibly yes’ (depending on whether explicit information was provided). When direct mentioning of masking of recruiting team was missing, but it was feasible to assess that the intervention condition was hidden at the site level, we coded this ‘Possibly no’ (e.g., inclusion of all eligible participants, use of retrospective medical records, or concealed allocation sequence). Where masking was not reported and the intervention could not reasonably be hidden at the site level, we recorded ‘no information’ rather than inferring masking from context.***
	There are also clarity and consistency issues in the Results. For example, “restricted randomisation was commonly used (n=11, 17%)” (Page 9, lines 307-309) is ambiguous 17% of what? Every percentage should show n/N, and denominators should be consistent across the manuscript. Similarly, check that the switch from “62” to “63” studies as clearly randomised is reconciled across all locations.	Thank you for your comment, we have updated the manuscript and added denominators throughout.
Figure 2	Table 2 is captioned as Table but in the text you talk about Figure 2, the authors’ Revisions file states: “A score of ‘High risk’ or ‘Some concerns’ was considered as an indicator of potential ‘risk of bias’…” (Revisions doc, Figure 2 note). This grouping obscures the difference between these categories. The main figure should present low, some concerns, and high separately; if you want an “any risk” summary, make it a secondary panel or a supplementary figure.	We have revised Figure 2 to present RoB indication for each signalling question without any grouping, we have added the responses to each signalling question (Yes or Possibly yes / No or Possibly no / No information) and updated the legend.<br><br>The legend:<br>– *Indicator of potential bias*<br>– *No risk of bias.*<br>– *No information was available to allow assessment of risk of bias.*<br>– *Not applicable (a default option for Selection bias signalling question 1b.2*

	Instead of: grouping high risk and some concerns in the main display. Consider: "Show all three RoB 2.0 categories separately in the main figure, with 'any risk' presented only as an additional panel."	*in cases when recruitment was conducted prior to the randomisation)*
Table 3 (Line 342, Page 10)	You also have useful allocation concealment detail central systems, envelope methods, sequence foreknowledge but it sits in the Discussion (Page 12, lines 354-371). That information belongs in the Results, ideally in a concise table showing method, independence of allocator, and sequence knowledge.	We have added a new Table 3 to the manuscript that captures the mitigation strategies for domains 1a.2 and 1b.2 and whether these achieved masking.
	The mitigation strategies for 1b.2, currently listed in prose (Page 9, lines 299-319), would be far clearer in a table mapping each strategy to whether eligibility was objective, whether masking was achieved, and the resulting RoB judgement.	
Lines 345-348, Page 11<br><br>Lines 359-364, Page 11	Finally, while you have softened your reliance on imbalance as a proxy for bias, the Results still state "40 (67%) suggested differential identification and/or differential recruitment" (Page 9, lines 299-319) without making it clear which had imbalance only and which had documented recruitment pathways. That distinction should be reported.	We have re-written this section for clarity:<br><br>***Individual-level baseline differences were identified in 29/64 (45%) studies, while cluster-level baseline differences were identified in 26/64 (41%) studies. In 23/64 manuscripts (36%), the authors adjusted statistical models to imbalanced covariates.***<br><br>***Importantly, imbalances in baseline characteristics do not necessarily indicate problems with randomisation or recruitment, and their sole presence might not necessarily lead to a high risk of bias. Among the trials with identified baseline imbalances in either subdomain 1a or 1b (n=41/64, 64%), 17 trials demonstrated signs of differential recruitment, but only in 7 of them the authors associated imbalances with recruitment performed by unmasked research team [23,39,43,44,55,60,67].***
Lines 498-503, Page 15	The one-year sampling frame also needs more frank discussion in the Limitations: recency may improve reporting but it introduces an epoch effect, and should be described as a trade-<br>off.	We have expanded the justification to address the comment; we hope that this is to a satisfactory level:<br><br>*The search strategy was limited to only one year (2023), and although expected to demonstrate the most current practices in addressing the risk of recruitment bias, it also violates generalisability of the review results.* ***We***

		acknowledge that recency might improve reporting, however, expanding the timeframe would have required additional resources that were not at the team's disposable. Therefore, as a trade-off, we decided to limit the included timeframe to 2023 but analyse the papers in detail.
Lines 86-103, Page 3	And your terminology around "recruitment bias," "selection bias," and "allocation concealment" still does not align with the Catalogue of Bias allocation concealment should not sit under recruitment bias, and selection bias should not be limited to 1b.2 outcomes.	We have updated the definitions and their justifications for terms "recruitment bias" and "selection bias" (as also recommended by another reviewer). The wording and definitions are review-specific, and we now acknowledge the deviation from conventional utilisation of these terms:<br><br>*In this review, we use terms 'recruitment bias' and 'selection bias' as per the following descriptions. Although selection bias is generally considered to be a broader term [2, 13], we deemed recruitment bias in this review as a broader term, addressing any issues arising from the randomisation process and the timing of identification or recruitment of participants (i.e., any bias identified with Cochrane Risk of bias 2.0 tool (subdomain 1a and 1b)) [14]. Selection bias in this review is defined as issues arising from differential identification and recruitment of potential participants in clusters (i.e., signalling question 1b.2 in subdomain 1b) [14], for example, if recruiting staff and/or potential participants are aware of allocation status. Such awareness is likely (either consciously or subconsciously) to affect the recruitment process [14] (e.g., recruit healthier patients in the control condition, and patients with multiple comorbidities during the intervention condition). Therefore, in this review selection bias is deemed one of the components of recruitment bias. Such approach was chosen as sometimes participants are not identified and/or recruited before randomisation of clusters, and selectivity in identification and/or recruitment can become an element in the wider recruiting process. We acknowledge that these terms are sometimes used differently in the broader methodological literature [2, 13]; however, for the purposes of this review, we adopted the above framework for clarity in the context of cluster randomised trials. For further information on risk of bias assessment see Risk of bias assessment.*
**Reviewer 3**		

	Throughout, I would appreciate that if the authors report a percentage, that they include a denominator along with their reported frequency. There are several places throughout the text where I tried to back-calculate the percentage based on the reported frequency and my assumption of the denominator, only to obtain a percentage that did not match with the one reported.	Thank you for your comment, we have updated the manuscript and added denominators throughout.
Lines 217-219, Page 6	Page 5, lines 203-204: "It includes an assessment of whether the randomization of clusters was performed prior to or after randomization…" I think one of the randomizations in this sentence is a typo.	It was a typo, corrected now:<br><br>*It includes an assessment of whether the randomisation of clusters was performed prior to or after* ***recruitment****…*
Lines 314-317, Page 9	Page 8, lines 298-299: There is an internal reference error	Thank you for spotting this, should be corrected now.
Lines 331-333, Page 10	Page 9, lines 307-309: The types of randomization scheme reported only account for 20% of those studies that reported their randomization allocation process; what types of randomization methods were used for the other 80% of studies?	The type of randomisation was often not reported, just general phrases like "computer-generated sequence was used". We have reviewed and updated this section in the manuscript.<br><br>***The type of randomisation was specified in 13 trials. Where reported, restricted randomisation was commonly used (n=11/64, 17%); simple (n=1/64, 1.5%) and matched-pair (n=1/64, 1.5%) were also reported.***
Lines 430-438, Page 13	Page 9, lines 311-312: I would be interested to see a breakdown of the sampling scheme (closed-cohort vs cross-sectional/open-cohort) of studies that recruited participants after randomization. I think this would allow us to see if most of these studies were prone to this bias by choice of design (such as in the case of a cross-sectional scheme), or if they were disregarding best practices (such as in the case of a closed-cohort design where clusters are randomized, but the cohort within the clusters is not recruited until after randomization occurs).	Thank you for your suggestion, we have added this information to the manuscript.<br><br>***Due to the trial design, the recruitment of all participants prior to randomisation (signalling questions 1b.1) was challenging and often infeasible due to the sampling scheme. Only three trials used the closed-cohort design [17, 27, 66], and in all of them all participants were enrolled during the recruitment stage and followed-up longitudinally for the length of a study, whereas two of the trials [37, 59] did not recruit individual participants. Fifty-six trials (56/64, 86%) had a cross-sectional or open-cohort design which meant participants were identified/recruited after randomisation. Therefore, the risk of bias could not be avoided due to the sampling strategy in these studies. Three papers were closed cohorts [51, 64, 73], however, no explicit information on when randomisation was performed in relation to recruitment.***

Lines 345-348, Page 11	Page 9, lines 322-323: If possible, I would appreciate a breakdown in why these 40 studies had a suggestion of differential identification and/or differential recruitment of individuals to clusters. Was it solely based on differences in (individual-level) baseline characteristics? Differences in cluster sizes?	We have re-written this section for clarity:<br><br>***Individual-level baseline differences were identified in 29/64 (45%) studies, while cluster-level baseline differences were identified in 26/64 (41%) studies. In 23/64 manuscripts (36%), the authors adjusted statistical models to imbalanced covariates.***
	Page 10, lines 335-341: I'm unclear on where the percentages from the current review are coming from, as they don't line up with what is reported in Table 2 (they all seem to be off by +/- 2%).	Thank you for spotting this, we have reviewed and corrected where needed all figures and percentages throughout the manuscript.
Lines 345-348, Page 11	Page 11, lines 412-415: You report thar 40 studies had imbalance on baseline characteristics, but report on page 9 (line 310) that 25 studies were identified as having individual-level baseline differences. Are these two referring to different concepts? Could the 40 mentioned in the discussion be referring to the 40 studies on lines 322-323 that suggested differential identification and/or differential recruitment of individuals to clusters?	We have re-written this section for clarity:<br><br>***Individual-level baseline differences were identified in 29/64 (45%) studies, while cluster-level baseline differences were identified in 26/64 (41%) studies. In 23/64 manuscripts (36%), the authors adjusted statistical models to imbalanced covariates.***

VERSION 3 - REVIEW

Reviewer 2

Name Kolokythas, Nico

Affiliation University of Wolverhampton, Nico Kolokythas

Date 23-Oct-2025

COI

Thank you for your efforts to refine your manuscript. I have thoroughly enjoyed the process.

Good luck with your work
